# Supplementary material for: Temperature modulates immune gene expression in mosquitoes during arbovirus infection
Source: Open Biol. 2021 Jan 6;11(1):200246. doi: 10.1098/rsob.200246 (PMC7881175; doi:10.1098/rsob.200246)
Supplement: Supplementary Figures and Tables [file rsob200246supp1.pdf]

Wimalasiri-Yapa B. M. C. R., Barrero R. A., Stassen L., Hafner L. M., McGraw E. A., Pyke A. T., Jansen C. C., Suhrbier A., Yakob L., Hu W., Devine G. J., Frentiu F. D. Temperature modulates immune gene expression in mosquitoes during arbovirus infection. ***Open Biology***. https://doi.org/10.1098/rsob.200246

Table of contents-Supplementary documents

Supplementary Figure 1. A) Volcano plot of gene expression of cases and controls at 3 dpi 18 ^{o}C, B) Volcano plot of gene expression of cases and controls at 3 dpi 32 ^{o}C, C) Volcano plot of gene expression of cases and controls at 3 dpi 28 ^{o}C, D) Heat map of gene expression of cases and controls at 3 dpi 18 ^{o}C, E) Heat map of gene expression of cases and controls at 3 dpi 32 ^{o}C, F) Heat map of gene expression of cases and controls at 3 dpi 28 ^{o}C. 2

Supplementary Figure 2. A) Volcano plot of gene expression of cases and controls at 7 dpi 18 ^{o}C, B) Volcano plot of gene expression of cases and controls at 7 dpi 32 ^{o}C, C) Volcano plot of gene expression of cases and controls at 7 dpi 28 ^{o}C, D) Heat map of gene expression of cases and controls at 7 dpi 18 ^{o}C, E) Heat map of gene expression of cases and controls at 7 dpi 32 ^{o}C, F) Heat map of gene expression of cases and controls at 7 dpi 28 ^{o}C. 3

Supplementary Figure 3. PCA plots of gene expression of infected (cases) and uninfected (controls) mosquitoes at A) 3 dpi 18 ^{o}C, B)3 dpi, 28 ^{o}C, C) 3 dpi 32 ^{o}C, D) 7 dpi 18 ^{o}C, B)7 dpi, 28 ^{o}C, C) 7 dpi 32 ^{o}C……………………………………………………………………...4

Supplementary Table 1. Mapping statistics..5

Supplementary Table 2. All DEG lists. 8

Supplementary Table 3. Number of DEGs...42

Supplementary Table 4. Classical and non-classical immune gene families and number of genes identified. 43

Supplementary Table 5. Classical immune genes. 45

Supplementary Table 6. Non-classical immune genes...57

Supplementary Table 7. Percent contribution of various gene ontologies across the temperatures 65

Supplementary Table 8. Percent contribution of various pathways across the temperatures 71

Supplementary Table 9. Genes unmapped to DAVID cloud map. 77

Supplementary Table 10. LncRNA 81

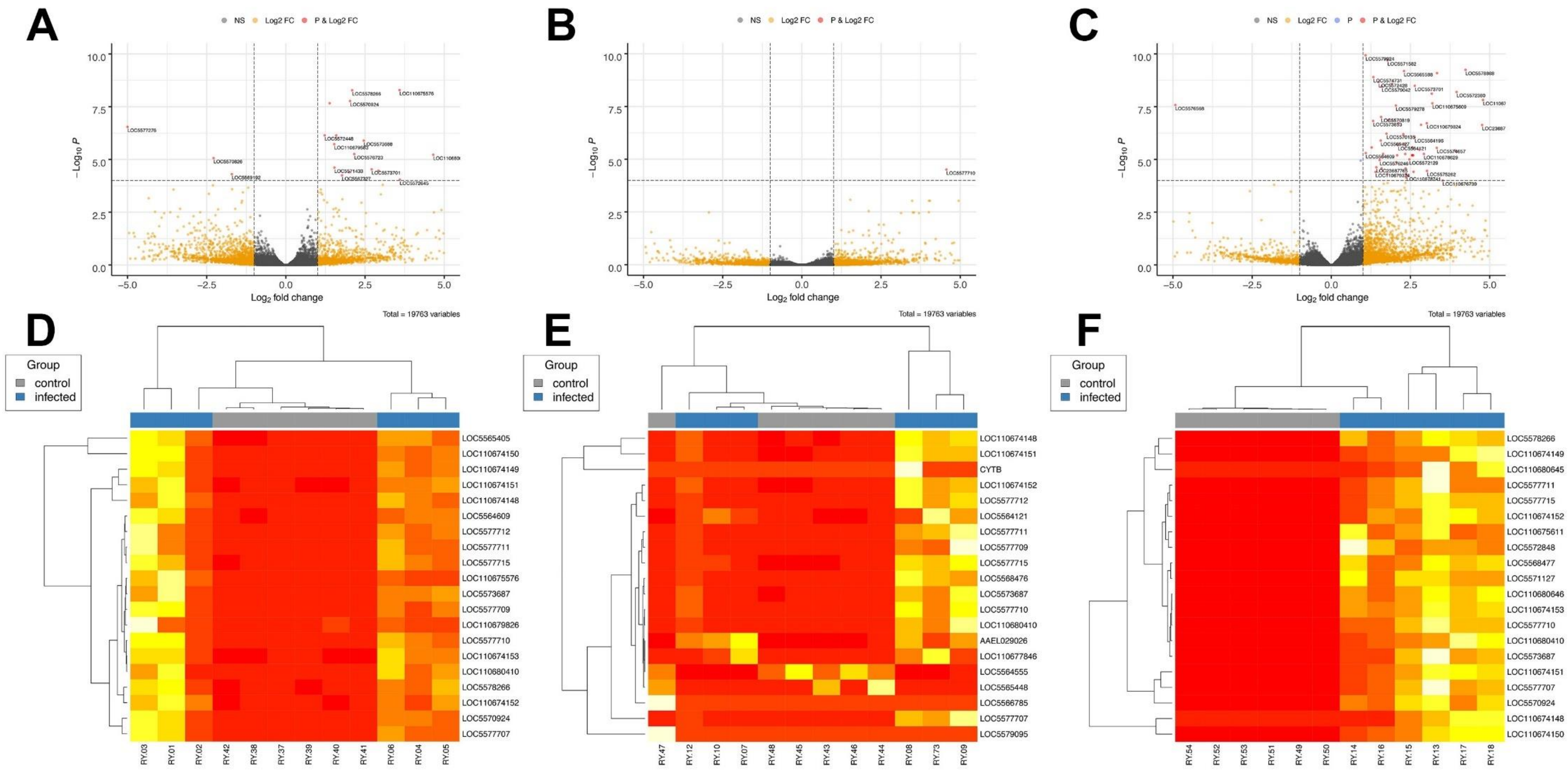

Supplementary Figure 1. A) Volcano plot of gene expression of cases and controls at 3 dpi 18 ^{o}C, B) Volcano plot of gene expression of cases and controls at 3 dpi 32 ^{o}C, C) Volcano plot of gene expression of cases and controls at 3 dpi 28 ^{o}C, D) Heat map of gene expression of cases and controls at 3 dpi 18 ^{o}C, E) Heat map of gene expression of cases and controls at 3 dpi 32 ^{o}C, F) Heat map of gene expression of cases and controls at 3 dpi 28 ^{o}C.

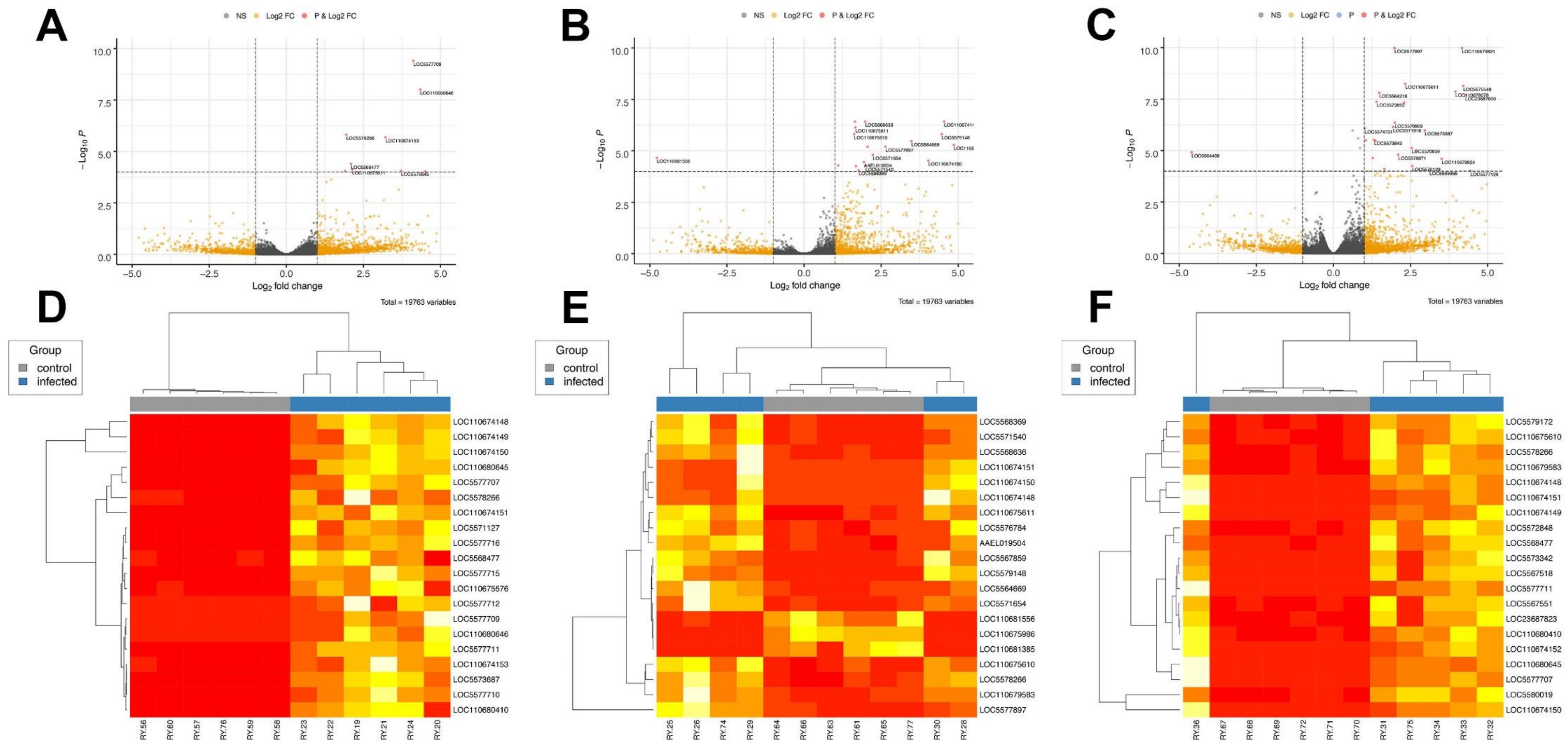

Supplementary Figure 2. A) Volcano plot of gene expression of cases and controls at 7 dpi 18 °C, B) Volcano plot of gene expression of cases and controls at 7 dpi 32 °C, C) Volcano plot of gene expression of cases and controls at 7 dpi 28 °C, D) Heat map of gene expression of cases and controls at 7 dpi 18 °C, E) Heat map of gene expression of cases and controls at 7 dpi 32 °C, F) Heat map of gene expression of cases and controls at 7 dpi 28 °C.

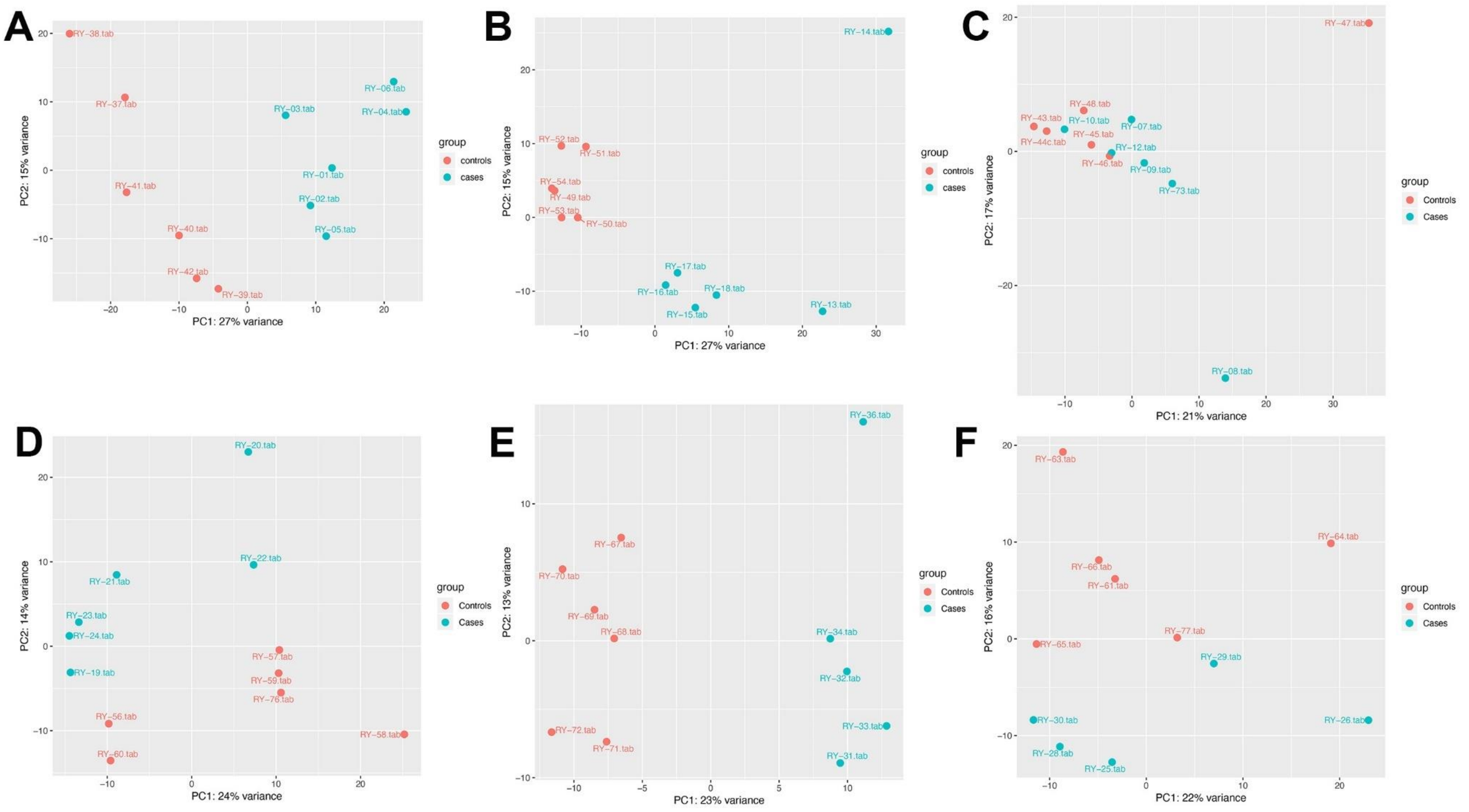

Supplementary Figure 3. PCA plots of gene expression of infected (cases) and uninfected (controls) mosquitoes at A) 3 dpi 18 ^{o}C, B)3 dpi, 28 ^{o}C, C) 3 dpi 32 ^{o}C, D) 7 dpi 18 ^{o}C, B)7 dpi, 28 ^{o}C, C) 7 dpi 32 ^{o}C.

Supplementary Table 1. Mapping statistics.

Sample ID	Raw reads R1	Raw reads R2	Mapped read pairs	% of mapped reads
**RY-01**	40,285,352	40,285,352	37,623,151	93.39
**RY-02**	38,908,531	38,908,531	36,441,243	93.66
**RY-03**	42,009,688	42,009,688	39296180	93.54
**RY-04**	42,984,040	42,984,040	40141389	93.39
**RY-05**	46,411,651	46,411,651	43128300	92.93
**RY-06**	37,002,005	37,002,005	34379255	92.91
**RY-07**	34,130,936	34,130,936	31,994,356	93.74
**RY-08**	32,190,705	32,190,705	30047114	93.34
**RY-09**	37,578,836	37,578,836	35252656	93.81
**RY-10**	38,453,304	38,453,304	36101459	93.88
**RY-12**	39,543,441	39,543,441	37166365	93.99
**RY-13**	41,962,994	41,962,994	39394169	93.88
**RY-14**	33,822,121	33,822,121	30787910	91.03
**RY-15**	32,192,708	32,192,708	29,206,064	90.72
**RY-16**	37,765,250	37,765,250	34188323	90.53
**RY-17**	38,591,247	38,591,247	35751876	92.64
**RY-18**	40,984,117	40,984,117	38603474	94.19
**RY-19**	37,091,930	37,091,930	34923378	94.15
**RY-20**	37,073,879	37,073,879	34809347	93.89
**RY-21**	38,291,431	38,291,431	35914033	93.79
**RY-22**	43,858,575	43,858,575	41268848	94.1
**RY-23**	30,765,242	30,765,242	28,836,103	93.73
**RY-24**	30,693,689	30,693,689	28,845,836	93.98
**RY-25**	38,692,441	38,692,441	36,421,995	94.13
**RY-26**	35,516,428	35,516,428	33,342,457	93.88
**RY-28**	38,218,083	38,218,083	35,909,209	93.96
**RY-29**	30,004,369	30,004,369	28200236	93.99
**RY-30**	38,562,521	38,562,521	36,298,967	94.13
**RY-31**	35,832,268	35,832,268	33,766,480	94.23

**RY-32**	35,417,713	35,417,713	33,403,827	94.31
**RY-33**	45,325,872	45,325,872	42,911,373	94.67
**RY-34**	42,575,000	42,575,000	40,117,155	94.23
**RY-36**	30,835,998	30,835,998	29,191,850	94.67
**RY-37**	34,601,897	34,601,897	32,717,138	94.55
**RY-38**	37,350,308	37,350,308	35,344,436	94.63
**RY-39**	39,612,660	39,612,660	37,360,181	94.31
**RY-40**	41,740,294	41,740,294	39,480,900	94.59
**RY-41**	36,497,253	36,497,253	34,491,830	94.51
**RY-42**	40,526,256	40,526,256	38,210,454	94.29
**RY-43**	40,632,982	40,632,982	38,492,323	94.73
**RY-44**	34,957,475	34,957,475	32,910,879	94.15
**RY-45**	35,231,754	35,231,754	33,230,913	94.32
**RY-46**	33,628,624	33,628,624	31,731,002	94.36
**RY-47**	46,187,306	46,187,306	38,243,073	82.8
**RY-48**	39,147,303	39,147,303	36,894,388	94.25
**RY-49**	32,333,451	32,333,451	30,061,200	92.97
**RY-50**	31,383,630	31,383,630	29,628,090	94.41
**RY-51**	34,759,953	34,759,953	32,787,103	94.32
**RY-52**	37,588,740	37,588,740	35352578	94.05
**RY-53**	40,571,112	40,571,112	38250283	94.28
**RY-54**	57,388,878	57,388,878	54,162,441	94.38
**RY-56**	40,163,339	40,163,339	37755106	94
**RY-57**	38,051,883	38,051,883	35830088	94.16
**RY-58**	36,193,477	36,193,477	34102310	94.22
**RY-59**	35,544,173	35,544,173	32,389,236	91.12
**RY-60**	44,312,210	44,312,210	40314320	90.98
**RY-61**	36,649,859	36,649,859	33,445,721	91.26
**RY-63**	36,481,409	36,481,409	33273723	91.21
**RY-64**	36,567,180	36,567,180	33,403,693	91.35
**RY-65**	36,197,587	36,197,587	33,021,251	91.23
**RY-66**	37,734,511	37,734,511	34,319,093	90.95
**RY-67**	35,750,054	35,750,054	32,682,290	91.42
**RY-68**	32,756,364	32,756,364	29,426,665	89.83

**RY-69**	36,849,343	36,849,343	33,560,238	91.07
**RY-70**	40,083,413	40,083,413	36,610,308	91.34
**RY-71**	34,061,633	34,061,633	31,078,074	91.24
**RY-72**	38,569,119	38,569,119	35,299,390	91.52
**RY-73**	32,810,943	32,810,943	30,310,061	92.38
**RY-74**	30,112,259	30,112,259	28,008,286	93.01
**RY-75**	29,952,637	29,952,637	28,126,155	93.9
**RY-76**	33,478,029	33,478,029	31,259,937	93.37
**RY-77**	36,205,058	36,205,058	33,836,239	93.46
**Total**	2,696,236,721	2,696,236,721	2,511,065,774	93.13

Supplementary Table 2. All DEG lists.

3 dpi 18 ^{o}C upregulated

GeneID	Base mean	log2(FC)	StdErr	Wald-Stats	P-value	P-adj
AAEL020330	3734.941	5.535313	0.380164	14.56031	5.02E-48	6.17E-44
AAEL017976	12407.69	5.247997	0.368873	14.22711	6.22E-46	3.82E-42
AAEL013346	1482.01	5.139391	0.383931	13.38625	7.28E-41	2.98E-37
AAEL013345	2014.426	4.956533	0.402408	12.31717	7.32E-35	2.25E-31
AAEL013350	6345.489	4.609722	0.398557	11.56602	6.13E-31	1.50E-27
AAEL013348	654.2395	4.427207	0.392608	11.27639	1.72E-29	3.51E-26
AAEL013351	1373.552	3.823111	0.343999	11.11371	1.08E-28	1.89E-25
AAEL013339	296.5337	4.26091	0.415188	10.26262	1.04E-24	1.59E-21
AAEL013349	1012.989	3.979345	0.396856	10.02717	1.16E-23	1.58E-20
AAEL023321	205.6866	3.799456	0.381041	9.971254	2.04E-23	2.50E-20
AAEL017975	21388.26	3.856276	0.406555	9.485246	2.42E-21	2.70E-18
AAEL026300	452.9175	3.817729	0.42868	8.905779	5.30E-19	5.42E-16
AAEL022253	11494.01	3.49052	0.422974	8.252319	1.55E-16	1.47E-13
AAEL003505	2500.659	2.027496	0.25007	8.107707	5.16E-16	4.52E-13
AAEL014531	1387.135	2.153974	0.279854	7.696777	1.40E-14	1.14E-11
AAEL014843	39193.04	1.301246	0.17041	7.635994	2.24E-14	1.72E-11
AAEL022079	771.3579	2.768739	0.364815	7.589426	3.21E-14	2.32E-11
AAEL013344	3116.273	2.221841	0.299912	7.40831	1.28E-13	7.95E-11
AAEL017380	1445.159	3.169652	0.427951	7.406578	1.30E-13	7.95E-11
AAEL001800	742.0151	1.166323	0.161765	7.20996	5.60E-13	3.27E-10
AAEL026751	8475.504	1.810765	0.268794	6.736626	1.62E-11	9.04E-09
AAEL004090	1268.531	1.454383	0.227733	6.386355	1.70E-10	8.69E-08
AAEL019935	7915.363	0.665429	0.106208	6.265334	3.72E-10	1.83E-07
AAEL027610	4012.713	2.669849	0.430476	6.202079	5.57E-10	2.63E-07
AAEL024512	229.4591	2.603068	0.426477	6.103648	1.04E-09	4.55E-07
AAEL001682	232.9808	1.234114	0.204104	6.046488	1.48E-09	6.27E-07
AAEL010680	158.535	2.096026	0.356294	5.882859	4.03E-09	1.60E-06
AAEL006883	2732.59	2.196196	0.377811	5.812946	6.14E-09	2.31E-06
AAEL001857	489.1288	2.338409	0.409105	5.71591	1.09E-08	3.94E-06
AAEL012712	384.0227	2.204887	0.391418	5.633081	1.77E-08	5.81E-06
AAEL002969	1497.899	1.8545	0.32938	5.630278	1.80E-08	5.81E-06
AAEL026833	1068.714	1.7364	0.319538	5.434093	5.51E-08	1.65E-05
AAEL000850	353.5365	1.258177	0.233109	5.397374	6.76E-08	1.98E-05
AAEL021614	20.26452	2.173809	0.406643	5.345736	9.01E-08	2.57E-05
AAEL008953	13844.17	1.054405	0.198955	5.29972	1.16E-07	3.24E-05
AAEL015609	36.04469	1.423921	0.270591	5.262256	1.42E-07	3.88E-05
AAEL013314	912.1895	0.946075	0.186049	5.085087	3.67E-07	9.60E-05
AAEL023591	2862.812	1.315018	0.258998	5.077333	3.83E-07	9.79E-05
AAEL011708	28081.34	1.088194	0.216052	5.036723	4.74E-07	0.000119
AAEL011371	3830.119	1.05284	0.211939	4.967662	6.78E-07	0.000166
AAEL008007	320.1926	1.764049	0.356803	4.944044	7.65E-07	0.000184
AAEL005558	5998.823	0.695489	0.143783	4.837075	1.32E-06	0.0003

GeneID	Base mean	log2(FC)	StdErr	Wald-Stats	P-value	P-adj
AAEL005992	93.65065	1.464637	0.304043	4.817201	1.46E-06	0.000325
AAEL022059	6900.654	2.071132	0.432829	4.785107	1.71E-06	0.000368
AAEL010411	4533.409	0.725523	0.154427	4.698174	2.62E-06	0.000537
AAEL004589	11091.43	0.688054	0.146272	4.703938	2.55E-06	0.000537
AAEL008622	84.70919	1.799117	0.382886	4.698839	2.62E-06	0.000537
AAEL025126	1084.982	2.039839	0.434553	4.694109	2.68E-06	0.000539
AAEL008473	3439.312	1.82938	0.390621	4.683258	2.82E-06	0.000559
AAEL025531	346.7837	2.01641	0.431726	4.670576	3.00E-06	0.000585
AAEL006794	4156.364	0.991009	0.216748	4.57217	4.83E-06	0.000898
AAEL021302	477.8883	1.312448	0.29467	4.453957	8.43E-06	0.001418
AAEL026519	176.072	1.193048	0.269628	4.424789	9.65E-06	0.001601
AAEL013352	325.8598	1.829224	0.415108	4.406623	1.05E-05	0.001674
AAEL023746	464.3846	0.921624	0.213034	4.326193	1.52E-05	0.002299
AAEL003632	57.25144	1.830763	0.429099	4.266529	1.99E-05	0.002769
AAEL019902	1538.573	1.347703	0.317675	4.242402	2.21E-05	0.00305
AAEL026008	3566.907	1.412411	0.334227	4.225901	2.38E-05	0.003182
AAEL026215	2918.491	0.870748	0.206926	4.208014	2.58E-05	0.0034
AAEL003619	1726.592	1.540189	0.372122	4.138936	3.49E-05	0.004326
AAEL004148	5155.337	0.586117	0.141692	4.136556	3.53E-05	0.004328
AAEL012255	163.6476	1.475474	0.361034	4.086798	4.37E-05	0.005162
AAEL014316	72.6363	0.858819	0.212107	4.048996	5.14E-05	0.005957
AAEL009055	738.5938	1.152196	0.288026	4.000323	6.33E-05	0.00719
AAEL005032	2305.857	1.064697	0.267315	3.982926	6.81E-05	0.007666
AAEL005656	472.1923	1.496157	0.376347	3.97547	7.02E-05	0.007754
AAEL014363	17.91429	1.680746	0.42306	3.972832	7.10E-05	0.007754
AAEL000812	490.3249	0.68961	0.173908	3.96538	7.33E-05	0.007754
AAEL006069	411.6914	0.794996	0.201135	3.952552	7.73E-05	0.007976
AAEL001565	231.7231	0.612403	0.155297	3.943432	8.03E-05	0.008216
AAEL008101	638.0649	0.739177	0.188032	3.931132	8.45E-05	0.008496
AAEL000338	1346.458	1.398064	0.357216	3.913773	9.09E-05	0.008852
AAEL008607	3573.331	1.274558	0.326845	3.89958	9.64E-05	0.009172
AAEL015631	1736.381	1.266749	0.32516	3.895768	9.79E-05	0.009172
AAEL008050	548.0128	1.676839	0.431892	3.88254	0.000103	0.009262
AAEL013972	12.31966	1.600317	0.412581	3.878792	0.000105	0.009338
AAEL020957	1177.887	0.666941	0.172305	3.870702	0.000109	0.009503
AAEL002655	403.1663	1.658564	0.429288	3.86352	0.000112	0.009527
AAEL012431	601.0768	1.046079	0.270886	3.861703	0.000113	0.009532
AAEL020777	7.414424	1.639095	0.426048	3.847211	0.000119	0.009976
AAEL001675	38.09614	1.598547	0.416466	3.838359	0.000124	0.01018
AAEL022900	41.52985	1.583063	0.412721	3.835668	0.000125	0.01018
AAEL000709	10552.29	0.755919	0.197674	3.824069	0.000131	0.010601
AAEL022363	131.5313	0.944937	0.255111	3.704022	0.000212	0.016018
AAEL006533	14.95517	1.510581	0.407395	3.707903	0.000209	0.016018
AAEL003888	2821.622	1.213512	0.327854	3.701374	0.000214	0.016018
AAEL001367	623.7485	0.801681	0.217583	3.68448	0.000229	0.016744

GeneID	Base mean	log2(FC)	StdErr	Wald-Stats	P-value	P-adj
AAEL008227	791.4774	0.802876	0.218254	3.678637	0.000234	0.017031
AAEL007826	3991.395	0.604745	0.165168	3.661386	0.000251	0.017903
AAEL025532	78.26468	1.325002	0.362452	3.65566	0.000257	0.018201
AAEL000251	63.28877	1.063487	0.293342	3.625423	0.000288	0.019765
AAEL010375	13.0691	1.571183	0.435826	3.605069	0.000312	0.020755
AAEL006449	2740.912	0.621239	0.172353	3.604453	0.000313	0.020755
AAEL004935	8.52101	1.511901	0.420177	3.598248	0.00032	0.020918
AAEL023999	15.31619	1.463008	0.406932	3.595211	0.000324	0.021051
AAEL023745	526.295	1.096241	0.305137	3.592623	0.000327	0.02109
AAEL007233	195.8894	1.517417	0.422445	3.591989	0.000328	0.02109
AAEL003345	14584	1.168739	0.327088	3.573165	0.000353	0.022525
AAEL025894	37.77207	1.508803	0.427931	3.525805	0.000422	0.025783
AAEL002959	465.9473	1.474204	0.419327	3.515641	0.000439	0.026658
AAEL010068	2102.199	1.052314	0.299589	3.512524	0.000444	0.02684
AAEL006197	99.02639	0.795746	0.226668	3.510627	0.000447	0.026874
AAEL024540	312.1506	0.931306	0.267558	3.480759	0.0005	0.029016
AAEL018304	124.587	1.104567	0.317662	3.477173	0.000507	0.029066
AAEL018301	658.8098	0.654417	0.188382	3.473882	0.000513	0.029288
AAEL007619	417.9272	0.977578	0.281919	3.467591	0.000525	0.029681
AAEL000750	2885.777	0.742536	0.214199	3.466577	0.000527	0.029681
AAEL013347	1493.641	1.061791	0.306269	3.466861	0.000527	0.029681
AAEL005890	1374.858	0.608056	0.175604	3.462649	0.000535	0.029961
AAEL020236	3572.704	0.670662	0.194072	3.455743	0.000549	0.030344
AAEL024560	88.94283	1.043001	0.302643	3.446309	0.000568	0.030874
AAEL026868	21.72797	1.494461	0.434361	3.440594	0.00058	0.031249
AAEL010166	29.17861	1.482285	0.431462	3.435495	0.000591	0.031567
AAEL015557	212.8244	0.90356	0.264156	3.420554	0.000625	0.032783
AAEL008213	90.29106	0.89186	0.26122	3.414204	0.00064	0.033012
AAEL010656	1644.107	1.161836	0.340652	3.410627	0.000648	0.033012
AAEL020575	74.75739	1.384464	0.407632	3.396357	0.000683	0.034081
AAEL019995	1158.313	0.785758	0.231543	3.393564	0.00069	0.034285
AAEL026878	1423.348	1.41347	0.417999	3.381516	0.000721	0.034837
AAEL003003	122.3534	0.73441	0.217367	3.378668	0.000728	0.035062
AAEL014335	634.6688	0.78638	0.233875	3.362395	0.000773	0.036383
AAEL021595	155.5841	0.583431	0.173379	3.365067	0.000765	0.036383
AAEL018241	157.9798	1.265813	0.376281	3.364006	0.000768	0.036383
AAEL011412	460.3259	0.711086	0.211569	3.361015	0.000777	0.036383
AAEL012853	230.0456	1.288257	0.383079	3.362902	0.000771	0.036383
AAEL019637	1155.443	1.089482	0.328978	3.311713	0.000927	0.04139
AAEL028247	71.85983	1.245511	0.377009	3.30366	0.000954	0.04229
AAEL011836	411.2745	0.76168	0.230797	3.300221	0.000966	0.042657
AAEL018189	31.04136	1.295138	0.395846	3.271821	0.001069	0.045075
AAEL004124	575.2964	0.742328	0.228034	3.255346	0.001133	0.046495
AAEL013974	532.2355	0.87561	0.269554	3.248369	0.001161	0.047334
AAEL026194	18.47451	1.373897	0.423511	3.244064	0.001178	0.04768

GeneID	Base mean	log2(FC)	StdErr	Wald-Stats	P-value	P-adj
AAEL006362	3303.221	0.58143	0.179262	3.243469	0.001181	0.04768
AAEL001498	393.9792	1.215518	0.375145	3.240125	0.001195	0.047772
AAEL027096	178.8959	0.848482	0.261846	3.240379	0.001194	0.047772
AAEL002075	27.44772	1.093068	0.337582	3.23793	0.001204	0.047984
AAEL014910	139.7114	0.959766	0.298058	3.220062	0.001282	0.049551
AAEL009038	2123.108	0.809267	0.25118	3.22186	0.001274	0.049551
AAEL014348	306.4843	0.895364	0.278098	3.219605	0.001284	0.049551
AAEL027238	198.671	0.7071	0.219534	3.220908	0.001278	0.049551
AAEL019751	742.0433	0.88313	0.274171	3.221094	0.001277	0.049551

FC: fold change; P-adj: adjusted *p* value

3 dpi 18 ^{o}C downregulated

GeneID	Base mean	log2(FC)	StdErr	Wald-Stats	P-value	P-adj
AAEL009165	438.394	-3.08522	0.413725	-7.45717	8.84E-14	6.03E-11
AAEL007450	124.6644	-1.55767	0.239976	-6.49094	8.53E-11	4.55E-08
AAEL012766	23.81061	-2.08265	0.340855	-6.11008	9.96E-10	4.53E-07
AAEL008609	223.5211	-1.9133	0.320578	-5.96828	2.40E-09	9.81E-07
AAEL015304	766.0237	-1.18589	0.204076	-5.81103	6.21E-09	2.31E-06
AAEL023395	82.28582	-2.09976	0.370658	-5.66495	1.47E-08	5.01E-06
AAEL008602	294.326	-1.16002	0.206542	-5.6164	1.95E-08	6.10E-06
AAEL012884	640.7386	-0.79055	0.140843	-5.61299	1.99E-08	6.10E-06
AAEL024161	359.2593	-2.11474	0.429758	-4.92077	8.62E-07	0.000203
AAEL004283	222.2479	-0.81149	0.16569	-4.89766	9.70E-07	0.000225
AAEL007664	136.8199	-1.76802	0.367691	-4.80844	1.52E-06	0.000333
AAEL023753	722.3696	-1.44348	0.311751	-4.63023	3.65E-06	0.000701
AAEL001607	1074.553	-1.23204	0.268854	-4.58257	4.59E-06	0.000867
AAEL001693	180.6399	-1.83209	0.402402	-4.5529	5.29E-06	0.000969
AAEL023158	127.7007	-0.88786	0.195577	-4.53971	5.63E-06	0.001017
AAEL021138	278.383	-1.24699	0.276271	-4.51364	6.37E-06	0.001134
AAEL009214	1221.181	-1.36277	0.304488	-4.47563	7.62E-06	0.001336
AAEL012717	39.94538	-1.92836	0.432801	-4.45554	8.37E-06	0.001418
AAEL007317	180.7115	-0.84021	0.190458	-4.41152	1.03E-05	0.001658
AAEL027166	678.8159	-1.79064	0.40985	-4.36901	1.25E-05	0.001964
AAEL014864	127.8909	-1.01439	0.232407	-4.36473	1.27E-05	0.001978
AAEL004313	1195.406	-1.6177	0.371598	-4.35337	1.34E-05	0.002057
AAEL008701	223.0075	-1.81708	0.420361	-4.32266	1.54E-05	0.002308
AAEL009843	60.57035	-1.81209	0.420225	-4.31218	1.62E-05	0.002391
AAEL000500	1787.485	-1.68356	0.392061	-4.29413	1.75E-05	0.002563
AAEL003443	318.847	-1.78304	0.415707	-4.28917	1.79E-05	0.00259
AAEL013118	3375.385	-1.81879	0.424468	-4.28488	1.83E-05	0.00261
AAEL008063	581.9485	-0.61889	0.14459	-4.28034	1.87E-05	0.002633
AAEL004700	112.9155	-1.20243	0.284015	-4.2337	2.30E-05	0.003135
AAEL003601	577.9445	-1.23636	0.295569	-4.183	2.88E-05	0.003757
AAEL008876	274.1447	-1.61046	0.387061	-4.16073	3.17E-05	0.004099

GeneID	Base mean	log2(FC)	StdErr	Wald-Stats	P-value	P-adj
AAEL003100	80.70409	-1.66985	0.401941	-4.15446	3.26E-05	0.004169
AAEL024064	52.7001	-1.79892	0.433341	-4.15127	3.31E-05	0.004184
AAEL011766	48.33218	-1.05116	0.253682	-4.14363	3.42E-05	0.004282
AAEL008561	707.1124	-0.60988	0.147605	-4.13186	3.60E-05	0.004373
AAEL024926	5.064363	-1.77784	0.432335	-4.11218	3.92E-05	0.004717
AAEL010196	4565.047	-1.74898	0.425987	-4.10571	4.03E-05	0.004804
AAEL019650	534.3518	-1.15792	0.284307	-4.07277	4.65E-05	0.005431
AAEL015458	2057.041	-1.68346	0.424537	-3.96541	7.33E-05	0.007754
AAEL002904	35.40926	-1.19036	0.299887	-3.96936	7.21E-05	0.007754
AAEL012932	1730.283	-0.73613	0.185405	-3.97039	7.18E-05	0.007754
AAEL013712	1776.191	-1.71109	0.430227	-3.97719	6.97E-05	0.007754
AAEL005008	774.5354	-1.55791	0.393108	-3.96307	7.40E-05	0.007763
AAEL025199	2331.33	-1.41866	0.35822	-3.96029	7.49E-05	0.007787
AAEL000146	32.40373	-1.29028	0.327616	-3.93838	8.20E-05	0.008322
AAEL013554	76.32456	-1.57441	0.400667	-3.92947	8.51E-05	0.008496
AAEL013001	139.4364	-1.56577	0.399543	-3.91889	8.90E-05	0.008806
AAEL021086	47.59014	-1.30114	0.332338	-3.91512	9.04E-05	0.008852
AAEL003182	543.8475	-1.27316	0.3267	-3.89703	9.74E-05	0.009172
AAEL006381	741.8508	-1.66804	0.429611	-3.88267	0.000103	0.009262
AAEL017056	5.354633	-1.69349	0.435699	-3.88685	0.000102	0.009262
AAEL019834	509.3668	-1.44168	0.370473	-3.89147	9.96E-05	0.009262
AAEL011741	2328.323	-0.83752	0.2157	-3.88281	0.000103	0.009262
AAEL003742	287.8265	-0.9903	0.256044	-3.8677	0.00011	0.009503
AAEL010276	235.6529	-1.66376	0.430305	-3.86645	0.00011	0.009503
AAEL006903	59.28145	-1.65685	0.430071	-3.8525	0.000117	0.00983
AAEL000757	854.9099	-1.00989	0.26288	-3.84162	0.000122	0.010137
AAEL000471	759.0046	-1.48929	0.388222	-3.83619	0.000125	0.01018
AAEL008488	40.27667	-1.35145	0.35603	-3.79589	0.000147	0.011803
AAEL024475	203.5882	-1.14069	0.30366	-3.75648	0.000172	0.013646
AAEL018216	67.65922	-0.90195	0.24131	-3.73773	0.000186	0.014611
AAEL002775	124.9256	-0.69675	0.187053	-3.72487	0.000195	0.015171
AAEL003066	5406.107	-1.44144	0.387124	-3.72345	0.000197	0.015171
AAEL006615	110.1771	-1.60289	0.433354	-3.69881	0.000217	0.016018
AAEL003600	1127.051	-1.08632	0.294022	-3.6947	0.00022	0.016182
AAEL013421	111.7923	-1.29698	0.353452	-3.66946	0.000243	0.017448
AAEL012318	262.092	-1.33428	0.366013	-3.64544	0.000267	0.018758
AAEL015336	488.5393	-1.07378	0.294591	-3.64497	0.000267	0.018758
AAEL008751	69.78643	-1.30677	0.358706	-3.64302	0.000269	0.018793
AAEL024221	5.345853	-1.55643	0.429452	-3.62422	0.00029	0.019765
AAEL006347	1959.661	-1.34767	0.372936	-3.61368	0.000302	0.020361
AAEL006454	68.70137	-1.15657	0.320311	-3.61077	0.000305	0.020478
AAEL028002	9.806623	-1.28049	0.355546	-3.60148	0.000316	0.02077
AAEL004676	329.8385	-1.03017	0.285967	-3.60239	0.000315	0.02077
AAEL007668	478.7056	-1.33325	0.373241	-3.57207	0.000354	0.022525
AAEL004301	431.6955	-1.0681	0.29933	-3.56831	0.000359	0.022734

GeneID	Base mean	log2(FC)	StdErr	Wald-Stats	P-value	P-adj
AAEL004097	326.8902	-0.9176	0.257328	-3.56587	0.000363	0.022828
AAEL003806	71.00272	-1.55063	0.435198	-3.56306	0.000367	0.022957
AAEL006483	663.1591	-1.53862	0.433829	-3.54661	0.00039	0.024315
AAEL020477	634.2157	-0.86628	0.245056	-3.53505	0.000408	0.025275
AAEL006333	403.5089	-1.28681	0.364248	-3.53278	0.000411	0.025365
AAEL000223	201.1629	-1.43697	0.407537	-3.526	0.000422	0.025783
AAEL024122	49.64152	-1.25495	0.357578	-3.50958	0.000449	0.026874
AAEL015450	1765.982	-1.31602	0.375199	-3.50752	0.000452	0.026944
AAEL004728	1624.614	-1.187	0.339004	-3.50143	0.000463	0.027179
AAEL021513	118.4388	-1.04908	0.30041	-3.49217	0.000479	0.028005
AAEL003722	104.7567	-0.84764	0.243565	-3.48015	0.000501	0.029016
AAEL028236	47.74142	-1.10188	0.316866	-3.47744	0.000506	0.029066
AAEL008769	502.7905	-1.48734	0.429667	-3.4616	0.000537	0.029961
AAEL023844	208.2892	-1.01111	0.293357	-3.44669	0.000568	0.030874
AAEL004931	1059.659	-1.44743	0.420047	-3.44588	0.000569	0.030874
AAEL000885	180.1278	-0.76649	0.22249	-3.44505	0.000571	0.030874
AAEL021180	38.37013	-1.19695	0.348403	-3.43554	0.000591	0.031567
AAEL018125	45.97186	-1.43962	0.420605	-3.42274	0.00062	0.032783
AAEL007926	783.3932	-1.29407	0.378206	-3.42162	0.000623	0.032783
AAEL001674	396.8265	-1.44401	0.423314	-3.4112	0.000647	0.033012
AAEL002185	143.3106	-1.15058	0.337187	-3.4123	0.000644	0.033012
AAEL003318	646.4753	-1.37486	0.402531	-3.41554	0.000637	0.033012
AAEL013054	89.28896	-0.70108	0.205719	-3.40795	0.000655	0.0332
AAEL010318	96968.52	-0.70391	0.206996	-3.40061	0.000672	0.033964
AAEL010235	1711.07	-1.14545	0.337265	-3.39631	0.000683	0.034081
AAEL013375	6.526511	-1.446	0.42573	-3.39653	0.000682	0.034081
AAEL002801	162.5786	-1.02585	0.302658	-3.38948	0.0007	0.034377
AAEL005776	75.35789	-0.9483	0.27984	-3.38871	0.000702	0.034377
AAEL019698	13.24113	-1.36899	0.40402	-3.38843	0.000703	0.034377
AAEL026023	258.7575	-1.38667	0.409744	-3.38424	0.000714	0.03463
AAEL007907	160.1749	-0.84771	0.250468	-3.38451	0.000713	0.03463
AAEL008773	1346.675	-0.89811	0.266904	-3.36492	0.000766	0.036383
AAEL013276	45.17908	-1.33383	0.396792	-3.36154	0.000775	0.036383
AAEL000689	44.09866	-1.31763	0.392774	-3.35469	0.000795	0.037084
AAEL025667	299.8847	-1.44163	0.429911	-3.35332	0.000798	0.037126
AAEL023431	7.05051	-1.29718	0.387656	-3.34622	0.000819	0.037946
AAEL008753	509.4985	-1.42447	0.426082	-3.34319	0.000828	0.03803
AAEL009449	175.5888	-1.26647	0.38027	-3.33044	0.000867	0.039421
AAEL023874	15.27692	-1.34954	0.405863	-3.32511	0.000884	0.039974
AAEL019588	119.2231	-0.96967	0.291674	-3.3245	0.000886	0.039974
AAEL024149	5.098964	-1.42067	0.428807	-3.31308	0.000923	0.041339
AAEL012687	68.23247	-1.33568	0.405288	-3.29563	0.000982	0.043051
AAEL008424	739.1683	-0.96785	0.293938	-3.29272	0.000992	0.043161
AAEL003483	643.4096	-1.39197	0.423096	-3.28996	0.001002	0.043161
AAEL002656	402.7658	-0.99556	0.30252	-3.29088	0.000999	0.043161

GeneID	Base mean	log2(FC)	StdErr	Wald-Stats	P-value	P-adj
AAEL014830	385.1722	-1.38864	0.422694	-3.28521	0.001019	0.043738
AAEL008141	853.4426	-0.62107	0.18939	-3.2793	0.001041	0.044355
AAEL002675	193.839	-1.41964	0.43377	-3.27279	0.001065	0.045075
AAEL007669	54.41489	-1.4053	0.429402	-3.2727	0.001065	0.045075
AAEL005199	90.53596	-1.31955	0.40354	-3.26995	0.001076	0.045208
AAEL017212	7682.897	-0.71193	0.217779	-3.26905	0.001079	0.045208
AAEL021576	33.63242	-0.99791	0.305587	-3.26555	0.001093	0.045615
AAEL008860	221.8753	-0.70897	0.217207	-3.26404	0.001098	0.045703
AAEL006587	288.8305	-0.74326	0.227802	-3.26276	0.001103	0.045754
AAEL006406	1720.222	-1.0076	0.310897	-3.24096	0.001191	0.047772
AAEL017320	134.5747	-1.38428	0.428618	-3.22963	0.00124	0.049042
AAEL024146	41.99415	-1.39058	0.431907	-3.21963	0.001284	0.049551
AAEL000101	293.866	-1.01511	0.315583	-3.21662	0.001297	0.049912

FC: fold change; P-adj: adjusted *p* value

3 dpi 28 ^{o}C upregulated

GeneID	Base mean	log2(FC)	StdErr	Wald-Stats	P-value	P-adj
AAEL013350	5784.056	7.656931	0.323131	23.69607	3.96E-124	5.02E-120
AAEL013339	190.3038	6.206891	0.30999	20.02289	3.48E-89	2.21E-85
AAEL017975	7345.993	5.90406	0.333718	17.69179	4.85E-70	2.05E-66
AAEL017976	6584.064	6.062034	0.368852	16.43487	1.08E-60	3.41E-57
AAEL020330	1361.706	6.110232	0.37674	16.21868	3.72E-59	9.43E-56
AAEL013346	1155.747	5.574374	0.375043	14.86329	5.71E-50	1.21E-46
AAEL013348	421.3866	4.765501	0.331875	14.35932	9.31E-47	1.69E-43
AAEL023321	283.7587	3.400928	0.251276	13.53461	9.77E-42	1.55E-38
AAEL026215	1692.454	1.37514	0.106498	12.91241	3.83E-38	4.88E-35
AAEL022253	1987.402	4.84703	0.375388	12.91204	3.85E-38	4.88E-35
AAEL024512	174.4702	3.730291	0.31059	12.01032	3.14E-33	3.61E-30
AAEL006883	1027.71	2.569106	0.214682	11.96702	5.29E-33	5.59E-30
AAEL019751	901.0433	2.227562	0.19015	11.71475	1.07E-31	1.04E-28
AAEL027610	6435.418	4.688384	0.410716	11.41516	3.51E-30	3.18E-27
AAEL013351	1269.289	3.296215	0.30531	10.7963	3.58E-27	3.03E-24
AAEL008622	70.84556	3.009712	0.286288	10.51289	7.53E-26	5.97E-23
AAEL026751	9525.754	2.211781	0.211952	10.43528	1.71E-25	1.28E-22
AAEL025531	65.35001	3.261551	0.342989	9.509189	1.92E-21	1.35E-18
AAEL023591	2776.274	1.985301	0.210816	9.417219	4.63E-21	3.09E-18
AAEL011038	923.8275	1.967118	0.211192	9.314348	1.23E-20	7.78E-18
AAEL010068	1679.027	2.230035	0.242467	9.197262	3.67E-20	2.22E-17
AAEL026833	1376.325	2.809921	0.307319	9.143345	6.05E-20	3.49E-17
AAEL013349	800.6453	3.593085	0.393457	9.132086	6.72E-20	3.70E-17
AAEL003505	3237.838	2.219512	0.247298	8.975035	2.83E-19	1.50E-16
AAEL026008	4188.54	2.715168	0.311314	8.721637	2.74E-18	1.39E-15
AAEL006794	4168.475	1.017043	0.118469	8.584925	9.09E-18	4.43E-15
AAEL022059	3368.455	3.626993	0.423239	8.569612	1.04E-17	4.87E-15
AAEL005992	152.9939	2.341997	0.274841	8.521268	1.58E-17	7.14E-15

GeneID	Base mean	log2(FC)	StdErr	Wald-Stats	P-value	P-adj
AAEL010680	70.01603	2.522056	0.296131	8.51668	1.64E-17	7.18E-15
AAEL002467	7464.032	3.451633	0.416294	8.291341	1.12E-16	4.73E-14
AAEL003728	458.8365	2.612568	0.327448	7.978577	1.48E-15	6.05E-13
AAEL013347	1090.537	2.140365	0.275172	7.778275	7.35E-15	2.91E-12
AAEL001857	219.3792	2.584523	0.333103	7.758935	8.56E-15	3.29E-12
AAEL021302	460.6418	1.787031	0.230846	7.741223	9.85E-15	3.67E-12
AAEL001794	1467.342	1.423436	0.19009	7.488224	6.98E-14	2.53E-11
AAEL009762	310.1128	2.801133	0.380186	7.367798	1.73E-13	6.11E-11
AAEL003726	134.2793	2.865447	0.392324	7.303776	2.80E-13	9.46E-11
AAEL011371	5108.795	1.269037	0.173792	7.302035	2.83E-13	9.46E-11
AAEL004870	217.5289	1.889874	0.262189	7.208052	5.68E-13	1.84E-10
AAEL021072	57.51679	2.684856	0.3752	7.155806	8.32E-13	2.64E-10
AAEL009171	3057.94	1.660286	0.232613	7.137558	9.50E-13	2.94E-10
AAEL015631	1068.381	1.803133	0.260341	6.926052	4.33E-12	1.31E-09
AAEL017380	161.5912	2.839763	0.413419	6.868969	6.47E-12	1.86E-09
AAEL025126	274.4642	2.810271	0.415808	6.758581	1.39E-11	3.93E-09
AAEL013984	1618.659	1.47697	0.219324	6.734186	1.65E-11	4.54E-09
AAEL021795	4203.723	2.428758	0.361348	6.721388	1.80E-11	4.85E-09
AAEL007344	902.5024	2.380682	0.364705	6.527694	6.68E-11	1.76E-08
AAEL028247	37.18132	2.325267	0.356505	6.522391	6.92E-11	1.79E-08
AAEL012567	51.72097	2.160349	0.33344	6.478975	9.23E-11	2.34E-08
AAEL002853	414.3521	1.426999	0.222454	6.414796	1.41E-10	3.50E-08
AAEL005903	337.8735	1.939593	0.305302	6.353036	2.11E-10	5.15E-08
AAEL002610	2168.662	1.641394	0.261188	6.284342	3.29E-10	7.87E-08
AAEL006795	321.4665	1.841573	0.29576	6.226574	4.77E-10	1.12E-07
AAEL013352	798.3605	2.470338	0.398806	6.19434	5.85E-10	1.35E-07
AAEL002655	142.1698	2.037876	0.33391	6.10307	1.04E-09	2.36E-07
AAEL008607	3804.703	1.504206	0.247252	6.083701	1.17E-09	2.61E-07
AAEL010769	935.7548	1.240058	0.206443	6.006778	1.89E-09	4.14E-07
AAEL014531	905.2883	1.084319	0.180737	5.999426	1.98E-09	4.25E-07
AAEL009645	6326.606	1.186614	0.198253	5.985353	2.16E-09	4.49E-07
AAEL017334	4229.773	1.92533	0.321629	5.986191	2.15E-09	4.49E-07
AAEL019564	531.9792	1.464263	0.244865	5.97989	2.23E-09	4.57E-07
AAEL022079	181.5742	2.163054	0.362815	5.961863	2.49E-09	5.02E-07
AAEL009952	172.918	1.50934	0.255248	5.913221	3.35E-09	6.64E-07
AAEL014541	397.9313	2.022085	0.346435	5.836838	5.32E-09	1.04E-06
AAEL005428	6536.217	2.116166	0.362993	5.829777	5.55E-09	1.07E-06
AAEL012712	227.1891	1.745037	0.299754	5.821569	5.83E-09	1.10E-06
AAEL008106	812.1433	1.492137	0.257569	5.793154	6.91E-09	1.29E-06
AAEL002757	25.01229	2.039375	0.354207	5.757581	8.53E-09	1.57E-06
AAEL023999	15.79765	1.933722	0.336125	5.752984	8.77E-09	1.59E-06
AAEL002969	989.6688	2.008806	0.350082	5.738108	9.57E-09	1.71E-06
AAEL009055	512.7365	1.758351	0.306746	5.732279	9.91E-09	1.74E-06
AAEL014348	185.223	2.002745	0.349573	5.729125	1.01E-08	1.75E-06
AAEL000811	315.0489	1.503285	0.262761	5.721119	1.06E-08	1.81E-06

GeneID	Base mean	log2(FC)	StdErr	Wald-Stats	P-value	P-adj
AAEL004090	2135.773	1.759188	0.312888	5.622413	1.88E-08	3.18E-06
AAEL007624	818.8665	1.424095	0.254414	5.597555	2.17E-08	3.63E-06
AAEL006708	81.64057	1.883651	0.338754	5.560519	2.69E-08	4.43E-06
AAEL002075	44.26663	2.035739	0.367087	5.545652	2.93E-08	4.76E-06
AAEL003740	279.2502	1.904356	0.346661	5.49342	3.94E-08	6.25E-06
AAEL010411	6156.341	0.808247	0.147511	5.479229	4.27E-08	6.69E-06
AAEL006323	508.8304	1.965614	0.362572	5.421304	5.92E-08	9.15E-06
AAEL008936	564.0859	1.602409	0.296276	5.408497	6.36E-08	9.71E-06
AAEL021929	262.6133	2.041578	0.3788	5.389598	7.06E-08	1.07E-05
AAEL001100	759.5531	1.845488	0.34436	5.359183	8.36E-08	1.25E-05
AAEL001232	630.6709	1.182004	0.22068	5.356191	8.50E-08	1.25E-05
AAEL010338	122.3551	1.774577	0.33417	5.310408	1.09E-07	1.59E-05
AAEL017345	1353.901	1.301753	0.245565	5.301062	1.15E-07	1.66E-05
AAEL010076	176.9812	1.401221	0.264481	5.298	1.17E-07	1.67E-05
AAEL028635	337.1032	2.140899	0.41845	5.116264	3.12E-07	4.39E-05
AAEL001690	1042.88	2.14875	0.420905	5.105068	3.31E-07	4.61E-05
AAEL007092	1666.175	0.984136	0.194172	5.068368	4.01E-07	5.52E-05
AAEL022387	2408.535	1.8226	0.35972	5.066721	4.05E-07	5.52E-05
AAEL001929	57.63435	1.77557	0.351062	5.057718	4.24E-07	5.72E-05
AAEL001513	64.07459	1.532098	0.304763	5.027181	4.98E-07	6.64E-05
AAEL008190	362.6617	1.261397	0.254119	4.963805	6.91E-07	9.13E-05
AAEL002412	1098.94	1.340911	0.270462	4.957859	7.13E-07	9.31E-05
AAEL005432	3110.994	1.514457	0.307333	4.927742	8.32E-07	0.0001076
AAEL026819	103.2923	1.801787	0.367513	4.902648	9.46E-07	0.0001187
AAEL022334	969.9629	1.652164	0.337944	4.88887	1.01E-06	0.0001254
AAEL003978	144.74	1.303115	0.266597	4.887959	1.02E-06	0.0001254
AAEL002124	735.3393	1.026379	0.210275	4.881121	1.05E-06	0.0001286
AAEL004715	539.5686	1.268765	0.261237	4.856753	1.19E-06	0.0001441
AAEL001503	338.8052	1.266419	0.262135	4.831165	1.36E-06	0.0001623
AAEL005293	1313.266	1.92198	0.398187	4.826826	1.39E-06	0.0001643
AAEL011203	106.977	1.869969	0.389089	4.806015	1.54E-06	0.0001807
AAEL001565	124.8969	1.392703	0.290008	4.802295	1.57E-06	0.0001824
AAEL004688	2524.55	0.944062	0.199424	4.733936	2.20E-06	0.0002538
AAEL017139	187.815	1.911766	0.40473	4.723563	2.32E-06	0.0002647
AAEL027019	94.76075	1.53148	0.325302	4.707866	2.50E-06	0.0002833
AAEL010434	159.8025	1.897974	0.403811	4.700159	2.60E-06	0.0002916
AAEL006990	1028.282	1.738972	0.371248	4.684128	2.81E-06	0.0003126
AAEL008953	13309.45	1.170589	0.250402	4.674837	2.94E-06	0.0003243
AAEL026603	42.51229	1.547599	0.333241	4.644089	3.42E-06	0.0003729
AAEL012702	3369.073	1.529489	0.329453	4.642512	3.44E-06	0.0003729
AAEL029047	47.98867	1.971092	0.426172	4.625108	3.74E-06	0.0004022
AAEL019684	11.20749	1.747526	0.378185	4.620825	3.82E-06	0.0004071
AAEL008105	7217.976	0.801024	0.173472	4.617602	3.88E-06	0.0004101
AAEL026175	2198.047	1.405885	0.304609	4.615384	3.92E-06	0.000411
AAEL001800	950.5305	0.935029	0.204034	4.582722	4.59E-06	0.0004769

GeneID	Base mean	log2(FC)	StdErr	Wald-Stats	P-value	P-adj
AAEL006533	23.2452	1.638814	0.359215	4.562206	5.06E-06	0.0005217
AAEL014754	330.1854	1.491431	0.331149	4.503808	6.67E-06	0.0006769
AAEL011727	21.82691	1.887064	0.419431	4.499103	6.82E-06	0.0006865
AAEL023348	338.2233	0.868756	0.19344	4.491087	7.09E-06	0.0007073
AAEL009904	181.8715	1.884171	0.419793	4.488331	7.18E-06	0.0007109
AAEL009074	8645.459	1.019847	0.228361	4.465948	7.97E-06	0.0007833
AAEL007926	1651.043	1.639836	0.368175	4.453961	8.43E-06	0.000822
AAEL010688	172.4118	1.386629	0.31161	4.44988	8.59E-06	0.0008314
AAEL013434	330.6199	0.798817	0.180432	4.427252	9.54E-06	0.0009028
AAEL015465	220.0909	1.400984	0.316382	4.428138	9.51E-06	0.0009028
AAEL008473	2504.421	1.254764	0.284979	4.403007	1.07E-05	0.0009962
AAEL023729	325.2253	1.876554	0.426262	4.402351	1.07E-05	0.0009962
AAEL007342	2699.181	1.278658	0.29103	4.393562	1.12E-05	0.0010243
AAEL000859	1239.467	1.861165	0.425578	4.373264	1.22E-05	0.0011083
AAEL026531	121.5693	0.901493	0.206381	4.368094	1.25E-05	0.0011211
AAEL026031	67.43212	1.114144	0.255089	4.367659	1.26E-05	0.0011211
AAEL008157	167.8229	1.553542	0.35625	4.360817	1.30E-05	0.0011406
AAEL006276	179.9901	1.366195	0.313569	4.356926	1.32E-05	0.0011524
AAEL009850	96.99051	1.474627	0.338563	4.355547	1.33E-05	0.0011524
AAEL012536	832.206	1.176214	0.270873	4.342311	1.41E-05	0.0012076
AAEL017095	41.09661	1.786439	0.411297	4.343424	1.40E-05	0.0012076
AAEL002353	84.33612	1.540883	0.355015	4.340331	1.42E-05	0.0012103
AAEL003886	46.91248	1.722213	0.397482	4.332804	1.47E-05	0.0012441
AAEL002269	1148.651	1.444293	0.333561	4.329917	1.49E-05	0.0012522
AAEL010596	192.8158	1.478195	0.342404	4.317107	1.58E-05	0.0013184
AAEL012698	394.4726	1.588359	0.368058	4.315511	1.59E-05	0.0013192
AAEL021257	71.32764	1.376562	0.31922	4.312265	1.62E-05	0.0013301
AAEL023395	41.78586	1.80173	0.418197	4.308334	1.64E-05	0.0013452
AAEL027700	263.9977	1.540998	0.359122	4.29102	1.78E-05	0.0014452
AAEL007197	1251.759	0.976455	0.228348	4.276166	1.90E-05	0.0015352
AAEL012099	1309.418	1.021207	0.240332	4.249157	2.15E-05	0.0017107
AAEL009387	9606.411	0.619584	0.145967	4.244688	2.19E-05	0.0017342
AAEL001243	89.52798	1.073912	0.253295	4.239776	2.24E-05	0.0017507
AAEL005342	799.5598	0.821281	0.193663	4.24078	2.23E-05	0.0017507
AAEL025894	12.7806	1.802038	0.425469	4.235418	2.28E-05	0.0017738
AAEL018241	342.8322	1.444882	0.341251	4.234077	2.29E-05	0.0017738
AAEL024838	514.8578	0.87682	0.207826	4.219001	2.45E-05	0.0018852
AAEL001548	574.2691	1.084587	0.257205	4.21681	2.48E-05	0.0018921
AAEL001965	1088.742	1.402411	0.332704	4.215188	2.50E-05	0.0018943
AAEL006069	504.3606	1.30271	0.309209	4.213035	2.52E-05	0.0019011
AAEL017144	1836.946	1.156814	0.274713	4.21099	2.54E-05	0.0019071
AAEL002959	175.2784	1.549768	0.368929	4.200728	2.66E-05	0.0019723
AAEL008096	512.3748	1.291174	0.310802	4.154332	3.26E-05	0.0023767
AAEL006815	256.5448	1.087856	0.262123	4.150172	3.32E-05	0.0024065
AAEL014510	244.9046	1.110525	0.268041	4.14312	3.43E-05	0.0024676

GeneID	Base mean	log2(FC)	StdErr	Wald-Stats	P-value	P-adj
AAEL007206	1011.047	1.095975	0.265351	4.130285	3.62E-05	0.0025947
AAEL020603	54.94935	1.18837	0.288531	4.118684	3.81E-05	0.0027135
AAEL014349	207.4646	1.071446	0.26027	4.116675	3.84E-05	0.002722
AAEL006361	16.66943	1.408174	0.344199	4.091161	4.29E-05	0.003006
AAEL009660	94.57614	1.152919	0.282251	4.084728	4.41E-05	0.0030735
AAEL027188	39.3365	1.510878	0.370816	4.07447	4.61E-05	0.0031946
AAEL001307	98.75584	1.499311	0.368137	4.072695	4.65E-05	0.0032015
AAEL003640	370.2143	1.046696	0.257521	4.064503	4.81E-05	0.0032982
AAEL007902	172.9648	1.16835	0.287866	4.058662	4.94E-05	0.0033636
AAEL026843	7790.01	1.160491	0.286056	4.056872	4.97E-05	0.0033713
AAEL019528	1488.322	0.892322	0.220601	4.044963	5.23E-05	0.0035098
AAEL009531	203.9522	1.610651	0.399908	4.027552	5.64E-05	0.003721
AAEL007765	4599.867	0.990208	0.246687	4.014026	5.97E-05	0.0039205
AAEL011867	103.0433	1.145468	0.285495	4.012217	6.02E-05	0.0039303
AAEL029031	190.9685	1.168092	0.291701	4.004412	6.22E-05	0.0040415
AAEL005666	566.7667	1.052103	0.26297	4.000854	6.31E-05	0.0040818
AAEL027093	3.850387	1.668462	0.41737	3.997558	6.40E-05	0.0040894
AAEL003632	31.74144	1.532191	0.383149	3.998939	6.36E-05	0.0040894
AAEL006754	177.584	1.065677	0.26671	3.995634	6.45E-05	0.0040894
AAEL003888	1937.104	1.435117	0.359065	3.996812	6.42E-05	0.0040894
AAEL013812	571.2525	1.594401	0.399522	3.990774	6.59E-05	0.0041328
AAEL026447	4.585397	1.67971	0.421203	3.987887	6.67E-05	0.0041627
AAEL017023	760.2604	0.825842	0.207597	3.978098	6.95E-05	0.0042955
AAEL006280	45.33233	1.287139	0.32376	3.975591	7.02E-05	0.0043055
AAEL009630	257.3899	1.001203	0.25186	3.975235	7.03E-05	0.0043055
AAEL010625	8.018426	1.642687	0.41373	3.970431	7.17E-05	0.0043512
AAEL013341	1207.901	1.21579	0.306183	3.970794	7.16E-05	0.0043512
AAEL019578	203.8957	1.193456	0.300841	3.967064	7.28E-05	0.0043921
AAEL010479	1728.988	0.988333	0.249786	3.956721	7.60E-05	0.0045649
AAEL014618	34.6267	1.686867	0.426507	3.955074	7.65E-05	0.0045748
AAEL007792	278.9527	1.207479	0.306052	3.945344	7.97E-05	0.0047201
AAEL008354	111.4486	1.057035	0.267885	3.945848	7.95E-05	0.0047201
AAEL001373	33.94338	1.283716	0.32588	3.939227	8.17E-05	0.0047972
AAEL014076	41.77342	1.653518	0.419674	3.940003	8.15E-05	0.0047972
AAEL003737	172.2251	1.090585	0.277245	3.933651	8.37E-05	0.0048873
AAEL007703	743.8899	1.651893	0.420655	3.926951	8.60E-05	0.0050023
AAEL008699	77.33347	1.672026	0.426238	3.922755	8.75E-05	0.005067
AAEL024560	92.78694	1.200297	0.308026	3.896736	9.75E-05	0.0056177
AAEL018351	517.4097	0.966635	0.24819	3.894738	9.83E-05	0.0056385
AAEL019868	142.1417	1.306783	0.336771	3.880331	0.0001043	0.0059563
AAEL026300	36.49657	1.620907	0.418581	3.872383	0.0001078	0.0061263
AAEL006394	10.63294	1.387161	0.358467	3.8697	0.000109	0.0061665
AAEL022674	138.1285	1.458834	0.378489	3.85436	0.000116	0.006537
AAEL003051	92.12374	1.288741	0.334764	3.849699	0.0001183	0.0066332
AAEL008628	17.79751	1.411862	0.368837	3.827872	0.0001293	0.0071862

GeneID	Base mean	log2(FC)	StdErr	Wald-Stats	P-value	P-adj
AAEL003857	72.99003	1.604194	0.419271	3.82615	0.0001302	0.007205
AAEL027699	13.28489	1.386421	0.363289	3.816303	0.0001355	0.0073558
AAEL007090	256.6072	1.298519	0.340496	3.813614	0.0001369	0.0073558
AAEL024038	138.8779	1.010885	0.265026	3.814294	0.0001366	0.0073558
AAEL023882	10.13949	1.549059	0.405857	3.816764	0.0001352	0.0073558
AAEL011980	130.3171	1.120688	0.294684	3.803022	0.0001429	0.0075912
AAEL027632	623.31	1.436387	0.377728	3.802699	0.0001431	0.0075912
AAEL020340	7667.399	0.903366	0.238488	3.787893	0.0001519	0.0080244
AAEL002175	2093.977	0.587213	0.155435	3.777878	0.0001582	0.0083194
AAEL025750	156.3401	1.21716	0.323445	3.763119	0.0001678	0.0086822
AAEL003527	60.39437	1.041641	0.276793	3.763242	0.0001677	0.0086822
AAEL013257	82.30367	1.165331	0.309554	3.764553	0.0001668	0.0086822
AAEL025839	30.1891	1.555673	0.413083	3.766007	0.0001659	0.0086822
AAEL005491	235.1988	1.077765	0.28707	3.754358	0.0001738	0.0088827
AAEL001519	144.9248	1.34742	0.358811	3.755233	0.0001732	0.0088827
AAEL002254	591.4642	0.876364	0.233521	3.752835	0.0001748	0.008901
AAEL014773	7.563296	1.387879	0.370479	3.746172	0.0001796	0.0090817
AAEL003389	37.29007	1.578976	0.421534	3.745788	0.0001798	0.0090817
AAEL029046	13.03295	1.559203	0.416459	3.743951	0.0001811	0.0091121
AAEL025552	100.2473	1.224406	0.327524	3.738373	0.0001852	0.0092433
AAEL012859	101.3332	1.388411	0.371717	3.735133	0.0001876	0.0093264
AAEL009126	41.73404	1.589443	0.426507	3.726651	0.000194	0.0095707
AAEL012856	399.5885	1.165543	0.313075	3.72289	0.000197	0.0096768
AAEL009487	3417.461	1.014725	0.27331	3.712721	0.000205	0.0099583
AAEL001417	229.3783	1.326564	0.357297	3.712779	0.000205	0.0099583
AAEL028221	66.42982	1.059166	0.285634	3.708123	0.0002088	0.0100637
AAEL012184	150.9025	0.996729	0.269675	3.696034	0.000219	0.0104754
AAEL014316	53.42481	1.162642	0.314961	3.691385	0.000223	0.0106286
AAEL013600	815.7912	0.75663	0.205186	3.687537	0.0002264	0.01071
AAEL020033	453.895	1.239684	0.336099	3.688453	0.0002256	0.01071
AAEL015644	237.9285	1.10361	0.299777	3.681441	0.0002319	0.0108882
AAEL007322	1890.898	0.954807	0.259324	3.681912	0.0002315	0.0108882
AAEL006114	7.660662	1.513053	0.412564	3.667436	0.000245	0.0114596
AAEL014539	48.80451	1.38572	0.37831	3.662926	0.0002494	0.0115779
AAEL021583	474.0557	1.154128	0.316011	3.65218	0.00026	0.0120294
AAEL025392	10.03591	1.496365	0.41126	3.638491	0.0002742	0.0125497
AAEL026537	13.80957	1.257432	0.346143	3.632699	0.0002805	0.0127887
AAEL006126	93.96053	1.502308	0.413837	3.630191	0.0002832	0.0128673
AAEL020078	15.49207	1.341617	0.369746	3.628487	0.0002851	0.0129063
AAEL012091	149.8871	0.844295	0.233129	3.621572	0.0002928	0.0131623
AAEL026440	1936.124	1.064952	0.294319	3.618365	0.0002965	0.0132794
AAEL009837	408.4249	1.014612	0.280807	3.613194	0.0003024	0.0134994
AAEL005130	66.79305	0.914671	0.253274	3.61139	0.0003046	0.013546
AAEL014343	22.30363	1.267359	0.352035	3.600088	0.0003181	0.01405
AAEL025125	5.258394	1.532462	0.426213	3.595532	0.0003237	0.0142486

GeneID	Base mean	log2(FC)	StdErr	Wald-Stats	P-value	P-adj
AAEL009249	296.5556	1.05174	0.294025	3.577048	0.0003475	0.0150343
AAEL002964	579.7919	0.95955	0.268206	3.577665	0.0003467	0.0150343
AAEL003420	140.9826	1.049211	0.293817	3.570966	0.0003557	0.0153348
AAEL024468	7.567725	1.517422	0.425983	3.562164	0.0003678	0.0158047
AAEL001536	30.53871	1.240871	0.348656	3.559012	0.0003723	0.0159415
AAEL013875	5029.768	0.899848	0.252949	3.557425	0.0003745	0.015984
AAEL009148	332.5632	1.042286	0.293367	3.55284	0.0003811	0.0161565
AAEL027655	13.87487	1.330922	0.374997	3.549156	0.0003865	0.0163296
AAEL005768	447.8671	1.361932	0.384271	3.544191	0.0003938	0.0165849
AAEL007403	236.0034	0.961803	0.272173	3.533787	0.0004097	0.0171377
AAEL001818	387.54	1.117286	0.31656	3.529456	0.0004164	0.0173634
AAEL021016	200.1772	0.772463	0.219288	3.522597	0.0004273	0.0177605
AAEL024540	137.1792	0.991282	0.281532	3.521032	0.0004299	0.0178073
AAEL007502	320.1961	0.846843	0.241569	3.505591	0.0004556	0.0188114
AAEL010881	80.64011	1.212936	0.346136	3.504221	0.0004579	0.0188471
AAEL010451	73.26203	1.116715	0.319472	3.495501	0.0004732	0.0193482
AAEL001928	1682.96	1.018857	0.291817	3.491427	0.0004804	0.0195825
AAEL021982	17.62089	1.166193	0.334432	3.487086	0.0004883	0.019776
AAEL008489	242.942	0.967137	0.27772	3.48242	0.0004969	0.0200555
AAEL002390	851.7374	1.068684	0.30695	3.481624	0.0004984	0.0200555
AAEL024669	730.7402	1.029516	0.2965	3.472226	0.0005162	0.0207053
AAEL001693	176.3984	1.406068	0.405565	3.466939	0.0005264	0.0210502
AAEL014910	30.89665	1.34186	0.388051	3.457945	0.0005443	0.0216292
AAEL006138	142.0151	1.469892	0.425706	3.452833	0.0005547	0.0219059
AAEL023243	28.10678	1.117499	0.324289	3.446003	0.0005689	0.0223973
AAEL022600	400.9745	1.363779	0.396013	3.443773	0.0005737	0.0225082
AAEL014138	164.986	0.802264	0.233013	3.442994	0.0005753	0.0225082
AAEL012412	22.49855	1.275602	0.370778	3.44034	0.000581	0.0225906
AAEL004719	340.5183	0.879947	0.255738	3.440813	0.00058	0.0225906
AAEL002908	49.15145	1.465278	0.426499	3.435598	0.0005912	0.0229195
AAEL008404	19.86394	1.411783	0.412881	3.419349	0.0006277	0.0242588
AAEL009869	1228.05	0.608743	0.178301	3.414127	0.0006399	0.0243841
AAEL019844	58.7366	0.90623	0.265459	3.413827	0.0006406	0.0243841
AAEL019537	442.6477	1.402509	0.410702	3.414902	0.0006381	0.0243841
AAEL011779	149.1592	0.870939	0.255109	3.413989	0.0006402	0.0243841
AAEL013525	597.8701	0.888488	0.260494	3.410784	0.0006478	0.024584
AAEL012093	571.972	1.056681	0.310395	3.40431	0.0006633	0.025099
AAEL013345	2091.104	1.351629	0.397873	3.397132	0.000681	0.025538
AAEL011967	912.1809	0.877246	0.258229	3.397158	0.0006809	0.025538
AAEL024112	607.4509	1.092194	0.321654	3.395553	0.0006849	0.0256101
AAEL019893	337.8388	0.60392	0.17792	3.394337	0.000688	0.0256484
AAEL005849	8412.767	1.155223	0.340574	3.391991	0.0006939	0.0257931
AAEL012549	1065.228	0.976689	0.288427	3.386262	0.0007085	0.0262175
AAEL007693	311.1743	1.157429	0.341837	3.385913	0.0007094	0.0262175
AAEL003345	10284.03	1.172598	0.34724	3.376911	0.000733	0.0269337

GeneID	Base mean	log2(FC)	StdErr	Wald-Stats	P-value	P-adj
AAEL027362	31.42138	1.391915	0.41233	3.375731	0.0007362	0.0269712
AAEL027829	171.7545	1.338293	0.396624	3.374211	0.0007403	0.0270425
AAEL026041	13.17522	1.33437	0.395992	3.369688	0.0007525	0.0273327
AAEL008832	2258.953	0.698426	0.207252	3.369935	0.0007519	0.0273327
AAEL000242	45.51674	1.024009	0.304451	3.363464	0.0007697	0.0277182
AAEL008278	412.195	1.326029	0.394747	3.359186	0.0007817	0.0280712
AAEL013906	19.18083	1.356959	0.404346	3.355934	0.000791	0.0283232
AAEL022829	9.194961	1.414172	0.422182	3.349674	0.0008091	0.0288894
AAEL025079	8.309693	1.407754	0.420599	3.347024	0.0008168	0.028928
AAEL009081	1992.548	0.644463	0.192496	3.347939	0.0008142	0.028928
AAEL004805	2452.177	1.189846	0.356103	3.341295	0.0008339	0.029362
AAEL002130	2748.739	0.739045	0.221291	3.339691	0.0008387	0.0294504
AAEL004572	127.7069	1.028086	0.30798	3.338163	0.0008433	0.029531
AAEL006329	17.39274	1.202431	0.361327	3.327817	0.0008753	0.0303979
AAEL027493	152.3785	0.754039	0.226649	3.326902	0.0008782	0.0304145
AAEL012450	121.4309	0.868228	0.262584	3.306471	0.0009448	0.0323681
AAEL005651	6837.951	0.707463	0.213943	3.306783	0.0009437	0.0323681
AAEL007191	411.6934	1.08428	0.32835	3.302211	0.0009593	0.0327751
AAEL013163	932.7747	1.002523	0.304206	3.29554	0.0009823	0.0333833
AAEL019773	78.99006	1.398552	0.424672	3.29325	0.0009904	0.0335665
AAEL000618	34.66495	1.210145	0.368289	3.285855	0.0010167	0.0341536
AAEL000037	418.4091	1.093827	0.332938	3.285376	0.0010185	0.0341536
AAEL009888	481.8975	0.805992	0.245405	3.284341	0.0010222	0.0341888
AAEL024175	55.25191	0.911478	0.277895	3.279933	0.0010383	0.0345452
AAEL002661	10.03927	1.396822	0.426485	3.275194	0.0010559	0.035038
AAEL006352	247.1212	1.071658	0.327645	3.270791	0.0010725	0.0354951
AAEL001349	60.11651	0.855291	0.262136	3.262776	0.0011033	0.0361209
AAEL004213	55.24829	1.285933	0.394022	3.263609	0.0011	0.0361209
AAEL008668	44.47421	0.940649	0.288628	3.259041	0.0011179	0.0364279
AAEL008336	26.22536	0.957256	0.293909	3.256985	0.001126	0.036505
AAEL019438	372.2857	1.24651	0.382814	3.25618	0.0011292	0.0365154
AAEL022427	44.84072	1.180727	0.36314	3.25144	0.0011482	0.0370352
AAEL000164	106.7757	1.220664	0.375644	3.24952	0.001156	0.0371915
AAEL010738	1982.918	0.822461	0.253202	3.248236	0.0011612	0.0372651
AAEL027514	412.0888	0.701038	0.216342	3.240417	0.0011936	0.0381095
AAEL007535	268.9715	0.807575	0.24955	3.236131	0.0012116	0.0385891
AAEL000102	1418.612	0.99988	0.309177	3.234008	0.0012207	0.0387556
AAEL001646	154.4133	1.290689	0.399209	3.233116	0.0012245	0.0387556
AAEL000967	611.8377	0.792888	0.245267	3.232757	0.001226	0.0387556
AAEL010067	14.60293	1.075311	0.332704	3.23203	0.0012291	0.0387577
AAEL015416	9.73182	1.291735	0.400928	3.221862	0.0012736	0.0399963
AAEL003738	117.2808	1.200916	0.372971	3.219863	0.0012825	0.0400424
AAEL010413	4.499809	1.318839	0.40987	3.217696	0.0012922	0.0400494
AAEL012341	568.3146	0.97098	0.301644	3.218965	0.0012865	0.0400494
AAEL019849	447.027	0.79886	0.248324	3.217001	0.0012954	0.0400494

GeneID	Base mean	log2(FC)	StdErr	Wald-Stats	P-value	P-adj
AAEL007143	48.75739	1.058717	0.329087	3.217133	0.0012948	0.0400494
AAEL009464	40.25879	1.103822	0.343521	3.213257	0.0013124	0.0403527
AAEL006582	31869.16	0.690617	0.215008	3.212048	0.0013179	0.0403527
AAEL001062	277.9282	0.948194	0.295525	3.208506	0.0013343	0.0407546
AAEL015458	380.1295	1.09973	0.343444	3.202062	0.0013645	0.0415667
AAEL002347	155.4017	1.063669	0.332247	3.201443	0.0013674	0.0415667
AAEL023560	68.28717	0.970427	0.303694	3.195409	0.0013963	0.0421425
AAEL000834	559.4591	1.144341	0.358232	3.194416	0.0014011	0.0421873
AAEL022876	8.691477	1.173543	0.367778	3.190904	0.0014183	0.0425015
AAEL009038	518.4585	1.011739	0.317871	3.182855	0.0014583	0.0434953
AAEL027106	190.1763	1.027908	0.323191	3.180491	0.0014703	0.0437487
AAEL004533	129.7041	0.914981	0.288245	3.174316	0.0015019	0.0443425
AAEL003703	19.06008	1.269577	0.400009	3.173872	0.0015042	0.0443425
AAEL000428	1438.551	1.014774	0.319595	3.175189	0.0014974	0.0443425
AAEL006113	677.8402	0.730493	0.230422	3.170244	0.0015231	0.0447956
AAEL025332	10.69345	1.283329	0.40543	3.165353	0.001549	0.0454472
AAEL008274	1215.456	0.888387	0.280718	3.164699	0.0015524	0.0454472
AAEL005676	86.96242	1.143142	0.36143	3.162828	0.0015624	0.0456349
AAEL003229	2207.826	0.59259	0.187414	3.161923	0.0015673	0.0456718
AAEL011180	3243.92	1.028451	0.325333	3.161228	0.0015711	0.0456759
AAEL019767	806.3366	0.835747	0.264885	3.155132	0.0016043	0.0465343
AAEL001094	955.4217	0.693881	0.220024	3.153662	0.0016124	0.0466627
AAEL005177	419.3467	0.887037	0.282033	3.145156	0.00166	0.0479315
AAEL006904	1363.188	0.58096	0.184792	3.143852	0.0016674	0.0480363
AAEL012135	286.5131	0.923976	0.294007	3.142698	0.001674	0.0481167
AAEL027270	10.83758	1.293931	0.412253	3.13868	0.0016971	0.048561
AAEL019463	415.8936	0.801131	0.255357	3.137294	0.0017052	0.0486813
AAEL007993	21.5142	1.25305	0.399686	3.135083	0.0017181	0.0488298
AAEL005017	1179.668	0.716334	0.228478	3.135243	0.0017171	0.0488298
AAEL010379	496.8672	0.802462	0.256622	3.127014	0.0017659	0.049966
AAEL001318	17.67618	1.063269	0.339988	3.127375	0.0017637	0.049966

FC: fold change; P-adj: adjusted *p* value

3 dpi 28 °C downregulated

GeneID	Base mean	log2(FC)	StdErr	Wald-Stats	P-value	P-adj
AAEL012628	474.5021	-2.8039	0.406212	-6.90257	5.11E-12	1.51E-09
AAEL000507	59.32845	-1.97832	0.357445	-5.53463	3.12E-08	5.00E-06
AAEL009899	1587.204	-1.52068	0.309149	-4.91894	8.70E-07	0.0001114
AAEL010891	88.07134	-1.21154	0.24657	-4.91356	8.94E-07	0.0001134
AAEL018189	59.77118	-1.71655	0.39002	-4.40117	1.08E-05	0.0009962
AAEL025401	592.1465	-0.86644	0.198485	-4.36527	1.27E-05	0.0011255
AAEL028022	94.78526	-0.8702	0.204456	-4.25616	2.08E-05	0.0016685
AAEL022506	603.8726	-0.90026	0.214022	-4.20637	2.60E-05	0.001935
AAEL010855	777.8795	-1.40369	0.337708	-4.15651	3.23E-05	0.0023677

GeneID	Base mean	log2(FC)	StdErr	Wald-Stats	P-value	P-adj
AAEL002785	647.5887	-0.71852	0.177452	-4.04913	5.14E-05	0.0034663
AAEL020175	24.92202	-1.70906	0.4231	-4.03937	5.36E-05	0.0035756
AAEL003916	2524.846	-0.65688	0.163013	-4.0296	5.59E-05	0.003708
AAEL005507	193.9252	-1.70281	0.4265	-3.99251	6.54E-05	0.0041229
AAEL011983	1222.265	-0.83232	0.208822	-3.98577	6.73E-05	0.0041794
AAEL006313	294.6973	-1.11518	0.291314	-3.82809	0.0001291	0.0071862
AAEL024022	1257.093	-0.66379	0.173974	-3.81545	0.0001359	0.0073558
AAEL014295	769.078	-0.92575	0.243127	-3.80767	0.0001403	0.0075029
AAEL011710	642.353	-0.95844	0.255195	-3.75572	0.0001728	0.0088827
AAEL011911	565.861	-0.62327	0.166709	-3.73866	0.000185	0.0092433
AAEL005577	278.9741	-0.76512	0.205044	-3.73146	0.0001904	0.0094264
AAEL014263	66.07671	-1.15402	0.310698	-3.71428	0.0002038	0.0099583
AAEL011510	132.9247	-1.06661	0.28816	-3.70144	0.0002144	0.0102933
AAEL012455	376.7221	-0.763	0.208191	-3.66488	0.0002475	0.011532
AAEL006614	282.8583	-0.73253	0.202205	-3.62268	0.0002916	0.0131526
AAEL019677	1164.556	-0.90249	0.253846	-3.55526	0.0003776	0.0160621
AAEL012566	70.42868	-1.46172	0.413295	-3.53675	0.0004051	0.017003
AAEL026775	414.3637	-0.83955	0.249591	-3.36371	0.000769	0.0277182
AAEL026260	85.39223	-1.1222	0.333522	-3.36468	0.0007663	0.0277182
AAEL022382	36.42032	-1.29198	0.387719	-3.33226	0.0008615	0.0300415
AAEL026241	507.6766	-0.59299	0.178602	-3.32017	0.0008996	0.0310731
AAEL022900	50.1307	-1.36645	0.414138	-3.29951	0.0009685	0.033003
AAEL024298	83.20629	-1.1401	0.34678	-3.28769	0.0010101	0.0341457
AAEL013816	1131.992	-0.59861	0.182456	-3.28084	0.001035	0.0345246
AAEL021303	127.5461	-1.29047	0.39539	-3.26379	0.0010993	0.0361209
AAEL006488	1316.479	-0.58701	0.182219	-3.22148	0.0012753	0.0399963
AAEL025549	22.5495	-0.80927	0.253398	-3.19369	0.0014047	0.0421933

FC: fold change; P-adj: adjusted *p* value

3 dpi 32 ºC upregulated

GeneID	Base mean	log2(FC)	StdErr	Wald-stats	P-value	P-adj
AAEL013339	87.53917	2.487519	0.370644	6.711338	1.93E-11	2.37E-07
AAEL004090	1851.692	1.374891	0.247752	5.549469	2.87E-08	0.000176
AAEL013349	673.0459	2.055251	0.377009	5.451465	5.00E-08	0.000205
AAEL013346	755.9516	2.024833	0.376885	5.372545	7.76E-08	0.000239
AAEL017976	6791.25	1.939832	0.374036	5.186217	2.15E-07	0.000442
AAEL013345	1284.808	1.955582	0.377127	5.185477	2.15E-07	0.000442
AAEL013348	219.7709	1.89139	0.3689	5.127108	2.94E-07	5.17E-04
AAEL013351	775.3334	1.630181	0.321914	5.064033	4.10E-07	6.31E-04
AAEL020330	1528.054	1.838949	0.37786	4.866747	1.13E-06	0.001551
AAEL013350	4300.273	1.82661	0.378096	4.831071	1.36E-06	0.00167
AAEL006883	1071.751	1.315066	0.311461	4.222249	2.42E-05	0.021401
AAEL006886	129.0013	1.504414	0.354119	4.248332	2.15E-05	0.021401
AAEL013257	86.11675	1.150659	0.273634	4.205105	2.61E-05	0.021401

GeneID	Base mean	log2(FC)	StdErr	Wald-Stats	P-value	P-adj
AAEL021012	439.7245	1.43843	0.338622	4.247893	2.16E-05	0.021401
AAEL010068	1739.129	1.141382	0.27103	4.211268	2.54E-05	0.021401
AAEL009682	7.136665	1.547103	0.369836	4.183215	2.87E-05	0.022097
AAEL010793	302.8245	1.010064	0.244752	4.126884	3.68E-05	0.023585
AAEL012184	144.1262	0.885474	0.214867	4.121029	3.77E-05	0.023585
AAEL009660	114.36	0.905478	0.219925	4.11722	3.83E-05	0.023585
AAEL023321	363.3567	1.400596	0.342886	4.084733	4.41E-05	0.025848
AAEL006126	191.997	1.229758	0.30412	4.043663	5.26E-05	0.028144
AAEL012351	162.5252	1.471971	0.363539	4.049009	5.14E-05	0.028144
AAEL024560	144.7041	1.161194	0.291786	3.979602	6.90E-05	0.035288
AAEL004478	15.70645	1.311482	0.330305	3.970516	7.17E-05	0.035288
AAEL002741	36.46224	1.158966	0.297029	3.901856	9.55E-05	0.043942
AAEL005977	533.8883	0.761845	0.196042	3.886133	0.000102	0.044682

FC: fold change; P-adj: adjusted *p* value

3 dpi 32 °C downregulated

GeneID	Base mean	log2(FC)	StdErr	Wald-Stats	P-value	P-adj
AAEL024207	101.1526	-1.57107	0.377418	-4.16268	3.15E-05	0.022759
AAEL000668	67.81704	-1.40938	0.361439	-3.89935	9.64E-05	0.043942
AAEL014019	197.0831	-1.43832	0.370897	-3.87796	0.000105	0.044682
AAEL011126	406.0354	-1.27551	0.331352	-3.84943	0.000118	0.048546

FC: fold change; P-adj: adjusted *p* value

7 dpi 18 °C upregulated

GeneID	Base mean	log2(FC)	StdErr	Wald-Stats	P-value	P-adj
AAEL013346	420.3165	4.176572	0.302726	13.79656	2.67E-43	3.46E-39
AAEL013348	150.1522	3.462339	0.280088	12.36163	4.22E-35	2.72E-31
AAEL013350	2381.49	3.89112	0.32236	12.07075	1.51E-33	6.50E-30
AAEL013339	63.31234	3.71537	0.317903	11.68712	1.48E-31	4.79E-28
AAEL013351	631.1653	3.197844	0.296957	10.76872	4.84E-27	1.25E-23
AAEL017975	8101.265	3.147815	0.327586	9.609137	7.32E-22	1.58E-18
AAEL017976	4130.422	3.142821	0.332892	9.440963	3.69E-21	6.82E-18
AAEL013347	800.9459	1.953186	0.217144	8.994871	2.37E-19	3.82E-16
AAEL022079	396.6688	2.314959	0.279665	8.277602	1.26E-16	1.81E-13
AAEL019751	955.3816	1.67476	0.212444	7.883309	3.19E-15	4.12E-12
AAEL013349	274.9653	2.490805	0.328152	7.590404	3.19E-14	3.75E-11
AAEL006352	298.3305	1.251859	0.172568	7.254283	4.04E-13	4.35E-10
AAEL022253	4523.436	2.439641	0.337721	7.223847	5.05E-13	5.03E-10
AAEL024512	162.5634	2.388031	0.334364	7.142006	9.20E-13	8.49E-10
AAEL022059	2395.029	2.202666	0.33608	6.553999	5.60E-11	4.83E-08
AAEL023321	151.8003	2.029257	0.323287	6.276943	3.45E-10	2.79E-07
AAEL023395	121.0724	2.045191	0.328381	6.228096	4.72E-10	3.59E-07
AAEL027610	2684.146	2.056135	0.337229	6.097151	1.08E-09	7.76E-07

GeneID	Base mean	log2(FC)	StdErr	Wald-Stats	P-value	P-adj
AAEL001857	134.3301	1.906687	0.336916	5.659238	1.52E-08	1.03E-05
AAEL006883	927.2206	1.564359	0.287865	5.434357	5.50E-08	3.55E-05
AAEL003737	149.6336	1.054883	0.197164	5.350283	8.78E-08	5.41E-05
AAEL026833	557.9738	1.531724	0.290752	5.26815	1.38E-07	8.10E-05
AAEL009881	354.4552	0.934855	0.181892	5.139604	2.75E-07	0.000155
AAEL024597	267.3648	0.828234	0.163736	5.058341	4.23E-07	0.000228
AAEL002209	189.6914	1.11988	0.223811	5.003687	5.62E-07	0.000291
AAEL026008	1605.445	1.410719	0.28551	4.941043	7.77E-07	0.000386
AAEL021072	40.69005	1.404413	0.289463	4.851787	1.22E-06	0.000565
AAEL013077	1172.399	1.259947	0.260178	4.842638	1.28E-06	0.000571
AAEL002075	33.58957	1.425952	0.303065	4.7051	2.54E-06	0.001093
AAEL013770	92.40521	1.069836	0.228253	4.687066	2.77E-06	0.001156
AAEL003505	2530.577	1.361659	0.29527	4.611572	4.00E-06	0.001603
AAEL006276	238.8906	1.189589	0.25823	4.606706	4.09E-06	0.001603
AAEL003726	44.47114	1.516708	0.329791	4.598992	4.25E-06	0.00161
AAEL000915	310.5454	1.320343	0.294409	4.484733	7.30E-06	0.002484
AAEL025894	23.83281	1.477798	0.33409	4.423353	9.72E-06	0.003064
AAEL006902	726.9372	1.383176	0.314324	4.400477	1.08E-05	0.003314
AAEL003886	54.19795	1.174559	0.267184	4.396067	1.10E-05	0.003314
AAEL007206	983.3226	1.088468	0.249164	4.368476	1.25E-05	0.003676
AAEL005772	485.0575	1.387753	0.319198	4.347618	1.38E-05	0.003954
AAEL024038	206.2591	0.944778	0.218621	4.321531	1.55E-05	0.004355
AAEL020092	18951.2	0.941047	0.221991	4.239122	2.24E-05	0.006172
AAEL008622	57.86806	1.386567	0.333706	4.155058	3.25E-05	0.00858
AAEL004919	229.0628	0.94733	0.231526	4.091688	4.28E-05	0.011073
AAEL008953	11806.12	0.820875	0.202235	4.059016	4.93E-05	0.011912
AAEL003728	141.0934	1.194262	0.294594	4.053923	5.04E-05	0.011912
AAEL010262	1913.182	0.99813	0.246301	4.052482	5.07E-05	0.011912
AAEL002661	7.098293	1.367789	0.337293	4.055196	5.01E-05	0.011912
AAEL012352	1303.102	0.888156	0.218583	4.063244	4.84E-05	0.011912
AAEL007200	4.079949	1.34003	0.332504	4.030112	5.58E-05	0.01287
AAEL023591	2404.098	0.875281	0.219422	3.989024	6.63E-05	0.015048
AAEL028247	55.4548	1.340884	0.336799	3.981262	6.86E-05	0.01528
AAEL026031	105.5293	0.868658	0.218818	3.969771	7.19E-05	0.015764
AAEL000334	62.87674	1.303493	0.331749	3.929158	8.52E-05	0.018367
AAEL027802	75.8778	1.0622	0.273047	3.890171	0.0001	0.020888
AAEL024257	9.453414	1.296162	0.337273	3.843067	0.000122	0.023637
AAEL013352	267.5548	1.291916	0.336256	3.842063	0.000122	0.023637
AAEL000445	312.4624	0.871034	0.226984	3.837427	0.000124	0.023637
AAEL013341	1175.571	0.920469	0.241839	3.806125	0.000141	0.025703
AAEL002130	3246.238	0.881223	0.231354	3.808978	0.00014	0.025703
AAEL003883	1403.115	0.944435	0.248609	3.798873	0.000145	0.026099
AAEL024887	410.0083	0.825397	0.218224	3.782349	0.000155	0.026278
AAEL010712	1792.319	0.910695	0.241301	3.774108	0.000161	0.026278
AAEL010120	333.82	0.855971	0.225969	3.787994	0.000152	0.026278

GeneID	Base mean	log2(FC)	StdErr	Wald-Stats	P-value	P-adj
AAEL010242	512.101	1.274405	0.337638	3.774468	0.00016	0.026278
AAEL001928	2105.611	0.953398	0.252352	3.778043	0.000158	0.026278
AAEL000545	286.5021	1.215807	0.320857	3.789245	0.000151	0.026278
AAEL005829	244.4502	0.81641	0.21755	3.752741	0.000175	0.026813
AAEL020330	770.7974	1.202723	0.320253	3.755543	0.000173	0.026813
AAEL025731	16.17947	1.225227	0.325725	3.761532	0.000169	0.026813
AAEL019688	34.81125	1.268243	0.337741	3.755078	0.000173	0.026813
AAEL019704	304.8896	0.876778	0.233212	3.759574	0.00017	0.026813
AAEL000780	222.1955	0.805576	0.214776	3.75077	0.000176	0.026813
AAEL020804	278.0058	0.618901	0.165558	3.738272	0.000185	0.027221
AAEL007225	22.29841	1.200419	0.320995	3.739683	0.000184	0.027221
AAEL023002	15.16646	1.262493	0.337715	3.738335	0.000185	0.027221
AAEL010260	1553.447	0.798033	0.213993	3.729244	0.000192	0.027898
AAEL019684	35.98889	1.114147	0.299567	3.719188	0.0002	0.028709
AAEL013345	672.0795	1.143719	0.308117	3.711969	0.000206	0.028899
AAEL003966	237.7414	0.767735	0.206747	3.713401	0.000204	0.028899
AAEL003888	2273.902	1.087065	0.294347	3.693142	0.000222	0.030463
AAEL011371	3573.692	0.750477	0.203999	3.678828	0.000234	0.031228
AAEL020028	179.8287	0.911619	0.24755	3.682565	0.000231	0.031228
AAEL005967	32.7544	1.172761	0.319496	3.670662	0.000242	0.031592
AAEL010680	65.99109	1.230371	0.336492	3.656461	0.000256	0.032732
AAEL004023	308.9545	0.714887	0.196669	3.634984	0.000278	0.034489
AAEL002853	589.2938	0.863334	0.237795	3.630584	0.000283	0.034489
AAEL019899	7.83657	1.223632	0.336482	3.636547	0.000276	0.034489
AAEL011520	701.7817	0.784751	0.216038	3.632467	0.000281	0.034489
AAEL023555	37.80518	1.094452	0.302312	3.620269	0.000294	0.035558
AAEL022829	12.63532	1.18367	0.327399	3.615373	0.0003	0.035572
AAEL025332	16.18794	1.087082	0.300661	3.615639	0.0003	0.035572
AAEL029030	11.87014	1.213811	0.336172	3.610691	0.000305	0.035891
AAEL002124	879.8008	0.680503	0.189022	3.600127	0.000318	0.036813
AAEL009541	411.2336	0.763981	0.212251	3.599425	0.000319	0.036813
AAEL013190	5.974054	1.197853	0.334176	3.584491	0.000338	0.038639
AAEL006844	18.22367	1.172727	0.328817	3.566498	0.000362	0.041027
AAEL006132	35.57626	1.048723	0.294406	3.562165	0.000368	0.041348
AAEL010881	138.9453	1.169439	0.329736	3.546592	0.00039	0.043493
AAEL026848	42.01983	1.190459	0.337019	3.53232	0.000412	0.045517
AAEL005961	9323.726	0.925108	0.262746	3.520927	0.00043	0.047115

FC: fold change; P-adj: adjusted *p* value

7 dpi 18 °C downregulated

GeneID	Base mean	log2(FC)	StdErr	Wald-Stats	P-value	P-adj
AAEL023617	399.222	-0.71837	0.147716	-4.86317	1.16E-06	0.000553
AAEL012219	1561.792	-0.63644	0.138551	-4.59351	4.36E-06	0.00161
AAEL029039	21.97969	-1.27506	0.278798	-4.57342	4.80E-06	0.001723
AAEL006151	215.0111	-1.50159	0.337762	-4.4457	8.76E-06	0.002856
AAEL009567	119.6869	-1.48522	0.33422	-4.44386	8.84E-06	0.002856
AAEL008789	4835.027	-1.25408	0.2987	-4.19847	2.69E-05	0.007238
AAEL010620	42.17635	-1.28654	0.330677	-3.89062	1.00E-04	0.020888
AAEL011263	93.60525	-0.89784	0.23138	-3.88035	0.000104	0.021404
AAEL018189	56.956	-1.23673	0.320701	-3.85634	0.000115	0.02325
AAEL006377	320.5194	-0.92791	0.241602	-3.84068	0.000123	0.023637
AAEL013432	206.882	-1.04263	0.273691	-3.80951	0.000139	0.025703
AAEL020035	135.6844	-1.18964	0.321919	-3.69547	0.000219	0.030463
AAEL025334	93.20759	-1.20526	0.327448	-3.68077	0.000233	0.031228

FC: fold change; P-adj: adjusted *p* value

7 dpi 28 °C upregulated

GeneID	Base mean	log2(FC)	StdErr	Wald-Stats	P-value	P-adj
AAEL013350	728.892	3.116381	0.297873	10.4621	1.29E-25	1.52E-21
AAEL017975	2959.508	3.074894	0.299122	10.27974	8.70E-25	5.11E-21
AAEL006883	667.5833	2.292157	0.240929	9.513818	1.84E-21	5.40E-18
AAEL023591	2032.563	1.554986	0.163415	9.515592	1.81E-21	5.40E-18
AAEL009645	5142.394	1.068227	0.115341	9.261451	2.02E-20	4.74E-17
AAEL010434	178.1841	2.635448	0.30056	8.76845	1.81E-18	3.55E-15
AAEL020330	328.7641	2.522	0.303941	8.29765	1.06E-16	1.78E-13
AAEL013346	171.5745	2.440115	0.302939	8.054812	7.96E-16	1.17E-12
AAEL025531	99.93837	2.307221	0.292246	7.894794	2.91E-15	3.80E-12
AAEL002655	139.2552	2.295781	0.296012	7.755711	8.79E-15	9.82E-12
AAEL025126	151.0833	2.294829	0.296109	7.749954	9.19E-15	9.82E-12
AAEL022079	113.6611	1.816592	0.235693	7.707444	1.28E-14	1.16E-11
AAEL026008	2540.816	1.846535	0.239494	7.710156	1.26E-14	1.16E-11
AAEL010068	1021.116	1.601684	0.209201	7.656194	1.92E-14	1.61E-11
AAEL017976	1384.711	2.24417	0.303548	7.393134	1.43E-13	1.12E-10
AAEL009387	7637.988	0.653415	0.089925	7.266183	3.70E-13	2.72E-10
AAEL004169	367.0314	1.321907	0.184538	7.163348	7.87E-13	5.44E-10
AAEL007902	185.6905	1.35207	0.189582	7.131846	9.90E-13	6.47E-10
AAEL022253	1757.554	2.143421	0.303135	7.07085	1.54E-12	9.53E-10
AAEL003505	2334.232	1.719665	0.244088	7.04527	1.85E-12	1.09E-09
AAEL017380	93.3928	2.127009	0.302186	7.038741	1.94E-12	1.09E-09
AAEL021302	473.302	1.652228	0.235395	7.01897	2.24E-12	1.19E-09
AAEL009487	3705.967	0.796327	0.113736	7.001517	2.53E-12	1.29E-09
AAEL008622	67.37195	1.985214	0.289337	6.861246	6.83E-12	3.34E-09
AAEL011371	4347.899	0.98818	0.147226	6.711997	1.92E-11	9.03E-09

GeneID	Base mean	log2(FC)	StdErr	Wald-Stats	P-value	P-adj
AAEL003728	250.8779	1.682361	0.255804	6.576753	4.81E-11	2.10E-08
AAEL003345	5246.289	1.764473	0.268303	6.57643	4.82E-11	2.10E-08
AAEL007126	122.7807	1.415538	0.216427	6.540483	6.13E-11	2.49E-08
AAEL010769	738.499	1.296685	0.198184	6.54284	6.04E-11	2.49E-08
AAEL013348	58.85481	1.851582	0.287246	6.445975	1.15E-10	4.50E-08
AAEL006126	71.78147	1.881567	0.296864	6.338145	2.33E-10	8.82E-08
AAEL024838	419.5059	1.126007	0.178562	6.305962	2.86E-10	1.05E-07
AAEL010379	398.7471	1.350192	0.214908	6.28266	3.33E-10	1.19E-07
AAEL004688	2124.404	0.60024	0.096003	6.252293	4.04E-10	1.40E-07
AAEL006904	1299.323	0.695429	0.11258	6.177196	6.53E-10	2.19E-07
AAEL009171	1750.662	0.833242	0.138185	6.029908	1.64E-09	5.36E-07
AAEL019463	327.5993	0.914593	0.15194	6.01944	1.75E-09	5.56E-07
AAEL001857	81.81225	1.553	0.262458	5.917143	3.28E-09	9.87E-07
AAEL008635	788.3385	1.493264	0.255311	5.848804	4.95E-09	1.46E-06
AAEL002610	1599.883	1.588379	0.273095	5.816219	6.02E-09	1.73E-06
AAEL021929	320.0132	1.748408	0.303424	5.762268	8.30E-09	2.32E-06
AAEL015631	557.4039	1.066857	0.186659	5.715537	1.09E-08	2.99E-06
AAEL013857	245.9264	1.518453	0.26597	5.709103	1.14E-08	3.03E-06
AAEL001420	5636.799	1.109987	0.195588	5.675132	1.39E-08	3.62E-06
AAEL026603	38.27262	1.597974	0.284453	5.617714	1.93E-08	4.94E-06
AAEL008953	8775.947	0.709779	0.126438	5.613638	1.98E-08	4.96E-06
AAEL009126	64.96642	1.676161	0.301159	5.565701	2.61E-08	6.39E-06
AAEL019751	508.2229	1.160973	0.209594	5.53914	3.04E-08	7.29E-06
AAEL026833	890.4588	1.575777	0.284982	5.529398	3.21E-08	7.55E-06
AAEL023321	107.0405	1.434187	0.260189	5.512088	3.55E-08	8.02E-06
AAEL001929	32.82776	1.346273	0.244091	5.515464	3.48E-08	8.02E-06
AAEL004803	107.9252	0.861928	0.156547	5.505866	3.67E-08	8.15E-06
AAEL015465	208.0624	1.260426	0.229887	5.482799	4.19E-08	9.11E-06
AAEL012856	434.0041	1.590409	0.290414	5.476358	4.34E-08	9.28E-06
AAEL027610	762.9641	1.629492	0.298347	5.461729	4.72E-08	9.90E-06
AAEL005768	252.2436	1.283613	0.235575	5.448853	5.07E-08	1.05E-05
AAEL009762	149.2643	1.509447	0.278382	5.422207	5.89E-08	1.19E-05
AAEL002969	586.2887	1.410403	0.263625	5.35004	8.79E-08	1.75E-05
AAEL006138	126.1631	1.598621	0.299556	5.336639	9.47E-08	1.86E-05
AAEL001414	3151.493	1.219908	0.232022	5.25773	1.46E-07	2.77E-05
AAEL004048	281.9358	1.006605	0.192029	5.241931	1.59E-07	2.96E-05
AAEL007197	782.7576	0.689791	0.132277	5.214737	1.84E-07	3.28E-05
AAEL002499	573.4986	0.883405	0.170364	5.185385	2.16E-07	3.78E-05
AAEL012089	770.8618	0.992716	0.193761	5.123406	3.00E-07	5.19E-05
AAEL008887	522.4587	1.43256	0.280674	5.103993	3.33E-07	5.67E-05
AAEL009055	279.3157	1.151009	0.227245	5.065057	4.08E-07	6.86E-05
AAEL007969	72.9558	1.186819	0.235566	5.038153	4.70E-07	7.75E-05
AAEL017345	1159.848	1.19892	0.238336	5.030378	4.90E-07	7.88E-05
AAEL008050	52.41532	1.521366	0.30315	5.018518	5.21E-07	8.27E-05
AAEL005992	109.7268	1.470094	0.29646	4.958831	7.09E-07	0.000107

GeneID	Base mean	log2(FC)	StdErr	Wald-Stats	P-value	P-adj
AAEL006543	353.2948	0.734391	0.14816	4.956759	7.17E-07	0.000107
AAEL015298	1103.794	0.754626	0.152215	4.957644	7.14E-07	0.000107
AAEL011038	519.7914	1.035706	0.209131	4.95242	7.33E-07	0.000108
AAEL003888	1267.242	1.231022	0.249439	4.935158	8.01E-07	0.000116
AAEL021555	163.3378	0.930546	0.190445	4.886154	1.03E-06	0.000147
AAEL014138	109.9276	0.956871	0.195996	4.882103	1.05E-06	0.000149
AAEL003597	194.5737	0.994111	0.203906	4.875352	1.09E-06	0.000152
AAEL006269	221.219	0.930504	0.19112	4.868691	1.12E-06	0.000155
AAEL013840	114.4479	0.757733	0.156048	4.855775	1.20E-06	0.000164
AAEL008473	1848.89	1.401784	0.288906	4.852041	1.22E-06	0.000165
AAEL018343	221.6811	0.975159	0.201241	4.845725	1.26E-06	0.000169
AAEL013984	1037.616	1.090209	0.225189	4.841296	1.29E-06	0.00017
AAEL018340	214.9941	0.958824	0.198471	4.831046	1.36E-06	0.000174
AAEL014618	40.02552	1.446133	0.300669	4.809722	1.51E-06	0.000191
AAEL011453	102.1792	1.238665	0.258308	4.795309	1.62E-06	0.000203
AAEL021099	252.744	1.132791	0.237184	4.77601	1.79E-06	0.000214
AAEL008471	396.7432	0.640615	0.134893	4.749073	2.04E-06	0.000243
AAEL005763	427.0991	0.75767	0.160337	4.725491	2.30E-06	0.00027
AAEL001794	1045.009	1.133738	0.241373	4.697033	2.64E-06	0.000301
AAEL006815	180.6059	0.928441	0.197772	4.69449	2.67E-06	0.000302
AAEL010102	242.2174	0.684095	0.145861	4.690049	2.73E-06	0.000303
AAEL007773	256.9152	0.780883	0.166465	4.690962	2.72E-06	0.000303
AAEL013770	53.71287	1.269363	0.272387	4.660153	3.16E-06	0.000347
AAEL008106	809.437	1.12908	0.242515	4.655707	3.23E-06	0.000351
AAEL022059	926.7941	1.340581	0.28813	4.65269	3.28E-06	0.000353
AAEL003051	58.52314	0.828919	0.180728	4.586556	4.51E-06	0.000469
AAEL003527	40.58973	0.877781	0.192604	4.557451	5.18E-06	0.00053
AAEL001965	752.0782	1.124173	0.246693	4.556964	5.19E-06	0.00053
AAEL002124	737.2991	0.996084	0.218918	4.550032	5.36E-06	0.000539
AAEL006990	541.2391	1.19069	0.262658	4.533239	5.81E-06	0.000579
AAEL024512	169.479	1.362707	0.30104	4.526671	5.99E-06	0.000587
AAEL003640	290.3398	0.783619	0.173109	4.526736	5.99E-06	0.000587
AAEL013345	270.9954	1.298092	0.289007	4.491561	7.07E-06	0.000687
AAEL024583	1618.893	0.784361	0.175192	4.477154	7.56E-06	0.000729
AAEL025903	169.268	1.038508	0.235003	4.419121	9.91E-06	0.000932
AAEL026175	1736.262	1.062213	0.240835	4.410539	1.03E-05	0.000962
AAEL001575	27.2747	0.993797	0.22556	4.405901	1.05E-05	0.000975
AAEL009863	1853.093	0.697844	0.159377	4.378583	1.19E-05	0.001097
AAEL008495	295.8199	0.829751	0.190175	4.363096	1.28E-05	0.001161
AAEL026300	26.30127	1.25414	0.287464	4.362779	1.28E-05	0.001161
AAEL019537	189.0761	1.020824	0.234192	4.358914	1.31E-05	0.001173
AAEL008963	629.9184	0.84189	0.193384	4.353455	1.34E-05	0.001193
AAEL010206	334.0111	1.097843	0.252299	4.351361	1.35E-05	0.001196
AAEL007092	1327.612	0.596863	0.137465	4.341927	1.41E-05	0.00123
AAEL005533	51.15832	1.313907	0.303443	4.329991	1.49E-05	0.001286

GeneID	Base mean	log2(FC)	StdErr	Wald-Stats	P-value	P-adj
AAEL000304	707.0519	0.655257	0.15137	4.328856	1.50E-05	0.001286
AAEL012712	187.5658	1.231312	0.284744	4.324269	1.53E-05	0.001288
AAEL014566	133.3158	0.980285	0.227344	4.311902	1.62E-05	0.001331
AAEL002600	1788.124	0.798765	0.185465	4.306818	1.66E-05	0.001343
AAEL013554	18.85148	1.134401	0.264411	4.290301	1.78E-05	0.001417
AAEL002049	670.6905	0.899753	0.209911	4.286343	1.82E-05	0.001433
AAEL006576	1406.726	1.045628	0.24444	4.277641	1.89E-05	0.00147
AAEL008625	52.39674	0.851006	0.198909	4.278372	1.88E-05	0.00147
AAEL001232	297.9011	0.685302	0.160411	4.272175	1.94E-05	0.001487
AAEL007128	277.9654	1.032843	0.24215	4.265311	2.00E-05	0.001519
AAEL024291	93.41137	0.695878	0.163489	4.256417	2.08E-05	0.001545
AAEL002959	109.1231	1.199275	0.282411	4.246553	2.17E-05	0.001585
AAEL003426	36.92346	0.998294	0.235068	4.246838	2.17E-05	0.001585
AAEL019658	968.931	0.624681	0.148328	4.211477	2.54E-05	0.001812
AAEL023524	233.7648	0.843538	0.20032	4.210947	2.54E-05	0.001812
AAEL017023	718.0172	0.98708	0.2347	4.205699	2.60E-05	0.001831
AAEL011810	461.4027	0.89686	0.213235	4.205963	2.60E-05	0.001831
AAEL027700	141.6591	1.000672	0.238004	4.204441	2.62E-05	0.001831
AAEL008028	607.322	0.78128	0.185952	4.201517	2.65E-05	0.001841
AAEL010151	275.9798	0.702215	0.167171	4.20059	2.66E-05	0.001841
AAEL026843	5521.472	0.957165	0.229077	4.178348	2.94E-05	0.002001
AAEL013163	811.1976	0.797777	0.191223	4.171964	3.02E-05	0.00204
AAEL013339	16.58957	1.210306	0.29106	4.15827	3.21E-05	0.002142
AAEL018334	329.0879	0.681031	0.164547	4.138836	3.49E-05	0.002318
AAEL002467	1059.453	1.249607	0.302181	4.135296	3.54E-05	0.002341
AAEL005660	209.4714	0.626625	0.151797	4.128043	3.66E-05	0.002389
AAEL001307	44.62804	0.890785	0.216366	4.117035	3.84E-05	0.002492
AAEL006794	3469.437	0.666262	0.162045	4.111581	3.93E-05	0.002538
AAEL008190	277.0882	0.807751	0.197677	4.086228	4.38E-05	0.00275
AAEL007619	401.9259	0.896309	0.220135	4.07163	4.67E-05	0.002888
AAEL021375	213.6006	1.039871	0.255942	4.062915	4.85E-05	0.002952
AAEL001646	64.59255	1.091138	0.2685	4.063831	4.83E-05	0.002952
AAEL008144	2003.838	0.73361	0.180981	4.053512	5.05E-05	0.003057
AAEL019564	326.2307	0.896106	0.221539	4.044918	5.23E-05	0.003139
AAEL012981	262.2759	0.626864	0.155021	4.043747	5.26E-05	0.003139
AAEL014348	118.0114	0.813352	0.201371	4.039074	5.37E-05	0.003186
AAEL021263	89.96674	1.04891	0.260213	4.030975	5.55E-05	0.003265
AAEL022932	62.93918	1.037877	0.258007	4.022672	5.75E-05	0.00335
AAEL028635	201.4996	1.205347	0.299882	4.019398	5.83E-05	0.003379
AAEL003726	32.35003	1.206955	0.30326	3.979935	6.89E-05	0.003915
AAEL022674	62.18833	1.134412	0.285747	3.96999	7.19E-05	0.004062
AAEL008157	110.7213	0.937194	0.23688	3.956402	7.61E-05	0.004239
AAEL003139	113.5658	0.738432	0.187222	3.94415	8.01E-05	0.00444
AAEL003844	1921.863	0.607159	0.154337	3.933972	8.36E-05	0.004611
AAEL009213	373.5242	0.792267	0.201455	3.932736	8.40E-05	0.004613

GeneID	Base mean	log2(FC)	StdErr	Wald-Stats	P-value	P-adj
AAEL000049	182.7444	0.71724	0.182497	3.930155	8.49E-05	0.004641
AAEL000037	254.1255	0.967964	0.246773	3.922491	8.76E-05	0.004769
AAEL005017	892.4331	0.616856	0.15761	3.913805	9.09E-05	0.00488
AAEL027188	30.93828	1.128159	0.288263	3.913649	9.09E-05	0.00488
AAEL007444	5000.773	0.617793	0.158059	3.908614	9.28E-05	0.00496
AAEL011452	136.996	0.865411	0.221666	3.904129	9.46E-05	0.00503
AAEL006069	299.3025	0.68483	0.175563	3.900773	9.59E-05	0.005077
AAEL024337	420.0972	0.656047	0.168875	3.884807	0.000102	0.005342
AAEL006535	160.339	0.974352	0.250856	3.884107	0.000103	0.005342
AAEL003632	26.05914	1.170244	0.301375	3.883012	0.000103	0.005343
AAEL002741	37.61778	0.861112	0.222433	3.871337	0.000108	0.005581
AAEL003626	245.7445	0.931098	0.240752	3.867457	0.00011	0.005621
AAEL007191	182.0107	0.618911	0.160236	3.862503	0.000112	0.005662
AAEL002683	650.203	0.619203	0.160689	3.853431	0.000116	0.005851
AAEL001960	341.1649	0.795728	0.207012	3.843878	0.000121	0.005963
AAEL001417	154.6086	1.080971	0.281668	3.83775	0.000124	0.006074
AAEL014605	1723.851	0.905679	0.236037	3.837017	0.000125	0.006074
AAEL001864	4113.008	0.986691	0.257224	3.835927	0.000125	0.006076
AAEL028247	20.69271	1.164048	0.30383	3.831245	0.000127	0.006168
AAEL017334	2081.961	1.094645	0.285838	3.829592	0.000128	0.006184
AAEL008365	401.2089	1.002519	0.262019	3.826133	0.00013	0.00622
AAEL007778	4061.046	0.834316	0.21842	3.819778	0.000134	0.006303
AAEL013347	602.0945	1.011676	0.265541	3.809868	0.000139	0.00646
AAEL010688	103.3768	0.757898	0.19934	3.802038	0.000144	0.006616
AAEL023844	216.4577	1.008718	0.265749	3.795751	0.000147	0.006707
AAEL017144	1478.453	1.0164	0.267694	3.796878	0.000147	0.006707
AAEL010884	2076.732	1.075631	0.283806	3.790025	0.000151	0.006837
AAEL017139	58.74374	0.971667	0.258125	3.764321	0.000167	0.007493
AAEL026751	4615.016	0.765989	0.203747	3.759505	0.00017	0.007608
AAEL020502	214.3529	0.791625	0.210813	3.755111	0.000173	0.007686
AAEL014391	111.498	0.851273	0.227605	3.740132	0.000184	0.008079
AAEL012853	159.8212	0.851246	0.227676	3.738844	0.000185	0.008079
AAEL022775	49.32167	1.080662	0.29047	3.720391	0.000199	0.008628
AAEL002385	297.9651	0.681258	0.183269	3.717264	0.000201	0.008672
AAEL020392	13.62418	1.128363	0.303514	3.717661	0.000201	0.008672
AAEL002893	24.91057	1.043772	0.28233	3.696988	0.000218	0.009225
AAEL002390	743.3171	0.706725	0.191132	3.697575	0.000218	0.009225
AAEL027984	23.0876	0.974827	0.264064	3.691631	0.000223	0.009369
AAEL010596	104.2237	0.869562	0.235581	3.691133	0.000223	0.009369
AAEL017513	366.3585	0.98735	0.267601	3.689633	0.000225	0.009369
AAEL013732	72.55543	0.959131	0.260256	3.685339	0.000228	0.009412
AAEL027545	21.0711	0.871389	0.236605	3.682891	0.000231	0.009412
AAEL006805	1232.235	0.846908	0.229933	3.683282	0.00023	0.009412
AAEL003737	145.1071	0.698272	0.190192	3.671412	0.000241	0.009767
AAEL012702	2185.461	0.949385	0.259582	3.657367	0.000255	0.010223

GeneID	Base mean	log2(FC)	StdErr	Wald-Stats	P-value	P-adj
AAEL005731	1630.859	0.759913	0.207874	3.655648	0.000257	0.010257
AAEL011365	87.11608	0.63695	0.174387	3.652504	0.00026	0.010348
AAEL025921	1053.944	0.622837	0.170665	3.649471	0.000263	0.010436
AAEL013853	17.96277	0.924485	0.253415	3.648101	0.000264	0.010456
AAEL002412	789.0255	0.700523	0.192256	3.6437	0.000269	0.010587
AAEL010269	161.0076	0.789138	0.216695	3.641693	0.000271	0.010613
AAEL010180	593.7807	1.097152	0.301525	3.638676	0.000274	0.010667
AAEL009629	2065.598	0.657224	0.180667	3.63777	0.000275	0.010669
AAEL000834	472.9553	0.906143	0.249399	3.633301	0.00028	0.01082
AAEL029061	63.28002	0.897938	0.247607	3.62647	0.000287	0.011037
AAEL006563	32.15283	0.977453	0.270784	3.609721	0.000307	0.011586
AAEL009955	15274.56	0.903363	0.250374	3.608057	0.000308	0.011623
AAEL010773	257.6855	0.90279	0.250571	3.602929	0.000315	0.011817
AAEL012395	319.2499	0.933424	0.25932	3.599504	0.000319	0.011919
AAEL005738	133.9378	0.856895	0.238204	3.597308	0.000322	0.011919
AAEL007342	1814.734	0.735001	0.204246	3.59861	0.00032	0.011919
AAEL005921	200.5262	0.632953	0.175988	3.596577	0.000322	0.011919
AAEL007808	124.4748	0.747978	0.207877	3.598185	0.00032	0.011919
AAEL013351	301.1988	1.029996	0.28754	3.582098	0.000341	0.012482
AAEL006179	57.31212	1.082713	0.302352	3.580964	0.000342	0.012497
AAEL012513	352.2005	0.593043	0.165671	3.57964	0.000344	0.012522
AAEL021257	60.41726	1.000669	0.279999	3.57383	0.000352	0.012646
AAEL023294	4593.122	0.663776	0.186367	3.561659	0.000369	0.013207
AAEL001189	8.51749	1.045852	0.294116	3.555914	0.000377	0.013336
AAEL002254	513.1662	0.670733	0.188927	3.550224	0.000385	0.013529
AAEL005386	42.92092	0.658306	0.185548	3.547905	0.000388	0.013545
AAEL007878	65.59102	0.89719	0.252866	3.548086	0.000388	0.013545
AAEL020936	343.6	0.582133	0.164269	3.543785	0.000394	0.013717
AAEL014354	131.8967	0.861564	0.243493	3.538354	0.000403	0.013961
AAEL023125	173.2256	0.847134	0.239761	3.533246	0.00041	0.014192
AAEL015430	98.33715	0.812388	0.230083	3.530845	0.000414	0.014269
AAEL001582	257.3216	0.721753	0.204867	3.523028	0.000427	0.014495
AAEL002130	2551.637	0.667593	0.189437	3.524081	0.000425	0.014495
AAEL007856	23.18957	1.069977	0.303915	3.520648	0.00043	0.014583
AAEL008828	126.6542	0.869882	0.248012	3.507423	0.000452	0.015107
AAEL003316	175.307	0.660549	0.18852	3.503866	0.000459	0.015213
AAEL013525	405.2231	0.63866	0.182583	3.497919	0.000469	0.01544
AAEL021073	843.091	0.77509	0.221849	3.493771	0.000476	0.015594
AAEL026446	374.8875	0.842943	0.241556	3.489634	0.000484	0.01575
AAEL006434	208.574	0.625826	0.179421	3.48803	0.000487	0.015801
AAEL002624	261.4895	0.954202	0.273828	3.484679	0.000493	0.015956
AAEL007624	598.2947	0.721101	0.207027	3.483124	0.000496	0.016005
AAEL008007	415.0432	1.054705	0.303474	3.475443	0.00051	0.016258
AAEL005428	3100.073	0.935934	0.269743	3.469732	0.000521	0.016503
AAEL021795	2528.497	0.933923	0.269263	3.468443	0.000523	0.016503

GeneID	Base mean	log2(FC)	StdErr	Wald-Stats	P-value	P-adj
AAEL004833	57.25577	0.988494	0.285181	3.466203	0.000528	0.016503
AAEL007296	445.058	0.930626	0.26835	3.467957	0.000524	0.016503
AAEL024406	1464.12	0.839349	0.242338	3.463546	0.000533	0.016577
AAEL006323	218.9261	1.029202	0.297273	3.462147	0.000536	0.016577
AAEL013027	5.590501	0.974992	0.282532	3.450913	0.000559	0.017063
AAEL024540	136.8602	0.788553	0.228667	3.448484	0.000564	0.017077
AAEL004213	29.94395	0.939	0.272346	3.447821	0.000565	0.017077
AAEL022982	513.8749	0.704866	0.204489	3.446954	0.000567	0.017088
AAEL005959	26.68813	1.008493	0.29272	3.445255	0.000571	0.017108
AAEL022829	5.715702	1.042455	0.302657	3.444348	0.000572	0.017122
AAEL019487	1582.578	0.625554	0.181687	3.443021	0.000575	0.017163
AAEL011203	61.36906	0.941392	0.273753	3.438838	0.000584	0.017383
AAEL014255	83.20852	0.892642	0.259624	3.438207	0.000586	0.017383
AAEL024269	23.30053	0.959329	0.279297	3.434806	0.000593	0.017558
AAEL023847	24.28288	0.975997	0.284341	3.432486	0.000598	0.017664
AAEL006723	228.1399	0.752672	0.219584	3.427714	0.000609	0.017901
AAEL013532	227.3889	0.594484	0.174487	3.407036	0.000657	0.018968
AAEL006795	153.574	0.867536	0.255318	3.397866	0.000679	0.01933
AAEL019954	52.71657	1.023466	0.301517	3.39439	0.000688	0.01953
AAEL015644	201.0627	0.77547	0.22864	3.391657	0.000695	0.019678
AAEL000243	37.70593	1.020635	0.301085	3.389863	0.000699	0.01976
AAEL021016	159.3543	0.62619	0.18479	3.388663	0.000702	0.019799
AAEL003645	1160.819	0.82356	0.243852	3.377298	0.000732	0.020247
AAEL007090	140.8638	0.932203	0.275979	3.377807	0.000731	0.020247
AAEL013225	1609.375	0.658488	0.194959	3.377564	0.000731	0.020247
AAEL017403	12.0762	0.899032	0.266321	3.375748	0.000736	0.020266
AAEL000811	265.837	0.869935	0.257853	3.373758	0.000741	0.020365
AAEL008961	149.261	0.626215	0.186067	3.365535	0.000764	0.020884
AAEL024753	88.15822	0.931237	0.276856	3.363614	0.000769	0.020933
AAEL013613	2085.21	0.701672	0.208757	3.361196	0.000776	0.021068
AAEL023100	28.37116	0.794484	0.236456	3.359968	0.00078	0.021113
AAEL014350	60.52949	0.992138	0.295426	3.358326	0.000784	0.021118
AAEL013257	40.17639	0.589155	0.175389	3.35913	0.000782	0.021118
AAEL007926	321.0101	0.935373	0.279236	3.349759	0.000809	0.021658
AAEL001586	273.0323	0.640416	0.191583	3.342756	0.00083	0.022066
AAEL012726	109.2739	0.638451	0.191096	3.340986	0.000835	0.02212
AAEL007794	40.43803	0.905844	0.27115	3.340753	0.000836	0.02212
AAEL014045	182.8779	0.798563	0.239355	3.336312	0.000849	0.022426
AAEL009936	29.19303	0.850153	0.255156	3.331887	0.000863	0.022583
AAEL002765	305.1896	0.610712	0.183396	3.330018	0.000868	0.022634
AAEL013434	240.1297	0.588193	0.178012	3.304225	0.000952	0.024338
AAEL006585	279.2277	0.834265	0.252665	3.301864	0.00096	0.024358
AAEL013341	791.4733	0.745544	0.225753	3.302475	0.000958	0.024358
AAEL009695	105.5119	0.754852	0.228634	3.301569	0.000961	0.024358
AAEL009952	99.87689	0.798505	0.242279	3.295807	0.000981	0.024809

GeneID	Base mean	log2(FC)	StdErr	Wald-Stats	P-value	P-adj
AAEL007907	116.1941	0.804487	0.244428	3.291313	0.000997	0.025047
AAEL004701	126.522	0.955274	0.290841	3.284526	0.001022	0.025573
AAEL019847	5.289382	0.994898	0.302984	3.283663	0.001025	0.025573
AAEL011177	45.18812	0.799456	0.243741	3.279943	0.001038	0.025858
AAEL010671	24.07153	0.894153	0.27301	3.275168	0.001056	0.026133
AAEL005749	251.0069	0.686759	0.210396	3.26412	0.001098	0.027003
AAEL010076	104.2662	0.757702	0.232393	3.260436	0.001112	0.027242
AAEL004673	175.841	0.747257	0.229475	3.256369	0.001128	0.027521
AAEL006361	16.80501	0.972718	0.298805	3.255364	0.001132	0.027562
AAEL025296	65.94521	0.933498	0.286871	3.254068	0.001138	0.02763
AAEL003738	143.0541	0.947385	0.29123	3.25305	0.001142	0.027672
AAEL000663	686.7743	0.701535	0.215908	3.249228	0.001157	0.027989
AAEL001184	113.6894	0.715922	0.220444	3.247643	0.001164	0.028057
AAEL004344	61.92219	0.946255	0.291754	3.243337	0.001181	0.028399
AAEL021583	361.7207	0.644956	0.199062	3.239973	0.001195	0.028678
AAEL003067	199.4711	0.735418	0.227297	3.235489	0.001214	0.029013
AAEL005753	253.4142	0.903745	0.279277	3.236021	0.001212	0.029013
AAEL009232	719.261	0.59774	0.184799	3.234544	0.001218	0.029051
AAEL029031	210.3387	0.899357	0.278608	3.228032	0.001246	0.02965
AAEL014662	100.3897	0.820568	0.254284	3.226967	0.001251	0.02965
AAEL002853	353.8561	0.783151	0.242955	3.223439	0.001267	0.029958
AAEL010125	452.0773	0.827231	0.257058	3.218076	0.001291	0.03028
AAEL022387	1555.035	0.839831	0.261106	3.216431	0.001298	0.030333
AAEL003516	35.32607	0.818741	0.254859	3.21252	0.001316	0.030688
AAEL010451	55.59873	0.883062	0.275001	3.211126	0.001322	0.030776
AAEL007806	95.1836	0.824052	0.256984	3.20662	0.001343	0.031017
AAEL008404	15.78988	0.927311	0.290059	3.196973	0.001389	0.031823
AAEL003611	503.1464	0.749426	0.235086	3.187881	0.001433	0.032587
AAEL019955	56.06252	0.818249	0.257095	3.182672	0.001459	0.03305
AAEL008936	505.5039	0.897201	0.282588	3.174942	0.001499	0.033684
AAEL001402	1624.63	0.803909	0.253299	3.173761	0.001505	0.033757
AAEL002394	68.52411	0.964397	0.303931	3.173083	0.001508	0.033771
AAEL020430	53.68024	0.869754	0.274401	3.169644	0.001526	0.03398
AAEL018150	256.8273	0.637242	0.201189	3.167374	0.001538	0.034052
AAEL028228	71.79854	0.60176	0.190749	3.154719	0.001607	0.035037
AAEL008628	11.60906	0.936794	0.297523	3.148643	0.00164	0.035707
AAEL019527	98.32211	0.936215	0.297447	3.147499	0.001647	0.035715
AAEL010772	12.71458	0.935409	0.297307	3.146271	0.001654	0.035733
AAEL004513	501.493	0.672957	0.214319	3.139977	0.00169	0.036243
AAEL004581	115.7052	0.747296	0.238105	3.138521	0.001698	0.036292
AAEL006754	188.9597	0.729288	0.233323	3.125656	0.001774	0.037643
AAEL007603	91.07154	0.790994	0.253281	3.122995	0.00179	0.037917
AAEL013352	165.8765	0.899788	0.28834	3.120586	0.001805	0.038091
AAEL001519	89.37989	0.754631	0.241797	3.120924	0.001803	0.038091
AAEL010480	171.9286	0.712333	0.228505	3.117365	0.001825	0.038304

GeneID	Base mean	log2(FC)	StdErr	Wald-Stats	P-value	P-adj
AAEL007807	291.0773	0.709335	0.227497	3.118003	0.001821	0.038304
AAEL002964	372.1749	0.708892	0.227773	3.112275	0.001857	0.038639
AAEL003978	86.0666	0.734852	0.23628	3.11009	0.00187	0.038639
AAEL012099	1071.938	0.644528	0.207692	3.103283	0.001914	0.039126
AAEL006027	369.2281	0.712718	0.229633	3.103733	0.001911	0.039126
AAEL003954	244.363	0.899993	0.290701	3.095942	0.001962	0.0399
AAEL025177	7.287182	0.937595	0.30297	3.094679	0.00197	0.040001
AAEL002031	364.7844	0.662513	0.214363	3.090608	0.001997	0.040414
AAEL019752	96.5247	0.935317	0.302789	3.089012	0.002008	0.040456
AAEL024216	36.26313	0.814318	0.263575	3.089518	0.002005	0.040456
AAEL007131	41.35659	0.902229	0.2921	3.088769	0.00201	0.040456
AAEL007830	93.73634	0.803854	0.260699	3.083455	0.002046	0.040975
AAEL013367	27.47528	0.830021	0.26916	3.08375	0.002044	0.040975
AAEL002353	44.52078	0.883784	0.287199	3.077257	0.002089	0.041765
AAEL002397	270.8589	0.672917	0.218925	3.073726	0.002114	0.042119
AAEL013111	417.0919	0.606105	0.197237	3.072975	0.002119	0.042154
AAEL000786	663.9844	0.613819	0.199955	3.069792	0.002142	0.04253
AAEL026214	54.25816	0.931681	0.303943	3.065316	0.002174	0.042958
AAEL012110	1114.376	0.680283	0.222055	3.063574	0.002187	0.043064
AAEL009112	150.0326	0.75735	0.247619	3.058532	0.002224	0.043563
AAEL002288	177.7313	0.701476	0.229506	3.056461	0.00224	0.043733
AAEL010164	136.0554	0.926719	0.303441	3.054033	0.002258	0.044015
AAEL022578	1111.711	0.651825	0.213518	3.052779	0.002267	0.044109
AAEL026481	16.45832	0.923289	0.302529	3.051906	0.002274	0.044109
AAEL014426	613.9428	0.78692	0.258058	3.049398	0.002293	0.044333
AAEL011130	220.0641	0.84931	0.278737	3.046994	0.002311	0.044542
AAEL010960	497.0209	0.590991	0.194244	3.042514	0.002346	0.044989
AAEL006682	268.142	0.600672	0.19767	3.038753	0.002376	0.045455
AAEL024717	43.29953	0.7425	0.245675	3.022284	0.002509	0.04726
AAEL022593	36.32292	0.748915	0.247928	3.020691	0.002522	0.047355
AAEL013349	138.7392	0.916768	0.303543	3.020225	0.002526	0.047355
AAEL001503	250.6286	0.616026	0.203919	3.02094	0.00252	0.047355
AAEL007792	188.4432	0.663433	0.219998	3.015624	0.002565	0.047905
AAEL014349	162.4543	0.819806	0.271922	3.014853	0.002571	0.047905
AAEL020706	171.17	0.799672	0.265249	3.014796	0.002572	0.047905
AAEL019917	182.4769	0.863065	0.287364	3.003385	0.00267	0.049348
AAEL027068	16.06901	0.910432	0.303506	2.999714	0.002702	0.049542
AAEL009483	396.3347	0.624344	0.208195	2.998836	0.00271	0.049542
AAEL002632	20.76006	0.853735	0.28468	2.998925	0.002709	0.049542
AAEL005142	139.5816	0.697069	0.232773	2.994635	0.002748	0.049768
AAEL000650	518.4899	0.829376	0.277	2.994139	0.002752	0.049773

FC: fold change; P-adj: adjusted *p* value

7 dpi 28 °C downregulated

GeneID	Base mean	log2(FC)	StdErr	Wald-Stats	P-value	P-adj
AAEL006539	796.8858	-0.73678	0.152472	-4.83225	1.35E-06	0.000174
AAEL006548	103.4199	-0.9033	0.196717	-4.59187	4.39E-06	0.000461
AAEL001985	31.22873	-0.89225	0.206364	-4.32368	1.53E-05	0.001288
AAEL019885	4768.937	-0.67283	0.157481	-4.27249	1.93E-05	0.001487
AAEL021308	699.5122	-0.83538	0.197223	-4.2357	2.28E-05	0.001653
AAEL012725	4190.36	-0.63749	0.152979	-4.16718	3.08E-05	0.002072
AAEL024284	92.68213	-1.24023	0.303653	-4.08437	4.42E-05	0.00275
AAEL003945	1538.941	-0.59791	0.14691	-4.06988	4.70E-05	0.002895
AAEL007472	152.4488	-0.75298	0.185976	-4.04879	5.15E-05	0.003103
AAEL015566	13.39188	-1.16857	0.302098	-3.86818	0.00011	0.005621
AAEL019889	315.843	-0.78786	0.205865	-3.82706	0.00013	0.00622
AAEL018234	311.1289	-0.76938	0.201293	-3.82217	0.000132	0.00627
AAEL012762	38.69028	-0.82461	0.21902	-3.76501	0.000167	0.007493
AAEL021449	19.00106	-1.12455	0.303932	-3.7	0.000216	0.009183
AAEL024757	12.57881	-1.117	0.302825	-3.68858	0.000226	0.009369
AAEL006538	1261.145	-0.72467	0.199573	-3.63111	0.000282	0.010876
AAEL005403	195.1592	-0.72627	0.204596	-3.54977	0.000386	0.013529
AAEL002173	46.9797	-1.07221	0.303871	-3.52849	0.000418	0.014323
AAEL011023	5220.941	-0.61058	0.175352	-3.48202	0.000498	0.016027
AAEL012377	182.5946	-0.87641	0.252186	-3.47526	0.00051	0.016258
AAEL006556	1382.892	-0.7928	0.232545	-3.40923	0.000651	0.018955
AAEL026107	37.50244	-0.95965	0.283578	-3.38407	0.000714	0.019989
AAEL022124	30.82317	-1.01051	0.300338	-3.36458	0.000767	0.020908
AAEL001997	605.8789	-0.68818	0.206507	-3.33248	0.000861	0.022583
AAEL008454	117.2711	-0.96631	0.292072	-3.30848	0.000938	0.024129
AAEL020075	275.3158	-0.93843	0.286306	-3.27772	0.001046	0.026007
AAEL010362	138.3527	-0.74182	0.230461	-3.21886	0.001287	0.030257
AAEL002753	972.7655	-0.88924	0.278464	-3.19339	0.001406	0.032095
AAEL019629	348.0743	-0.96619	0.303852	-3.17982	0.001474	0.03325
AAEL009777	23.26452	-0.93079	0.292797	-3.17897	0.001478	0.033283
AAEL011202	4899.99	-0.64138	0.202483	-3.16755	0.001537	0.034052
AAEL004576	55.46567	-0.94456	0.303401	-3.11325	0.00185	0.038634
AAEL011416	20.50216	-0.91963	0.296211	-3.10465	0.001905	0.039098
AAEL000305	69.42238	-0.82838	0.269374	-3.0752	0.002104	0.041984
AAEL011499	19.02131	-0.90922	0.298348	-3.04753	0.002307	0.044536
AAEL024830	179.1432	-0.71887	0.237273	-3.02971	0.002448	0.046411
AAEL021576	21.2682	-0.76916	0.255667	-3.00844	0.002626	0.048769
AAEL008183	1134.361	-0.60889	0.20306	-2.99857	0.002712	0.049542

FC: fold change; P-adj: adjusted *p* value

7 dpi 32 °C upregulated

GeneID	Base mean	log2(FC)	StdErr	Wald-Stats	P-value	P-adj
AAEL017976	446.7218	2.919349	0.359109	8.129415	4.31E-16	5.31E-12
AAEL027610	342.6254	2.880902	0.363423	7.927137	2.24E-15	1.38E-11
AAEL006323	204.7075	1.811199	0.261353	6.930083	4.21E-12	1.72E-08
AAEL017975	659.6938	2.424532	0.362353	6.691067	2.22E-11	6.81E-08
AAEL006990	710.9443	1.738503	0.265983	6.536144	6.31E-11	1.55E-07
AAEL020330	80.5189	2.272107	0.36694	6.192037	5.94E-10	1.04E-06
AAEL023591	3057.906	1.555017	0.250685	6.20308	5.54E-10	1.04E-06
AAEL001098	139.8757	1.82806	0.299515	6.103404	1.04E-09	1.42E-06
AAEL026008	3314.774	1.576086	0.257949	6.110066	9.96E-10	1.42E-06
AAEL027829	142.1775	2.10786	0.354259	5.95006	2.68E-09	3.30E-06
AAEL014348	162.4059	1.266885	0.217238	5.831786	5.48E-09	5.88E-06
AAEL001646	97.74182	1.763908	0.302848	5.824402	5.73E-09	5.88E-06
AAEL001077	180.9688	1.285756	0.224488	5.727507	1.02E-08	9.64E-06
AAEL014020	160.3409	2.096797	0.366975	5.713736	1.11E-08	9.71E-06
AAEL013350	177.675	2.077968	0.367169	5.659434	1.52E-08	1.25E-05
AAEL003345	9199.334	1.887363	0.336661	5.606128	2.07E-08	1.59E-05
AAEL005985	4343.619	0.649724	0.116935	5.556259	2.76E-08	1.78E-05
AAEL009127	823.6629	1.67233	0.300912	5.557547	2.74E-08	1.78E-05
AAEL000037	232.6124	1.394313	0.250482	5.566527	2.60E-08	1.78E-05
AAEL003728	266.4348	1.480512	0.269575	5.492032	3.97E-08	2.44E-05
AAEL011203	68.67572	1.344507	0.247184	5.439291	5.35E-08	3.13E-05
AAEL023844	316.0924	1.360717	0.250813	5.425223	5.79E-08	3.24E-05
AAEL003505	1961.892	1.117491	0.209226	5.341064	9.24E-08	4.94E-05
AAEL013770	116.996	1.559145	0.294503	5.294154	1.20E-07	6.13E-05
AAEL008473	3062.51	1.718822	0.32574	5.276673	1.32E-07	6.47E-05
AAEL022208	91.06876	1.446752	0.274668	5.267276	1.38E-07	6.55E-05
AAEL019751	776.5939	1.287111	0.250391	5.140404	2.74E-07	0.00012
AAEL022059	87.8943	1.848594	0.367529	5.029791	4.91E-07	0.000208
AAEL023999	19.01198	1.421984	0.285488	4.980893	6.33E-07	0.00026
AAEL001087	547.9228	1.656446	0.333591	4.965506	6.85E-07	0.000272
AAEL006883	538.1236	1.584296	0.320162	4.948414	7.48E-07	0.000288
AAEL002610	1194.176	1.528829	0.309799	4.934908	8.02E-07	0.000299
AAEL014045	379.1003	1.57201	0.320175	4.909841	9.12E-07	0.00033
AAEL022253	340.4038	1.800957	0.367743	4.89732	9.72E-07	0.000341
AAEL022334	1089.324	1.280693	0.262337	4.881862	1.05E-06	0.000359
AAEL000825	20.46806	1.741355	0.35981	4.839651	1.30E-06	0.000421
AAEL012764	1407.784	1.42573	0.29495	4.833795	1.34E-06	0.000423
AAEL022079	96.07221	1.36087	0.284021	4.791448	1.66E-06	0.000497
AAEL013284	280.9838	1.748619	0.367739	4.755048	1.98E-06	0.000581
AAEL001594	91.54731	1.528298	0.32188	4.74804	2.05E-06	0.000588
AAEL009850	110.7414	1.001692	0.212355	4.717067	2.39E-06	0.000654
AAEL009198	828.4534	0.74214	0.15723	4.720089	2.36E-06	0.000654

GeneID	Base mean	log2(FC)	StdErr	Wald-Stats	P-value	P-adj
AAEL008274	1180.8	0.943283	0.200634	4.701506	2.58E-06	0.000691
AAEL011038	590.2102	0.995029	0.211846	4.696952	2.64E-06	0.000691
AAEL001084	129.3413	1.432871	0.308204	4.649092	3.33E-06	0.000837
AAEL010434	58.39876	1.395248	0.300437	4.644068	3.42E-06	0.00084
AAEL003626	316.58	1.332189	0.287698	4.630514	3.65E-06	0.00088
AAEL019504	1039.776	1.410592	0.304987	4.625083	3.74E-06	0.000886
AAEL017334	3153.811	1.621586	0.352744	4.597059	4.28E-06	0.000995
AAEL001813	234.0416	1.543163	0.336516	4.585708	4.52E-06	0.001012
AAEL021302	754.1271	1.259606	0.274511	4.58855	4.46E-06	0.001012
AAEL025126	82.35019	1.683724	0.36779	4.577951	4.70E-06	0.001031
AAEL014510	176.4301	0.875211	0.19136	4.573642	4.79E-06	0.001034
AAEL007765	3748.655	0.617202	0.136073	4.535817	5.74E-06	0.001217
AAEL001949	67.29029	0.96705	0.214315	4.51229	6.41E-06	0.001315
AAEL013812	464.8684	1.220807	0.27322	4.468219	7.89E-06	0.001591
AAEL005045	230.6594	1.621136	0.363703	4.457303	8.30E-06	0.001621
AAEL000044	884.8495	1.444323	0.323811	4.460381	8.18E-06	0.001621
AAEL001241	44.46046	1.263618	0.284338	4.444064	8.83E-06	0.001688
AAEL012712	243.566	1.454281	0.330154	4.404857	1.06E-05	0.001943
AAEL006581	168.3474	1.086651	0.246615	4.406262	1.05E-05	0.001943
AAEL007218	307.9408	0.998626	0.227978	4.380358	1.18E-05	0.002143
AAEL024179	3549.131	0.830567	0.190646	4.356583	1.32E-05	0.002355
AAEL019590	200.524	1.329421	0.306152	4.342351	1.41E-05	0.002453
AAEL010393	19.65437	1.556713	0.358575	4.341384	1.42E-05	0.002453
AAEL010076	208.0212	1.270966	0.293128	4.335867	1.45E-05	0.00248
AAEL013345	35.93927	1.583963	0.367557	4.309437	1.64E-05	0.002732
AAEL010379	577.0775	1.267505	0.295651	4.287161	1.81E-05	0.002948
AAEL006805	1722.145	1.145897	0.267372	4.28577	1.82E-05	0.002948
AAEL007090	226.3581	1.407091	0.329313	4.272814	1.93E-05	0.003084
AAEL007902	293.3958	1.083611	0.253791	4.269701	1.96E-05	0.003087
AAEL001293	335.6991	1.421436	0.334346	4.251392	2.12E-05	0.003308
AAEL013346	44.75176	1.523031	0.360531	4.224415	2.40E-05	0.003638
AAEL008622	121.9277	1.508754	0.357862	4.216025	2.49E-05	0.00373
AAEL017345	1297.581	1.064248	0.252898	4.208207	2.57E-05	0.003815
AAEL003954	557.3156	1.458347	0.347417	4.197683	2.70E-05	0.003949
AAEL007387	919.4093	0.662786	0.158004	4.194744	2.73E-05	0.003953
AAEL007126	180.1804	1.368633	0.326626	4.190219	2.79E-05	0.003986
AAEL027654	931.9174	1.162701	0.278422	4.17604	2.97E-05	0.004194
AAEL026537	18.50074	1.429866	0.342787	4.171298	3.03E-05	0.004234
AAEL003708	104.9343	1.061444	0.25543	4.155524	3.25E-05	0.004436
AAEL002917	824.9129	1.358663	0.328008	4.142165	3.44E-05	0.004541
AAEL015465	195.1952	1.123156	0.271441	4.137748	3.51E-05	0.004541
AAEL014556	983.993	1.218744	0.294542	4.137754	3.51E-05	0.004541
AAEL017380	38.84343	1.523205	0.367482	4.144982	3.40E-05	0.004541
AAEL006904	1600.12	0.837621	0.203669	4.112657	3.91E-05	0.004904
AAEL014539	51.32586	1.348928	0.32816	4.11058	3.95E-05	0.004904

GeneID	Base mean	log2(FC)	StdErr	Wald-Stats	P-value	P-adj
AAEL005533	90.23157	1.49545	0.364803	4.099338	4.14E-05	0.005054
AAEL002658	301.2503	0.950003	0.231764	4.099005	4.15E-05	0.005054
AAEL010337	337.9218	1.240084	0.303146	4.090715	4.30E-05	0.005136
AAEL008635	1277.263	1.398547	0.343442	4.072155	4.66E-05	0.005457
AAEL008532	72.24792	1.497715	0.367772	4.072407	4.65E-05	0.005457
AAEL010206	397.5056	1.162759	0.285702	4.069832	4.70E-05	0.00546
AAEL014246	3290.577	1.202206	0.29701	4.047694	5.17E-05	0.005891
AAEL010769	538.2967	1.162549	0.287645	4.041606	5.31E-05	0.005991
AAEL007989	7.168395	1.440137	0.359007	4.011445	6.03E-05	0.006724
AAEL000811	360.2371	1.207735	0.301169	4.01016	6.07E-05	0.006724
AAEL021929	175.7643	1.46841	0.3671	4.000025	6.33E-05	0.006956
AAEL014604	162.4537	0.975968	0.245487	3.975643	7.02E-05	0.007574
AAEL003656	271.4358	0.949151	0.239128	3.96922	7.21E-05	0.007713
AAEL004118	662.4558	1.139949	0.289203	3.94169	8.09E-05	0.00858
AAEL012457	618.3535	1.328268	0.338319	3.926079	8.63E-05	0.008925
AAEL022982	610.0555	0.997812	0.254062	3.927434	8.59E-05	0.008925
AAEL014335	516.5232	0.944089	0.24138	3.911209	9.18E-05	0.009414
AAEL012856	783.5518	1.389763	0.356661	3.896596	9.76E-05	0.009917
AAEL026161	396.1913	1.190966	0.306088	3.89092	9.99E-05	0.010004
AAEL013163	994.9419	0.912059	0.236521	3.856147	0.000115	0.011245
AAEL014419	138.4878	1.189442	0.310149	3.83507	0.000126	0.012063
AAEL002301	595.0697	1.08825	0.283741	3.835368	0.000125	0.012063
AAEL027362	23.11579	1.342468	0.350727	3.827672	0.000129	0.012335
AAEL008401	27.55802	1.396174	0.365882	3.81591	0.000136	0.012839
AAEL006321	2850.859	0.650008	0.17071	3.807685	0.00014	0.013172
AAEL001667	530.0897	1.233206	0.325737	3.785899	0.000153	0.014059
AAEL013257	44.33485	0.882617	0.234425	3.765024	0.000167	0.015063
AAEL014541	277.04	1.183489	0.314746	3.760144	0.00017	0.015247
AAEL003632	22.32452	1.236254	0.328957	3.758101	0.000171	0.015261
AAEL012640	86.26759	1.362207	0.364137	3.740917	0.000183	0.016226
AAEL000915	292.4677	1.284868	0.344692	3.727585	0.000193	0.016747
AAEL023509	149.1324	1.001252	0.270994	3.694743	0.00022	0.018803
AAEL001403	60.93914	0.922478	0.249908	3.691266	0.000223	0.018869
AAEL011350	1004.095	0.927922	0.251447	3.690335	0.000224	0.018869
AAEL024560	145.6707	0.83561	0.227987	3.665167	0.000247	0.020544
AAEL010917	44.85952	1.332682	0.364534	3.655852	0.000256	0.021021
AAEL002124	763.5451	0.95074	0.259965	3.657187	0.000255	0.021021
AAEL007453	25.62713	1.318314	0.362529	3.636434	0.000276	0.022424
AAEL007784	158.5976	1.330548	0.367271	3.622796	0.000291	0.023129
AAEL004844	11.5029	1.234893	0.341244	3.618792	0.000296	0.023339
AAEL002075	38.14323	1.281871	0.355178	3.609097	0.000307	0.024074
AAEL010480	226.9322	1.035451	0.287839	3.597329	0.000322	0.02503
AAEL010068	1383.658	1.106547	0.307802	3.59499	0.000324	0.025097
AAEL013525	617.8744	0.749726	0.208717	3.592064	0.000328	0.025222
AAEL027157	337.3348	0.878586	0.244974	3.586445	0.000335	0.025612

GeneID	Base mean	log2(FC)	StdErr	Wald-Stats	P-value	P-adj
AAEL013159	28.46296	1.166178	0.325735	3.58014	0.000343	0.026076
AAEL020502	325.3333	0.91947	0.257584	3.569592	0.000358	0.026817
AAEL025574	808.3258	0.985478	0.276021	3.570302	0.000357	0.026817
AAEL007789	132.4106	0.786266	0.220558	3.564895	0.000364	0.027137
AAEL026819	109.2034	0.922467	0.258952	3.562312	0.000368	0.0272
AAEL009629	3513.993	1.096565	0.307927	3.561119	0.000369	0.0272
AAEL001420	7877.547	0.939145	0.264278	3.553619	0.00038	0.027821
AAEL012701	2446.635	0.589274	0.165915	3.551665	0.000383	0.027863
AAEL019641	592.2827	0.932353	0.263518	3.538094	0.000403	0.029163
AAEL003123	1780.523	1.220832	0.345505	3.53347	0.00041	0.029504
AAEL003713	245.679	0.861885	0.245095	3.516527	0.000437	0.03127
AAEL009640	143.485	0.798098	0.227962	3.501016	0.000463	0.032766
AAEL013885	6407.882	1.186245	0.338801	3.5013	0.000463	0.032766
AAEL017071	967.1173	0.94955	0.273901	3.46676	0.000527	0.036609
AAEL022589	70.16731	1.267022	0.367725	3.44557	0.00057	0.037802
AAEL013109	209.294	0.958501	0.27825	3.444748	0.000572	0.037802
AAEL003076	953.9536	1.051074	0.304708	3.449449	0.000562	0.037802
AAEL007773	438.2615	1.034985	0.300092	3.448887	0.000563	0.037802
AAEL019602	251.8779	0.835476	0.242534	3.444778	0.000572	0.037802
AAEL001794	1266.131	1.029609	0.298217	3.452549	0.000555	0.037802
AAEL002555	176.1723	1.030978	0.299896	3.437786	0.000586	0.038375
AAEL018103	407.3152	1.138742	0.331503	3.435089	0.000592	0.038554
AAEL008628	10.12739	1.241043	0.362826	3.42049	0.000625	0.040469
AAEL009645	6653.894	0.856867	0.251118	3.412206	0.000644	0.040896
AAEL002347	111.4024	1.241881	0.363978	3.411964	0.000645	0.040896
AAEL026496	5.767867	1.193677	0.349698	3.413448	0.000641	0.040896
AAEL013347	949.1318	1.039507	0.305001	3.408204	0.000654	0.04122
AAEL005221	3658.496	0.913722	0.268189	3.407011	0.000657	0.04122
AAEL014891	165.9633	1.144306	0.337079	3.394769	0.000687	0.042672
AAEL002432	12.90657	1.221794	0.361287	3.381785	0.00072	0.04409
AAEL010881	80.62625	0.828413	0.244971	3.381683	0.00072	0.04409
AAEL004003	23.51713	1.11224	0.328874	3.38196	0.00072	0.04409
AAEL005769	364.4213	1.109089	0.328161	3.379705	0.000726	0.044188
AAEL028247	23.29229	1.227407	0.36364	3.375335	0.000737	0.044675
AAEL019564	620.3279	1.028056	0.306753	3.351409	0.000804	0.047779
AAEL026390	1302.221	0.608807	0.182065	3.343893	0.000826	0.048856
AAEL013341	940.2407	0.80422	0.241003	3.336967	0.000847	0.049613
AAEL018159	583.4205	0.966312	0.28956	3.337169	0.000846	0.049613

FC: fold change; P-adj: adjusted *p* value

7 dpi 32 °C downregulated

GeneID	Base mean	log2(FC)	StdErr	Wald-Stats	P-value	P-adj
AAEL024449	7.900429	-1.89165	0.366118	-5.16676	2.38E-07	0.000109
AAEL006938	25.42905	-1.65673	0.341677	-4.84882	1.24E-06	0.000413
AAEL020913	93.79685	-0.84025	0.174434	-4.81699	1.46E-06	0.000448
AAEL018041	131.1753	-1.70489	0.363607	-4.68883	2.75E-06	0.000704
AAEL021011	89.28689	-1.15135	0.267228	-4.30848	1.64E-05	0.002732
AAEL020306	4.225345	-1.54521	0.365169	-4.23148	2.32E-05	0.00357
AAEL000322	434.8694	-0.81085	0.194666	-4.16534	3.11E-05	0.004297
AAEL000035	22.7378	-1.51775	0.365904	-4.14795	3.35E-05	0.004535
AAEL024183	54.88617	-1.51972	0.367732	-4.13267	3.59E-05	0.004547
AAEL006871	390.1212	-0.66076	0.159871	-4.13311	3.58E-05	0.004547
AAEL013003	347.7431	-1.09056	0.266392	-4.09382	4.24E-05	0.005117
AAEL005621	34.90218	-1.49456	0.367646	-4.0652	4.80E-05	0.005517
AAEL004843	822.7724	-0.70063	0.175736	-3.98683	6.70E-05	0.007289
AAEL005070	840.8001	-0.81728	0.21007	-3.89051	0.0001	0.010004
AAEL023039	27.23422	-1.28358	0.332377	-3.86183	0.000113	0.011164
AAEL005068	942.282	-1.11388	0.293884	-3.79021	0.000151	0.014027
AAEL026029	11.85499	-1.27482	0.346728	-3.67673	0.000236	0.019769
AAEL022225	19.50603	-1.26525	0.347996	-3.63582	0.000277	0.022424
AAEL008435	746.1219	-0.9798	0.269878	-3.63054	0.000283	0.022739
AAEL019689	202.5493	-1.1764	0.337385	-3.48682	0.000489	0.034359
AAEL025193	13.09782	-1.23745	0.35752	-3.46122	0.000538	0.037161
AAEL025401	534.8338	-0.78726	0.228847	-3.44011	0.000581	0.038251
AAEL026468	471.9231	-0.68725	0.204249	-3.36477	0.000766	0.046194
AAEL018184	46.58388	-1.22761	0.365171	-3.36172	0.000775	0.046478

FC: fold change; P-adj: adjusted *p* value

Supplementary Table 3. Number of DEGs.

		**Upregulated genes** P-adj<0.05, FC>+1.5		**Downregulated genes** P-adj<0.05, FC>-1.5	
		Total	Immune	Total	Immune
**3 dpi**	18ºC	141	12	134	4
	28ºC	374	39	36	1
	32ºC	26	2	4	4
**7 dpi**	18ºC	100	3	13	3
	28ºC	381	41	38	0
	32ºC	170	18	24	0
**Total**		1192	115	249	12

Adj: adjusted; FC: fold change

Supplementary Table 4. Classical and non-classical immune gene families and number of genes identified.

Category	Gene family	Number of genes identified
**Classical immune families**	Anti-microbial peptides	12
	Apoptosis	29
	Autophagy	21
	Catalase	1
	CLIP	92
	C-type lectins (CTL)	48
	Fibrinogen-related protein (FREP)	31
	Galectin	10
	Gram-negative binding proteins (GNBP)	7
	IMD pathway	21
	JAK-STAT pathway	6
	Leucine-rich repeat-containing proteins/ Leucine-rich repeat immune proteins (LRR/ LRIMs)	56
	Lysozymes	5
	Myeloid differentiation 2-related lipid recognition protein (ML)	22
	Peroxidase	21
	Peptidoglycan recognition proteins (PGRP)	9
	Prophenoloxidase (PPO)	10
	Relish	3
	Scavenger receptors (SCR)	20
	Serpins	25
	RNA inhibition pathway	30
	Superoxide dismutase (SOD)	8
	Spatzle	7
	Thioester proteins (TEP)	11
	Toll pathway	16
**Non-classical immune families**	Trypsin	59
	Serine proteases	119

Tubulin	21
Actin	31
Myosin	23
Lachesin	6
Heat shock protein	12
Cytochrome P450	150
Lethal (2) essential for life protein, l2efl	10
Vacuolar ATPase	14
Sidestep proteins	6
Salivary proteins	26
**Total**	**998**

Supplementary Table 5. Classical immune genes.

	Gene ID	Name/Description
**Classical**		
**AMP**	AAEL003389	ATT
	AAEL029038	CECA
	AAEL029046	CECD
	AAEL029044	CECE
	AAEL029041	CECD
	AAEL029047	CECN
	AAEL027792	DEFA
	AAEL003832	DEFC
	AAEL003857	DEFD
	AAEL004833	DPT1
	AAEL004522	GAM1
	AAEL017536	Holotricin glycine rich repeat protein (GRRP) anti-microbial peptide
**Autophagy**	AAEL009089	APG12
	AAEL019779	APG16L
	AAEL013063	APG18A
	AAEL013995	APG18B
	AAEL000955	APG3
	AAEL010516	APG4A
	AAEL007228	APG4B
	AAEL002286	APG5
	AAEL010427	APG6
	AAEL010641	APG7A
	AAEL012306	APG7B
	AAEL007162	APG8
	AAEL009105	APG9
	AAEL001515	DEBCL
	AAEL003777	APG2
	AAEL020638	TOR
	AAEL009814	Autophagy related gene
	AAEL010791	Autophagy-specific protein, putative
	AAEL021581	BUFFY
	AAEL019922	APG1
	AAEL021061	APG10
**GNBP**	AAEL000652	GNBPA2
	AAEL003889	GNBPB1
	AAEL003894	GNBPB5
	AAEL007064	GNBPB6
	AAEL007626	GNBPA1
	AAEL009176	GNBPB3
	AAEL009178	GNBPB4

	Gene ID	Name/Description
**Caspase and apoptosis**	AAEL014148	Dredd
	AAEL026744	CASPS9
	AAEL011562	Dronc
	AAEL005956	CASPS16
	AAEL005955	CASPS17
	AAEL003439	CASPS18
	AAEL003444	CASPS19
	AAEL017498	CASPS21
	AAEL012143	CASPS7
	AAEL014348	CASPS8
	AAEL000874	ARK
	AAEL004392	IMP
	AAEL014196	Michelob-x
	AAEL011277	Apoptosis stimulating of p53
	AAEL009642	Cathepsin B
	AAEL009637	Cathepsin B
	AAEL000420	Cathepsin O
	AAEL002833	Cathepsin L
	AAEL011167	Cathepsin L
	AAEL006389	Cathepsin L
	AAEL006633	IAP2
	AAEL009074	IAP1
	AAEL007713	Viral IAP-associated factor, putative
	AAEL010486	Viral IAP-associated factor, putative
	AAEL011096	Viral IAP-associated factor, putative
	AAEL012446	IAP6
	AAEL025438	Wengen
	AAEL008634	JNK
	AAEL014251	IAP5
**CLIP**	AAEL002601	CLIPA1
	AAEL019853	CLIP
	AAEL019781	CLIP
	AAEL015430	CLIP
	AAEL026876	CLIP
	AAEL019590	CLIP
	AAEL000224	CLIP
	AAEL026937	CLIP
	AAEL005718	CLIPA3
	AAEL002288	CLIPA4
	AAEL002629	CLIPA6
	AAEL001675	CLIPA10
	AAEL002585	CLIPA11
	AAEL002590	CLIPA12
	AAEL002595	CLIPA14

Gene ID	Name/Description
AAEL002126	CLIPA15
AAEL008404	CLIPA16
AAEL008668	CLIP
AAEL000074	CLIPB1
AAEL005064	CLIPB5
AAEL003243	CLIPB13A
AAEL003253	CLIPB13B
AAEL014349	CLIPB15
AAEL005648	CLIPB16
AAEL007006	CLIPA17
AAEL001084	CLIPB21
AAEL014140	CLIPB24
AAEL014137	CLIPB25
AAEL007993	CLIPB27
AAEL013245	CLIPB28
AAEL006674	CLIPB29
AAEL000760	CLIPB30
AAEL006161	CLIPB31
AAEL000099	CLIPB33
AAEL000028	CLIPB34
AAEL000037	CLIPB35
AAEL000038	CLIPB6-B36
AAEL005431	CLIPB37
AAEL003628	CLIPB38
AAEL003632	CLIPB39
AAEL003631	CLIPB41
AAEL006168	CLIPB42
AAEL014354	CLIPB43
AAEL005060	CLIPB44
AAEL001077	CLIPB45
AAEL005093	CLIPB46
AAEL027429	CLIPB76
AAEL014139	CLIPB79
AAEL017003	Clip-domain serine protease, family B
AAEL011991	CLIPC1
AAEL007593	CLIPC2
AAEL007597	CLIPC3
AAEL004524	CLIPC5B
AAEL011593	CLIPC11
AAEL012711	CLIPC12
AAEL012712	CLIPC13
AAEL004948	CLIPC14
AAEL010270	CLIPC15
AAEL012713	CLIPC16
AAEL007796	CLIPD1

	Gene ID	Name/Description
	AAEL004979	CLIPD2
	AAEL002997	CLIPD3
	AAEL002124	CLIPD6
	AAEL005906	CLIPD8
	AAEL000238	CLIPD9
	AAEL015109	CLIPD10
	AAEL011375	CLIPD11
	AAEL005792	CLIPE8
	AAEL001233	CLIPE9
	AAEL010773	CLIPE10
	AAEL018347	CLIPE12
	AAEL019767	SP-CLIP-SP
	AAEL006689	CUBSP1
	AAEL006696	CUBSP2
	AAEL006700	CUBSP3
	AAEL006703	CUBSP4
	AAEL016975	ZFSP
	AAEL023229	HP14
	AAEL005748	CBSP
	AAEL027371	SPH145
	AAEL013413	IgSP2
	AAEL014367	SEASP
	AAEL011349	SP55/SP218
	AAEL001098	CLIP-domain serine protease, putative
	AAEL003279	CLIP-domain serine protease, putative
	AAEL006576	CLIP-domain serine protease, putative
	AAEL009722	CLIP-domain serine protease, putative
	AAEL014386	CLIP-domain serine protease, putative
	AAEL015465	CLIP-domain serine protease, putative
	AAEL007587	CLIP-domain serine protease
	AAEL022578	CLIP-domain serine protease
	AAEL015637	Aaeg:CLIP23
**CTL**	AAEL000283	CTLMA16
	AAEL029028	CTL
	AAEL029020	CTL
	AAEL029053	CTL
	AAEL029039	CTL
	AAEL009338	CTL10
	AAEL000533	CTL16
	AAEL000543	CTLMA11
	AAEL000556	CTL25
	AAEL000563	CTLMA15
	AAEL002524	CTL24
	AAEL022136	CTL6
	AAEL005482	CTL18

	Gene ID	Name/Description
	AAEL005641	CTLGA5
	AAEL008299	CTL11
	AAEL008681	CTL12
	AAEL021200	CTLSE1
	AAEL009209	CTLGA6
	AAEL018207	CTL8
	AAEL011070	CTLGA3
	AAEL011078	CTLGA1
	AAEL011079	CTLMA10
	AAEL029068	CTL5
	AAEL011404	CTL19
	AAEL011407	CTL20
	AAEL011408	CTL21
	AAEL011453	CTL14
	AAEL011455	CTLMA12
	AAEL011612	CTLMA6
	AAEL011621	CTLMA13
	AAEL012353	CTL15
	AAEL018265	CTL9
	AAEL013853	CTLGA2
	AAEL014382	CTLMA14
	AAEL019633	CTLGA9
	AAEL025802	CTLD-S
	AAEL025598	CTLD-S
	AAEL022823	mosGCTL-11
	AAEL026955	CTLD-S
	AAEL023353	CTLD-S
	AAEL014384	CTLD-S
	AAEL027215	CTLD-S
	AAEL027443	CTLD-S
	AAEL011622	CLSP1(mosGCTL-31)
	AAEL006825	CTLD-E
	AAEL014357	CTLD-X
	AAEL006958	CTLD-X
	AAEL001935	CTL-like protein 1
**FREP**	AAEL000508	FREP15
	AAEL000726	FREP20
	AAEL000749	FREP22
	AAEL001713	FREP2
	AAEL002713	FREP31
	AAEL003156	FREP28
	AAEL003294	FREP3
	AAEL006691	FREP
	AAEL006699	FREP34
	AAEL006702	FREP33

	Gene ID	Name/Description
	AAEL006704	FREP18
	AAEL007942	FREP14
	AAEL008104	FREB23
	AAEL009384	FREP5
	AAEL009723	FREP11
	AAEL010117	FREP35
	AAEL011009	FREP13
	AAEL011633	FREP16
	AAEL011634	FREP12
	AAEL013417	FREP24
	AAEL013506	FREP29
	AAEL014432	FREP25
	AAEL021180	FREP26
	AAEL025451	FREP27
	AAEL014773	FREP
	AAEL019868	FREP
	AAEL020192	FREP
	AAEL023956	FREP
	AAEL025744	FREP
	AAEL028175	FREP
	AAEL011007	FREP
**Galectin**	AAEL003541	GALE1
	AAEL003840	GALE11
	AAEL003844	GALE5
	AAEL004196	GALE3
	AAEL005293	GALE8A
	AAEL009842	GALE12
	AAEL009850	GALE14
	AAEL012003	GALE6B
	AAEL012135	GALE2
	AAEL026564	Galectin
**IMD pathway**	AAEL010083	Imd
	AAEL027860	Caspar
	AAEL001932	FADD
	AAEL012510	IKK2
	AAEL018130	TAK1
	AAEL003245	IKK1
	AAEL026170	TAB2
	AAEL003371	Beta-TrCP [KO:K03362]
	AAEL003103	Ubiquitin-conjugating enzyme E2-17 kDa [KO:K06689] [EC:2.3.2.23]
	AAEL011873	Ubiquitin-conjugating enzyme E2 variant 1 [KO:K10704]
	AAEL002118	Ubiquitin-conjugating enzyme E2 N [KO:K10580] [EC:2.3.2.23]
	AAEL028161	Mitogen-activated protein kinase kinase kinase 4 isoform X1 [KO:K04428] [EC:2.7.11.25]

	Gene ID	Name/Description
	AAEL023782	Mitogen-activated protein kinase kinase kinase 4 isoform X1 [KO:K04428] [EC:2.7.11.25]
	AAEL001622	Dual specificity mitogen-activated protein kinase kinase 3 isoform X1 [KO:K04432] [EC:2.7.12.2]
	AAEL008379	Mitogen-activated protein kinase 14B isoform X2 [KO:K04441] [EC:2.7.11.24]
	AAEL013261	Cyclic AMP-dependent transcription factor ATF-2 [KO:K04450]
	AAEL021333	Dual specificity mitogen-activated protein kinase kinase hemipterous isoform X1 [KO:K04431] [EC:2.7.12.2]
	AAEL003505	Transcription factor AP-1 isoform X2 [KO:K04448]
	AAEL008953	Transcription factor kayak isoform X3 [KO:K09031]
	AAEL011563	Ankyrin-3 isoform X1 [KO:K10380]
	AAEL013466	Ankyrin-3 isoform X1 [KO:K10380]
**Jak-stat**	AAEL012471	DOME
	AAEL012553	HOP
	AAEL020559	STAT
	AAEL019728	SOCS
	AAEL009822	GPRMGL5
	AAEL009645	Hypotheical protein
**Lysozymes**	AAEL003712	LYSC10
	AAEL003723	LYSC11
	AAEL019435	LYSC6
	AAEL015404	LYSC7B
	AAEL017132	LYSC4
**LRR/LRIM**	AAEL010125	LRIM17
	AAEL010132	LRIM3
	AAEL012255	LRIM13
	AAEL001420	LRIM8
	AAEL001401	LRIM10A
	AAEL012092	Leucine-rich repeat
	AAEL010772	Leucine-rich repeat-containing protein
	AAEL012093	Leucine-rich transmembrane protein
	AAEL005734	Leucine-rich transmembrane protein
	AAEL002295	Leucine-rich transmembrane protein
	AAEL003597	Leucine-rich transmembrane protein
	AAEL000243	Leucine-rich transmembrane protein
	AAEL006026	Leucine rich protein, putative
	AAEL007565	Leucine rich protein, putative
	AAEL003554	Leucine rich repeat protein
	AAEL004711	Testis specific leucine rich repeat protein
	AAEL004466	Leucine-rich immune protein (Coil-less)
	AAEL002615	Leucine-rich transmembrane protein
	AAEL006377	LRIM31
	AAEL007785	Leucine-rich transmembrane protein
	AAEL006920	LRIM20
	AAEL012763	LRIM24

	Gene ID	Name/Description
	AAEL000762	LRIM19
	AAEL007224	LRIM22
	AAEL001402	LRIM10B
	AAEL001417	LRIM7
	AAEL009792	LRIM25
	AAEL007103	LRIM15
	AAEL010128	LRIM4
	AAEL007778	Leucine-rich transmembrane protein
	AAEL012086	LRIM1
	AAEL012538	LRIM6
	AAEL008658	LRIM16
	AAEL010656	LRIM12
	AAEL001649	Leucine aminopeptidase
	AAEL007363	Leucine-rich transmembrane protein
	AAEL000108	Leucine aminopeptidase
	AAEL006975	Leucine aminopeptidase
	AAEL000424	Leucine aminopeptidase
	AAEL012767	LRIM5
	AAEL003262	Leucine-rich transmembrane protein
	AAEL004773	Leucine carboxyl methyltransferase
	AAEL006797	F-box/Leucine rich repeat protein
	AAEL001414	LRIM9
	AAEL009894	LRIM21
	AAEL011387	Leucine-rich repeat
	AAEL002307	Leucine-rich transmembrane protein
	AAEL007442	F-box/leucine rich repeat protein
	AAEL010793	F-box/leucine rich repeat protein
	AAEL005762	Leucine-rich transmembrane proteins
	AAEL001766	Leucine-rich transmembrane proteins
	AAEL010286	Leucine-rich transmembrane protein
	AAEL003713	Leucine-rich transmembrane protein
	AAEL003408	Leucine-rich transmembrane protein
	AAEL012911	LRIM18
	AAEL000925	Leucine-zipper-like transcriptional regulator 1 (LZTR-1)
**ML**	AAEL009531	Niemann-Pick
	AAEL004120	ML1
	AAEL006854	ML13
	AAEL009553	ML9B
	AAEL009555	ML15A
	AAEL009556	ML15B
	AAEL015140	ML16
	AAEL009760	ML21
	AAEL026174	ML9B
	AAEL012064	ML2
	AAEL007592	ML20B

	Gene ID	Name/Description
	AAEL007591	ML26A
	AAEL001654	ML30
	AAEL019611	ML31
	AAEL001634	ML32
	AAEL001650	ML33
	AAEL015136	ML6
	AAEL015137	ML20
	AAEL015139	ML22A
	AAEL008492	DVRF1
	AAEL019883	ML1
	AAEL009956	Aaeg:ML18
**PGRP**	AAEL017056	PGRPS4
	AAEL019745	PGPPLD putative
	AAEL009474	PGRPS1
	AAEL010171	PGRPLB
	AAEL012380	PGRPLA
	AAEL027982	PGRPLE
	AAEL014640	PGRPLC
	AAEL007039	Peptidoglycan recognition protein (short)
	AAEL021026	PGRP
**ROS**	AAEL000342	PRDX
	AAEL020747	PRDX
	AAEL026038	PRDX
	AAEL004386	pxt
	AAEL000495	GPXH3
	AAEL002309	TPX4
	AAEL002354	HPX5
	AAEL019408	TPX2
	AAEL004388	HPX8A
	AAEL004390	HPX8B
	AAEL006014	HPX1
	AAEL007563	DUOX
	AAEL008397	GPXH2
	AAEL009051	TPX5
	AAEL012069	GPXH1
	AAEL013171	HPX2
	AAEL013528	TPX1
	AAEL025567	TPX3
	AAEL019639	HPX3
	AAEL011941	Oxidase/peroxidase
	AAEL000507	Peroxidase
**PPO**	AAEL011763	PPO3
	AAEL011764	PPO10
	AAEL013492	PPO5
	AAEL013493	PPO7

	Gene ID	Name/Description
	AAEL013496	PPO8
	AAEL014544	PPO1
	AAEL013501	PPO4
	AAEL015113	PPO2
	AAEL015116	PPO1
	AAEL020579	PPO9
**Relish**	AAEL007696	REL1A
	AAEL006930	REL1B
	AAEL007624	REL2
**SCR**	AAEL000227	SCRB8
	AAEL000234	SCRB7
	AAEL000256	SCRB9
	AAEL001914	SCRAC1
	AAEL002741	SCRB6
	AAEL005374	SCRB1
	AAEL005979	SCRB3
	AAEL006355	SCRC1
	AAEL006361	SCRC2
	AAEL008370	SCRB17
	AAEL009192	SCRASP1
	AAEL009420	SCRBQ1
	AAEL009423	SCRBQ2
	AAEL009432	SCRBQ3
	AAEL010655	SCRBSP2
	AAEL011222	SCRB5
	AAEL019436	SCRB16
	AAEL022263	SCRAL1
	AAEL027694	SCRASP3
	AAEL027927	SCR
**SOD**	AAEL004823	MNSOD1
	AAEL005108	MNSOD2
	AAEL006271	CUSOD2
	AAEL019759	CUSOD1
	AAEL019761	CUSOD1
	AAEL019937	SOD-Cu-Zn
	AAEL019938	CUSOD4
	AAEL025388	SOD7
**SPZ**	AAEL001435	SPZ2
	AAEL001929	SPZ5
	AAEL007897	SPZ4
	AAEL008596	SPZ3A
	AAEL012164	SPZ6
	AAEL013433	SPZ1C
	AAEL013434	SPZ1B
**Serpins**	AAEL002699	SRPN7

	Gene ID	Name/Description
	AAEL002704	SRPN
	AAEL002720	SRPN20
	AAEL002730	SRPN21
	AAEL002731	SRPN14
	AAEL003182	SRPN26
	AAEL003653	SRPN12
	AAEL003697	SRPN17
	AAEL005665	SRPN3
	AAEL006137	SRPN19
	AAEL007420	SRPN25
	AAEL007765	SRPN10
	AAEL008364	SRPN9
	AAEL010769	SRPN6
	AAEL011777	SRPN8
	AAEL012378	SRPN
	AAEL013936	SRPN4
	AAEL014078	SRPN2
	AAEL014079	SRPN1
	AAEL014138	SRPN16
	AAEL014141	SRPN5
	AAEL017249	SRPN24
	AAEL020823	SRPN11
	AAEL024006	SRPN
	AAEL028034	SRPN
**siRNA**	AAEL000293	TSN
	AAEL001317	RM62A
	AAEL001612	DCR1
	AAEL001769	RM62B
	AAEL002083	RM62C
	AAEL002351	RM62D
	AAEL004978	RM62E
	AAEL006287	PIWI7
	AAEL006794	DCR2
	AAEL007698	PIWI4
	AAEL007823	PIWI
	AAEL008073	VIG
	AAEL008098	PIWI2
	AAEL008592	DROSHA
	AAEL008687	LOQS
	AAEL008738	RM62F
	AAEL009326	FMR1
	AAEL010402	RM62G
	AAEL010787	DEAD box ATP-dependent RNA helicase
	AAEL011663	Aa-ago2
	AAEL011753	R2D2

	Gene ID	Name/Description
	AAEL012410	AGO1B
	AAEL013233	PIWI5
	AAEL013277	PIWI6
	AAEL013692	PIWI3
	AAEL013985	RM62I
	AAEL023716	SPNE
	AAEL017251	AGO2
	AAEL019460	Combined Dicer-1 and ARM
	AAEL021519	PASHA
**TEP**	AAEL000087	TEP3
	AAEL001794	TEP5
	AAEL004725	TEP
	AAEL005432	TEP
	AAEL008607	TEP
	AAEL009266	c4b-binding protein beta chain
	AAEL012267	TEP1
	AAEL021904	TEP
	AAEL001163	TEP
	AAEL017023	TEP
	AAEL025334	TEP
**Toll**	AAEL000057	TOLL5B
	AAEL000633	TOLL8
	AAEL000671	TOLL6
	AAEL000709	CACT
	AAEL002583	TOLL7
	AAEL004000	TOLL10
	AAEL005075	Ecsit
	AAEL006571	PELLE
	AAEL007619	TOLL5A
	AAEL007642	TUBE
	AAEL007768	MYD
	AAEL009551	TOLL11
	AAEL013441	TOLL9A
	AAEL015018	Toll
	AAEL026297	TOLL1A
	AAEL028236	TRAF6

Supplementary Table 6. Non-classical immune genes.

Gene family	Gene ID	Name/Description
**Trypsin**	AAEL009680	Chymotrypsin
	AAEL001693	Female-specific chymotrypsin
	AAEL006414	Trypsin
	AAEL006430	Trypsin
	AAEL006429	Trypsin
	AAEL010202	Trypsin
	AAEL013703	Trypsin
	AAEL006425	Trypsin
	AAEL013715	Trypsin
	AAEL007601	Trypsin
	AAEL010196	Trypsin
	AAEL013623	Trypsin
	AAEL007992	Trypsin
	AAEL007818	Trypsin 3A1 Precursor
	AAEL013712	Trypsin 5G1 Precursor
	AAEL008079	Trypsin-alpha
	AAEL006403	Trypsin-beta
	AAEL005596	Trypsin-epsilon
	AAEL008080	Trypsin-eta
	AAEL013628	Trypsin-eta
	AAEL005604	Trypsin-epsilon, putative
	AAEL006365	Trypsin-alpha, putative
	AAEL006368	Trypsin-beta, putative
	AAEL006382	Trypsin-eta, putative
	AAEL008097	Trypsin-eta, putative
	AAEL011230	Chymotrypsin, putative
	AAEL011882	Trypsin-zeta, putative
	AAEL006418	Trypsin
	AAEL006903	Trypsin
	AAEL015638	Trypsin
	AAEL000203	Trypsin
	AAEL011553	Trypsin
	AAEL012852	Trypsin
	AAEL004996	Trypsin
	AAEL005609	Trypsin
	AAEL005607	Trypsin
	AAEL005616	Trypsin
	AAEL005611	Trypsin
	AAEL013713	Trypsin
	AAEL006421	Trypsin
	AAEL006378	Trypsin
	AAEL006376	Trypsin

Gene family	Gene ID	Name/Description
	AAEL004543	Trypsin
	AAEL002273	Trypsin
	AAEL014579	Trypsin
	AAEL007600	Trypsin
	AAEL008214	Trypsin
	AAEL006123	Trypsin
	AAEL015432	Trypsin
	AAEL006384	Trypsin
	AAEL007102	Trypsin
	AAEL007602	Trypsin
	AAEL003308	Trypsin
	AAEL006121	Trypsin
	AAEL009853	Trypsin
	AAEL017403	TMOF
	AAEL013284	LT1
**Tubulin**	AAEL004172	Tubulin alpha chain
	AAEL006642	Tubulin alpha chain
	AAEL002848	Tubulin beta chain
	AAEL013229	Tubulin alpha chain
	AAEL019894	Tubulin beta chain
	AAEL006179	Tubulin alpha chain
	AAEL012101	Tubulin alpha chain
	AAEL004939	Tubulin beta chain
	AAEL012843	Tubulin gamma chain
	AAEL005084	Tubulin beta chain
	AAEL002851	Tubulin beta chain
	AAEL005052	Tubulin beta chain
	AAEL012424	Tubulin alpha chain
	AAEL002135	Tubulin-specific chaperone b (tubulin folding cofactor b)
	AAEL003546	Gamma-tubulin complex component 4 (gcp-4)
	AAEL008465	Gamma-tubulin complex component 3 (gcp-3)
	AAEL011067	Tubulin-specific chaperone, putative
	AAEL005303	Beta-tubulin cofactor d
	AAEL013903	Gamma-tubulin complex component 2 (gcp-2)
	AAEL004440	Tubulin-specific chaperone e
	AAEL008679	Alpha-tubulin N-acetyltransferase
**Actin**	AAEL004646	Actin
	AAEL005964	Actin
	AAEL011317	Actin
	AAEL004631	Actin
	AAEL004616	Actin
	AAEL009451	Actin
	AAEL001673	Actin

Gene family	Gene ID	Name/Description
	AAEL005961	Actin
	AAEL003383	Actin
	AAEL012310	Actin
	AAEL004843	Actin
	AAEL011197	Actin
	AAEL011750	Actin
	AAEL001951	Act4
	AAEL003754	Actin binding
	AAEL010664	Actin binding protein, putative
	AAEL013661	Actin binding protein, putative
	AAEL002953	Actin 3 isoform, putative
	AAEL011972	Actin binding protein, putative
	AAEL007660	Suppressor of actin (sac)
	AAEL012519	Actin binding protein, putative
	AAEL008102	Actin binding protein, putative
	AAEL004371	SWI/SNF related matrix associated actin dependent regulator of chromatin subfamily B member 1
	AAEL002822	Arp5
	AAEL002184	F-actin capping protein beta subunit
	AAEL007546	Actin-related protein 2/3 complex subunit 1A
	AAEL001928	Actin-1
	AAEL013778	F-actin capping protein alpha
	AAEL027716	Actin-related protein 2/3 complex subunit 3
	AAEL003701	Actin-binding protein ipp
	AAEL010762	Arp8
**Myosin**	AAEL000596	Myosin
	AAEL012449	Myosin x
	AAEL011905	Myosin i
	AAEL008610	Myosin vii
	AAEL004227	Myosin VI
	AAEL011436	Myosin xv
	AAEL009991	Myosin iii
	AAEL012543	Myosin motor, putative
	AAEL000382	Myosin motor, putative
	AAEL021838	Myosin heavy chain
	AAEL003676	Myosin I homologue, putative
	AAEL007439	Myosin light chain 1,
	AAEL012207	Myosin light chain 1,
	AAEL007632	Myosin light chain kinase
	AAEL012926	Unconventional Myosin 95e isoform
	AAEL001068	Myosin light chain 2V, putative
	AAEL004750	Nonmuscle Myosin heavy chain-A, putative
	AAEL011428	Fast myosin heavy chain HCIII, putative

Gene family	Gene ID	Name/Description
	AAEL002572	Myosin regulatory light chain 2 (mlc-2)
	AAEL008921	Myosin regulatory light chain 2 smooth muscle
	AAEL001411	Myosin heavy chain, nonmuscle or smooth muscle
	AAEL005656	Myosin heavy chain, nonmuscle or smooth muscle
	AAEL001220	CK
**Heat shock proteins**	AAEL011708	Heat shock protein
	AAEL000301	Heat shock protein
	AAEL014843	Heat shock protein
	AAEL013161	Heat shock protein, putative
	AAEL001052	Heat shock protein, putative
	AAEL017976	Heat shock protein HSP70
	AAEL019403	Heat shock cognate 70
	AAEL017975	Heat shock protein HSP70
	AAEL013350	Heat shock protein 26kD, putative
	AAEL010546	Heat shock factor binding protein, putative
	AAEL004148	Heat shock protein 70 (hsp70)-interacting protein
	AAEL001952	28 kDa heat- and acid-stable phosphoprotein (PDGF-associated protein), putative
**Cell proliferation**	AAEL008069	NOTCH
	AAEL011396	DELTA
**Cytochrome P450**	AAEL009132	CYP6Y3
	AAEL014615	CYP9J23
	AAEL001292	CYP9M7
	AAEL014603	CYP9J30
	AAEL007816	CYP4D23
	AAEL002633	CYP9J31
	AAEL014613	CYP9J24
	AAEL009133	CYP6N14
	AAEL005700	CYP325X4
	AAEL003890	CYP
	AAEL014619	CYP9J22
	AAEL011463	CYP
	AAEL009591	CYP9M8
	AAEL006984	CYP6AG5
	AAEL008638	CYP49A1
	AAEL010151	CYP6N16
	AAEL007808	CYP4D39
	AAEL004941	CYP6AK1
	AAEL010158	CYP6N17
	AAEL002043	CYP305A5
	AAEL014610	CYP9J29

Gene family	Gene ID	Name/Description
	AAEL001960	CYP
	AAEL005771	CYP325K2
	AAEL017539	CYP6BY1
	AAEL005788	CYP325K3
	AAEL006992	CYP6AG6
	AAEL003763	CYP329B1
	AAEL004054	CYP4G36
	AAEL014605	CYP9J9
	AAEL003748	CYP9AE1
	AAEL019911	CYP325S2
	AAEL011850	CYP315A1
	AAEL014893	CYP6BB2
	AAEL009125	CYP6M10
	AAEL009128	CYP6M6
	AAEL017297	CYP6M9
	AAEL009762	CYP307A1
	AAEL009130	CYP6Z7
	AAEL014891	CYP6P12
	AAEL007807	CYP4D38
	AAEL012770	CYP325N1
	AAEL014594	CYP301A1
	AAEL006257	CYP325Y1
	AAEL007798	CYP4K3
	AAEL013554	CYP4J14
	AAEL002638	CYP9J6
	AAEL000357	CYP325S3
	AAEL009138	CYP6N11
	AAEL011761	CYP325M5
	AAEL004870	CYP18A1
	AAEL014411	CYP304B3
	AAEL009117	CYP6M5
	AAEL002005	CYP12F6
	AAEL003399	CYP4H30
	AAEL002031	CYP12F7
	AAEL012761	CYP325T2
	AAEL000320	CYP325T1
	AAEL009127	CYP6M11
	AAEL000340	CYP
	AAEL009129	CYP6Z9
	AAEL007812	CYP4H32
	AAEL005695	CYP325X1
	AAEL010154	CYP4AR2
	AAEL009124	CYP6N12
	AAEL007473	CYP6AH1
	AAEL007795	CYP4D37

Gene family	Gene ID	Name/Description
	AAEL009123	CYP6Z6
	AAEL012765	CYP325M3
	AAEL013556	CYP4J15
	AAEL001312	CYP9M6
	AAEL006784	CYP9J17
	AAEL011770	CYP325L1
	AAEL006827	CYP12F8
	AAEL009120	CYP6S3
	AAEL003380	CYP4H28
	AAEL009018	CYP
	AAEL005696	CYP325X2
	AAEL005775	CYP325R1
	AAEL018028	CYP325Y3
	AAEL014413	CYP304C1
	AAEL014614	CYP
	AAEL017136	CYP325V1
	AAEL014609	CYP9J26
	AAEL012766	CYP325G2
	AAEL017215	CYP325U1
	AAEL005006	CYP6CD1
	AAEL012266	CYP4C38
	AAEL009131	CYP6Z8
	AAEL012769	CYP325M2
	AAEL006824	CYP
	AAEL012772	CYP325G3
	AAEL014019	CYP4J16
	AAEL006815	CYP9J16
	AAEL007830	CYP4H29
	AAEL006795	CYP9J15
	AAEL009121	CYP6N9
	AAEL002085	CYP4H31
	AAEL012762	CYP325N2
	AAEL007010	CYP6AG4
	AAEL006989	CYP6AG7
	AAEL014618	CYP
	AAEL014830	CYP
	AAEL014678	CYP6F2
	AAEL010946	CYP314A1
	AAEL008889	CYP6AL1
	AAEL006805	CYP9J2
	AAEL009126	CYP6N6
	AAEL008018	CYP4C51
	AAEL014604	CYP
	AAEL013798	CYP4H33
	AAEL009122	CYP

Gene family	Gene ID	Name/Description
	AAEL000338	CYP325E3
	AAEL007815	CYP4D24
	AAEL000326	CYP325S1
	AAEL014684	CYP6F3
	AAEL009656	CYP6AL3
	AAEL001807	CYP9M9
	AAEL014412	CYP304B2
	AAEL006058	CYP325Q2
	AAEL001320	CYP9M4
	AAEL014924	CYP
	AAEL012144	CYP303A1
	AAEL019910	CYP325S2
	AAEL007024	CYP6AG3
	AAEL009137	CYP6N13
	AAEL014890	CYP6CC1
	AAEL008017	CYP4C50
	AAEL006044	CYP325Q1
	AAEL014208	CYP
	AAEL014617	CYP9J28
**Small Heat shock proteins**	AAEL013348	Lethal (2)essential for life protein, l2efl
	AAEL013344	Lethal (2)essential for life protein, l2efl
	AAEL013351	Lethal (2)essential for life protein, l2efl
	AAEL013338	Lethal (2)essential for life protein, l2efl
	AAEL013349	Lethal (2)essential for life protein, l2efl
	AAEL013352	Lethal (2)essential for life protein, l2efl
	AAEL013341	Lethal (2)essential for life protein, l2efl
	AAEL013346	Lethal (2)essential for life protein, l2efl
	AAEL013347	Lethal (2)essential for life protein, l2efl
	AAEL010659	Lethal (2)essential for life protein, l2efl
**Salivary proteins**	AAEL004407	Allergen, putative
	AAEL004199	Allergen
	AAEL003057	Allergen
	AAEL010235	Allergen
	AAEL000793	Venom allergen
	AAEL002693	Venom allergen
	AAEL005531	Venom allergen
	AAEL013406	Venom allergen
	AAEL011798	Allergen, putative
	AAEL005997	Allergen, putative
	AAEL006524	Venom allergen
	AAEL011797	Venom allergen
	AAEL002476	Venom allergen
	AAEL003053	Allergen, putative
	AAEL026620	Allergen, putative

Gene family	Gene ID	Name/Description
	AAEL002682	Venom allergen
	AAEL009239	Venom allergen
	AAEL027045	Allergen, putative
	AAEL010269	Venom allergen
	AAEL026087	D7
	AAEL006347	APY
	AAEL002726	D7 protein, putative
	AAEL008620	D7 protein, putative
	AAEL006424	D7
	AAEL006417	D7 protein
**Lachesin**	AAEL009295	Lachesin
	AAEL004992	Lachesin, putative
	AAEL014334	Lachesin, putative
	AAEL006478	Lachesin, putative
	AAEL003966	Lachesin
	AAEL000576	Lachesin
**Sidestep proteins**	AAEL001227	Sidestep protein
	AAEL008010	Sidestep protein
	AAEL011989	Sidestep protein
	AAEL019917	Sidestep protein
	AAEL022485	Sidestep protein
	AAEL023141	Sidestep protein
**Vacuolar ATPase**	AAEL000291	Vacuolar ATPases
	AAEL008787	Vacuolar ATPases
	AAEL012113	Vacuolar ATPases
	AAEL011025	Vacuolar ATPases
	AAEL005798	Vacuolar ATPases
	AAEL015594	Vacuolar ATPases
	AAEL012035	Vacuolar ATPases
	AAEL013302	Vacuolar ATPases
	AAEL007184	Vacuolar ATPases
	AAEL012819	Vacuolar ATPases
	AAEL006516	Vacuolar ATPases
	AAEL010819	Vacuolar ATPases
	AAEL014053	Vacuolar ATPases
	AAEL003743	Vacuolar ATPases

Supplementary Table 7. Percent contribution of various gene ontologies across the temperatures

	% contribution at each temperature					
Cellular locations	3 dpi 18 ºC upregulated	3 dpi 18 ºC downregulated	3 dpi 28 ºC upregulated	3 dpi 28 ºC downregulated	3 dpi 32 ºC upregulated	3 dpi 32 ºC downregulated
GO:0044464 cell part	11.5	26.9	44.2	13.5	3.8	0.0
GO:0005623 cell	11.3	26.4	45.3	13.2	3.8	0.0
GO:0043226 organelle	10.0	30.0	33.3	20.0	6.7	0.0
GO:0032991 protein-containing complex	10.0	40.0	40.0	10.0	0.0	0.0
GO:0044422 organelle part	16.7	50.0	16.7	16.7	0.0	0.0
GO:0005576 extracellular region	10.3	20.7	69.0	0.0	0.0	0.0
GO:0044421 extracellular region part	14.3	7.1	78.6	0.0	0.0	0.0
GO:0044425 membrane part	17.0	12.3	65.1	1.9	2.8	0.9
GO:0016020 membrane	16.4	11.8	66.4	1.8	2.7	0.9
GO:0030054 cell junction	33.3	33.3	33.3	0.0	0.0	0.0
GO:0045202 synapse	33.3	33.3	33.3	0.0	0.0	0.0
GO:0031974 membrane-enclosed lumen	0.0	100.0	0.0	0.0	0.0	0.0
GO:0044456 synapse part	0.0	100.0	0.0	0.0	0.0	0.0

% contribution of DEGs for each gene ontology = No. of DEGs correspond to each annotation at each temperature/ total number of DEGs correspond to each annotation within 3 dpi*100

Cellular locations	7 dpi 18 ºC upregulated	7 dpi 18 ºC downregulated	7 dpi 28 ºC upregulated	7 dpi 28 ºC downregulated	7 dpi 32 ºC upregulated	7 dpi 32 ºC downregulated
GO:0044464 cell part	17.9	0.0	53.8	5.1	17.9	5.1
GO:0005623 cell	17.1	0.0	53.7	4.9	19.5	4.9
GO:0043226 organelle	23.8	0.0	57.1	9.5	4.8	4.8
GO:0044422 organelle part	33.3	0.0	66.7	0.0	0.0	0.0
GO:0032991 protein-containing complex	33.3	0.0	33.3	0.0	33.3	0.0
GO:0044425 membrane part	9.3	0.0	60.5	2.3	27.1	0.8
GO:0016020 membrane	9.0	0.0	60.4	2.2	27.6	0.7
GO:0044421 extracellular region part	9.1	0.0	63.6	0.0	27.3	0.0
GO:0005576 extracellular region	4.0	8.0	60.0	0.0	28.0	0.0
GO:0099080 supramolecular complex	0.0	0.0	100.0	0.0	0.0	0.0
GO:0044456 synapse part	0.0	0.0	100.0	0.0	0.0	0.0
GO:0045202 synapse	0.0	0.0	100.0	0.0	0.0	0.0
GO:0030054 cell junction	0.0	0.0	100.0	0.0	0.0	0.0

% contribution of various gene ontologies across the temperatures = No of DEGs correspond to each annotation at each temperature/ total number of DEGs correspond to each annotation within 7 dpi*100

	3 dpi 18 ºC upregulated	3 dpi 18 ºC downregulated	3 dpi 28 ºC upregulated	3 dpi 28 ºC downregulated	3 dpi 32 ºC upregulated	3 dpi 32 ºC downregulated
GO:0003824 catalytic activity	17.8	25.7	50.7	2.6	2.0	1.3
GO:0005215 transporter activity	12.9	9.7	74.2	0.0	3.2	0.0
GO:0005488 binding	15.0	25.2	49.7	8.8	0.0	1.4
GO:0140110 transcription regulator activity	27.8	5.6	61.1	5.6	0.0	0.0
GO:0060089 molecular transducer activity	25.0	25.0	50.0	0.0	0.0	0.0
GO:0005198 structural molecule activity	33.3	0.0	66.7	0.0	0.0	0.0
GO:0038024 cargo receptor activity	0.0	0.0	100.0	0.0	0.0	0.0
GO:0098772 molecular function regulator	0.0	0.0	100.0	0.0	0.0	0.0
GO:0045735 nutrient reservoir activity	0.0	0.0	100.0	0.0	0.0	0.0
GO:0016209 antioxidant activity	0.0	0.0	0.0	100.0	0.0	0.0

% contribution of various gene ontologies across the temperatures = No of DEGs correspond to each annotation at each temperature/ total number of DEGs correspond to each annotation within 3 dpi*100

Molecular functions	7 dpi 18 ºC upregulated	7 dpi 18 ºC downregulated	7 dpi 28 ºC upregulated	7 dpi 28 ºC downregulated	7 dpi 32 ºC upregulated	7 dpi 32 ºC downregulated
GO:0003824 catalytic activity	5.2	1.7	58.7	3.5	30.2	0.6
GO:0005488 binding	10.4	2.2	54.5	9.0	20.9	3.0
GO:0005215 transporter activity	2.5	0.0	62.5	0.0	35.0	0.0
GO:0005198 structural molecule activity	14.3	0.0	57.1	0.0	28.6	0.0
GO:0140110 transcription regulator activity	26.3	0.0	63.2	0.0	10.5	0.0
GO:0098772 molecular function regulator	11.1	0.0	66.7	0.0	22.2	0.0
GO:0060089 molecular transducer activity	33.3	0.0	33.3	0.0	0.0	33.3
GO:0038024 cargo receptor activity	0.0	0.0	100.0	0.0	0.0	0.0
GO:0045735 nutrient reservoir activity	0.0	0.0	50.0	0.0	50.0	0.0

% contribution of various gene ontologies across the temperatures = No of DEGs correspond to each annotation at each temperature/ total number of DEGs correspond to each annotation within 7 dpi*100

Biological functions	3 dpi 18 ºC upregulated	3 dpi 18 ºC downregulated	3 dpi 28 ºC upregulated	3 dpi 28 ºC downregulated	3 dpi 32 ºC upregulated	3 dpi 32 ºC downregulated
GO:0051179 localization	37.5	12.5	50.0	0.0	0.0	0.0
GO:0008152 metabolic process	16.7	29.2	47.2	5.6	1.4	0.0
GO:0009987 cellular process	19.5	22.1	49.4	6.5	2.6	0.0
GO:0065007 biological regulation	18.5	14.8	55.6	3.7	7.4	0.0
GO:0050789 regulation of biological process	20.8	12.5	54.2	4.2	8.3	0.0
GO:0023052 signaling	14.3	7.1	71.4	7.1	0.0	0.0
GO:0050896 response to stimulus	20.0	8.0	56.0	8.0	8.0	0.0
GO:0022610 biological adhesion	20.0	20.0	60.0	0.0	0.0	0.0
GO:0048519 negative regulation of biological process	100.0	0.0	0.0	0.0	0.0	0.0
GO:0007610 behavior	0.0	100.0	0.0	0.0	0.0	0.0
GO:0032501 multicellular organismal process	0.0	20.0	80.0	0.0	0.0	0.0
GO:0002376 immune system process	0.0	20.0	80.0	0.0	0.0	0.0
GO:0071840 cellular component organization or biogenesis	0.0	0.0	100.0	0.0	0.0	0.0
GO:0051704 multi-organism process	0.0	0.0	100.0	0.0	0.0	0.0
GO:0040007 growth	0.0	0.0	100.0	0.0	0.0	0.0
GO:0032502 developmental process	0.0	0.0	100.0	0.0	0.0	0.0
GO:0008283 cell proliferation	0.0	0.0	0.0	100.0	0.0	0.0

% contribution of various gene ontologies across the temperatures = No of DEGs correspond to each annotation at each temperature/ total number of DEGs correspond to each annotation within 3 dpi*100

Biological functions	7 dpi 18 ºC upregulated	7 dpi 18 ºC downregulated	7 dpi 28 ºC upregulated	7 dpi 28 ºC downregulated	7 dpi 32 ºC upregulated	7 dpi 32 ºC downregulated
GO:0008152 metabolic process	6.2	1.5	58.5	6.2	24.6	3.1
GO:0051179 localization	5.6	5.6	55.6	0.0	33.3	0.0
GO:0032501 multicellular organismal process	16.7	0.0	50.0	0.0	16.7	16.7
GO:0071840 cellular component organization or biogenesis	25.0	0.0	25.0	0.0	25.0	25.0
GO:0009987 cellular process	10.1	1.4	59.4	5.8	18.8	4.3
GO:0065007 biological regulation	12.0	0.0	52.0	12.0	20.0	4.0
GO:0050789 regulation of biological process	14.3	0.0	47.6	14.3	19.0	4.8
GO:0023052 signaling	9.1	0.0	45.5	9.1	27.3	9.1
GO:0050896 response to stimulus	11.8	0.0	47.1	5.9	29.4	5.9
GO:0048519 negative regulation of biological process	0.0	0.0	100.0	0.0	0.0	0.0
GO:0032502 developmental process	0.0	0.0	66.7	0.0	33.3	0.0
GO:0022610 biological adhesion	0.0	0.0	33.3	0.0	66.7	0.0
GO:0048511 rhythmic process	0.0	0.0	100.0	0.0	0.0	0.0
GO:0002376 immune system process	0.0	0.0	100.0	0.0	0.0	0.0
GO:0000003 reproduction	0.0	0.0	100.0	0.0	0.0	0.0
GO:0051704 multi-organism process	0.0	0.0	66.7	0.0	33.3	0.0
GO:0022414 reproductive process	0.0	0.0	100.0	0.0	0.0	0.0
GO:0048518 positive regulation of biological process	0.0	0.0	0.0	100.0	0.0	0.0
GO:0040007 growth	0.0	0.0	0.0	0.0	100.0	0.0

% contribution of various gene ontologies across the temperatures = No of DEGs correspond to each annotation at each temperature/ total number of DEGs correspond to each annotation within 7 dpi*100

Supplementary Table 8. Percent contribution of various pathways across the temperatures

KEGG Pathway ID	% contribution at each temperature*					
	3 dpi 18 ºC upregulated	3 dpi 18 ºC downregulated	3 dpi 28 ºC upregulated	3 dpi 28 ºC downregulated	3 dpi 32 ºC upregulated	3 dpi 32 ºC downregulated
aag00010:Glycolysis / Gluconeogenesis	0.0	0.0	50.0	50.0	0.0	0.0
aag00040:Pentose and glucuronate interconversions	0.0	0.0	0.0	0.0	0.0	100.0
aag00051:Fructose and mannose metabolism	0.0	0.0	50.0	0.0	0.0	50.0
aag00052:Galactose metabolism	0.0	50.0	50.0	0.0	0.0	0.0
aag00053:Ascorbate and aldarate metabolism	0.0	100.0	0.0	0.0	0.0	0.0
aag00220:Arginine biosynthesis	33.3	33.3	33.3	0.0	0.0	0.0
aag00230:Purine metabolism	0.0	0.0	75.0	25.0	0.0	0.0
aag00240:Pyrimidine metabolism	0.0	0.0	50.0	50.0	0.0	0.0
aag00250:Alanine, aspartate and glutamate metabolism	50.0	0.0	50.0	0.0	0.0	0.0
aag00260:Glycine, serine and threonine metabolism	0.0	60.0	40.0	0.0	0.0	0.0
aag00270:Cysteine and methionine metabolism	0.0	100.0	0.0	0.0	0.0	0.0
aag00330:Arginine and proline metabolism	0.0	50.0	50.0	0.0	0.0	0.0
aag00380:Tryptophan metabolism	0.0	0.0	100.0	0.0	0.0	0.0
aag00472:D-Arginine and D-ornithine metabolism	0.0	0.0	100.0	0.0	0.0	0.0
aag00480:Glutathione metabolism	0.0	0.0	100.0	0.0	0.0	0.0
aag00500:Starch and sucrose metabolism	0.0	50.0	50.0	0.0	0.0	0.0
aag00511:Other glycan degradation	0.0	100.0	0.0	0.0	0.0	0.0
aag00520:Amino sugar and nucleotide sugar metabolism	0.0	75.0	25.0	0.0	0.0	0.0
aag00531:Glycosaminoglycan degradation	0.0	100.0	0.0	0.0	0.0	0.0
aag00561:Glycerolipid metabolism	0.0	0.0	100.0	0.0	0.0	0.0

KEGG Pathway ID	% contribution at each temperature*					
	3 dpi 18 ºC upregulated	3 dpi 18 ºC downregulated	3 dpi 28 ºC upregulated	3 dpi 28 ºC downregulated	3 dpi 32 ºC upregulated	3 dpi 32 ºC downregulated
aag00562:Inositol phosphate metabolism	33.3	33.3	0.0	33.3	0.0	0.0
aag00564:Glycerophospholipid metabolism	0.0	0.0	100.0	0.0	0.0	0.0
aag00565:Ether lipid metabolism	0.0	0.0	100.0	0.0	0.0	0.0
aag00600:Sphingolipid metabolism	0.0	50.0	50.0	0.0	0.0	0.0
aag00603:Glycosphingolipid biosynthesis - globo series	0.0	100.0	0.0	0.0	0.0	0.0
aag00604:Glycosphingolipid biosynthesis - ganglio series	0.0	100.0	0.0	0.0	0.0	0.0
aag00630:Glyoxylate and dicarboxylate metabolism	0.0	100.0	0.0	0.0	0.0	0.0
aag00670:One carbon pool by folate	0.0	100.0	0.0	0.0	0.0	0.0
aag00730:Thiamine metabolism	0.0	100.0	0.0	0.0	0.0	0.0
aag00760:Nicotinate and nicotinamide metabolism	0.0	0.0	100.0	0.0	0.0	0.0
aag00900:Terpenoid backbone biosynthesis	0.0	100.0	0.0	0.0	0.0	0.0
aag00981:Insect hormone biosynthesis	0.0	20.0	80.0	0.0	0.0	0.0
aag01100:Metabolic pathways	12.0	36.0	40.0	8.0	0.0	4.0
aag01130:Biosynthesis of antibiotics	12.5	37.5	50.0	0.0	0.0	0.0
aag01200:Carbon metabolism	0.0	50.0	50.0	0.0	0.0	0.0
aag01230:Biosynthesis of amino acids	25.0	25.0	50.0	0.0	0.0	0.0
aag02010:ABC transporters	0.0	0.0	100.0	0.0	0.0	0.0
aag03013:RNA transport	0.0	50.0	0.0	50.0	0.0	0.0
aag03015:mRNA surveillance pathway	0.0	100.0	0.0	0.0	0.0	0.0
aag03018:RNA degradation	0.0	50.0	50.0	0.0	0.0	0.0
aag03030:DNA replication	0.0	0.0	0.0	100.0	0.0	0.0
aag03040:Spliceosome	0.0	0.0	0.0	100.0	0.0	0.0
aag03410:Base excision repair	0.0	0.0	0.0	100.0	0.0	0.0
aag03420:Nucleotide excision repair	0.0	0.0	0.0	100.0	0.0	0.0

KEGG Pathway ID	% contribution at each temperature*					
	3 dpi 18 ºC upregulated	3 dpi 18 ºC downregulated	3 dpi 28 ºC upregulated	3 dpi 28 ºC downregulated	3 dpi 32 ºC upregulated	3 dpi 32 ºC downregulated
aag04068:FoxO signaling pathway	50.0	0.0	50.0	0.0	0.0	0.0
aag04068:FoxO signaling pathway,	0.0	0.0	0.0	0.0	100.0	0.0
aag04070:Phosphatidylinositol signaling system	100.0	0.0	0.0	0.0	0.0	0.0
aag04080:Neuroactive ligand-receptor interaction	0.0	66.7	33.3	0.0	0.0	0.0
aag04120:Ubiquitin mediated proteolysis	0.0	0.0	100.0	0.0	0.0	0.0
aag04141:Protein processing in endoplasmic reticulum	62.5	0.0	37.5	0.0	0.0	0.0
aag04142:Lysosome	0.0	33.3	66.7	0.0	0.0	0.0
aag04145:Phagosome	0.0	50.0	50.0	0.0	0.0	0.0
aag04146:Peroxisome	0.0	50.0	50.0	0.0	0.0	0.0
aag04310:Wnt signaling pathway	50.0	0.0	50.0	0.0	0.0	0.0
aag04320:Dorso-ventral axis formation	0.0	0.0	100.0	0.0	0.0	0.0
aag04340:Hedgehog signaling pathway	0.0	0.0	100.0	0.0	0.0	0.0
aag04350:TGF-beta signaling pathway	0.0	0.0	100.0	0.0	0.0	0.0
aag04391:Hippo signaling pathway - fly	20.0	0.0	60.0	20.0	0.0	0.0
aag04512:ECM-receptor interaction	0.0	50.0	50.0	0.0	0.0	0.0
aag04711:Circadian rhythm - fly	33.3	33.3	33.3	0.0	0.0	0.0
aag04745:Phototransduction - fly	0.0	0.0	100.0	0.0	0.0	0.0
aag04931:Insulin resistance	50.0	0.0	50.0	0.0	0.0	0.0

*% contribution at each temperature= No. of DEGs at each temperature/ Total number of DEGs at all temperatures within a given dpi

7 dpi

KEGG Pathway ID	% contribution at each temperature*					
	**7 dpi 18 ºC upregulated**	**7 dpi 18 ºC downregulated**	**7 dpi 28 ºC upregulated**	**7 dpi 28 ºC downregulated**	**7 dpi 32 ºC upregulated**	**7 dpi 32 ºC downregulated**
aag00010:Glycolysis / Gluconeogenesis	0.0	0.0	100.0	0.0	0.0	0.0
aag00020:Citrate cycle (TCA cycle)	0.0	0.0	100.0	0.0	0.0	0.0
aag00040:Pentose and glucuronate interconversions	0.0	0.0	33.3	0.0	66.7	0.0
aag00051:Fructose and mannose metabolism	0.0	0.0	100.0	0.0	0.0	0.0
aag00052:Galactose metabolism	0.0	0.0	100.0	0.0	0.0	0.0
aag00053:Ascorbate and aldarate metabolism	0.0	0.0	0.0	0.0	100.0	0.0
aag00061:Fatty acid biosynthesis	0.0	0.0	100.0	0.0	0.0	0.0
aag00071:Fatty acid degradation	0.0	0.0	100.0	0.0	0.0	0.0
aag00130:Ubiquinone and other terpenoid-quinone biosynthesis	0.0	0.0	100.0	0.0	0.0	0.0
aag00190:Oxidative phosphorylation	0.0	0.0	100.0	0.0	0.0	0.0
aag00220:Arginine biosynthesis	0.0	0.0	66.7	0.0	33.3	0.0
aag00230:Purine metabolism	0.0	0.0	50.0	0.0	50.0	0.0
aag00232:Caffeine metabolism	0.0	0.0	100.0	0.0	0.0	0.0
aag00250:Alanine, aspartate and glutamate metabolism	0.0	0.0	66.7	0.0	33.3	0.0
aag00260:Glycine, serine and threonine metabolism	0.0	0.0	40.0	0.0	60.0	0.0
aag00270:Cysteine and methionine metabolism	0.0	0.0	100.0	0.0	0.0	0.0
aag00330:Arginine and proline metabolism	0.0	0.0	50.0	0.0	50.0	0.0
aag00350:Tyrosine metabolism	0.0	0.0	100.0	0.0	0.0	0.0
aag00360:Phenylalanine metabolism	0.0	0.0	100.0	0.0	0.0	0.0
aag00400:Phenylalanine, tyrosine and tryptophan biosynthesis	0.0	0.0	100.0	0.0	0.0	0.0
aag00480:Glutathione metabolism	0.0	0.0	50.0	0.0	50.0	0.0
aag00500:Starch and sucrose metabolism	0.0	0.0	100.0	0.0	0.0	0.0
aag00511:Other glycan degradation	0.0	0.0	100.0	0.0	0.0	0.0

KEGG Pathway ID	% contribution at each temperature*					
	7 dpi 18 ºC upregulated	7 dpi 18 ºC downregulated	7 dpi 28 ºC upregulated	7 dpi 28 ºC downregulated	7 dpi 32 ºC upregulated	7 dpi 32 ºC downregulated
aag00514:Other types of O-glycan biosynthesis	0.0	0.0	100.0	0.0	0.0	0.0
aag00520:Amino sugar and nucleotide sugar metabolism	0.0	0.0	100.0	0.0	0.0	0.0
aag00534:Glycosaminoglycan biosynthesis - heparan sulfate / heparin	0.0	0.0	0.0	100.0	0.0	0.0
aag00564:Glycerophospholipid metabolism	0.0	0.0	0.0	0.0	100.0	0.0
aag00565:Ether lipid metabolism	0.0	0.0	0.0	0.0	100.0	0.0
aag00620:Pyruvate metabolism	0.0	0.0	100.0	0.0	0.0	0.0
aag00630:Glyoxylate and dicarboxylate metabolism	0.0	0.0	66.7	0.0	33.3	0.0
aag00830:Retinol metabolism	0.0	0.0	0.0	0.0	100.0	0.0
aag00860:Porphyrin and chlorophyll metabolism	0.0	0.0	0.0	0.0	100.0	0.0
aag00910:Nitrogen metabolism	0.0	0.0	100.0	0.0	0.0	0.0
aag00970:Aminoacyl-tRNA biosynthesis	0.0	0.0	0.0	0.0	0.0	100.0
aag00980:Metabolism of xenobiotics by cytochrome P450	0.0	0.0	0.0	0.0	100.0	0.0
aag00981:Insect hormone biosynthesis	0.0	0.0	66.7	0.0	33.3	0.0
aag00982:Drug metabolism - cytochrome P450	0.0	0.0	0.0	0.0	100.0	0.0
aag00983:Drug metabolism - other enzymes	0.0	0.0	50.0	0.0	50.0	0.0
aag01040:Biosynthesis of unsaturated fatty acids	0.0	0.0	100.0	0.0	0.0	0.0
aag01100:Metabolic pathways	0.0	0.0	60.9	0.0	39.1	0.0
aag01130:Biosynthesis of antibiotics	0.0	0.0	72.7	0.0	27.3	0.0
aag01200:Carbon metabolism	0.0	0.0	80.0	0.0	20.0	0.0
aag01212:Fatty acid metabolism	0.0	0.0	100.0	0.0	0.0	0.0
aag01230:Biosynthesis of amino acids	0.0	0.0	75.0	0.0	25.0	0.0
aag02010:ABC transporters	0.0	0.0	66.7	0.0	33.3	0.0
aag03013:RNA transport	0.0	0.0	100.0	0.0	0.0	0.0
aag03015:mRNA surveillance pathway	0.0	0.0	0.0	0.0	100.0	0.0
aag03022:Basal transcription factors	0.0	0.0	0.0	100.0	0.0	0.0

KEGG Pathway ID	% contribution at each temperature*					
	7 dpi 18 ºC upregulated	7 dpi 18 ºC downregulated	7 dpi 28 ºC upregulated	7 dpi 28 ºC downregulated	7 dpi 32 ºC upregulated	7 dpi 32 ºC downregulated
aag04068:FoxO signaling pathway	28.6	0.0	28.6	0.0	28.6	14.3
aag04080:Neuroactive ligand-receptor interaction	100.0	0.0	0.0	0.0	0.0	0.0
aag04120:Ubiquitin mediated proteolysis	0.0	0.0	0.0	0.0	0.0	100.0
aag04140:Regulation of autophagy	0.0	0.0	0.0	0.0	100.0	0.0
aag04141:Protein processing in endoplasmic reticulum	33.3	0.0	33.3	0.0	33.3	0.0
aag04142:Lysosome	0.0	0.0	75.0	0.0	25.0	0.0
aag04144:Endocytosis	0.0	100.0	0.0	0.0	0.0	0.0
aag04145:Phagosome	100.0	0.0	0.0	0.0	0.0	0.0
aag04146:Peroxisome	0.0	0.0	62.5	12.5	25.0	0.0
aag04310:Wnt signaling pathway	28.6	0.0	42.9	0.0	28.6	0.0
aag04320:Dorso-ventral axis formation	0.0	0.0	100.0	0.0	0.0	0.0
aag04391:Hippo signaling pathway - fly	33.3	0.0	33.3	16.7	16.7	0.0
aag04512:ECM-receptor interaction	0.0	0.0	100.0	0.0	0.0	0.0
aag04711:Circadian rhythm - fly	33.3	0.0	66.7	0.0	0.0	0.0
aag04745:Phototransduction - fly	100.0	0.0	0.0	0.0	0.0	0.0
aag04931:Insulin resistance	20.0	0.0	40.0	20.0	20.0	0.0

*% contribution at each temperature= No. of DEGs at each temperature/ Total number of DEGs at all temperatures within a given dpi

Supplementary Table 9. Genes unmapped to DAVID cloud map.

3 dpi 18 ºC upregulated	3 dpi 18 ºC downregulated	3 dpi 28 ºC upregulated	3 dpi 28 ºC upregulated	3 dpi 28 ºC upregulated
AAEL020330	AAEL024221	AAEL025079	AAEL028635	AAEL027610
AAEL019751	AAEL021086	AAEL020330	AAEL027655	AAEL022876
AAEL027238	AAEL019650	AAEL019578	AAEL021257	AAEL027362
AAEL023745	AAEL021576	AAEL029047	AAEL027493	AAEL020033
AAEL018301	AAEL024146	AAEL026440	AAEL026008	AAEL026843
AAEL018304	AAEL023395	AAEL025125	AAEL024175	AAEL027514
AAEL026878	AAEL019698	AAEL024669	AAEL026300	
AAEL023999	AAEL021513	AAEL022059	AAEL026031	
AAEL022900	AAEL024475	AAEL027093	AAEL024540	
AAEL026300	AAEL023431	AAEL020078	AAEL022600	
AAEL025532	AAEL019834	AAEL025894	AAEL024112	
AAEL026868	AAEL024926	AAEL022674	AAEL028247	
AAEL021302	AAEL021138	AAEL027106	AAEL026819	
AAEL023746	AAEL023844	AAEL021929	AAEL022427	
AAEL022253	AAEL018216	AAEL027700	AAEL026447	
AAEL024512	AAEL024064	AAEL019773	AAEL026833	
AAEL022363	AAEL023158	AAEL024838	AAEL026537	
AAEL024540	AAEL023753	AAEL026751	AAEL026041	
AAEL025894	AAEL020477	AAEL025126	AAEL029046	
AAEL019935	AAEL024122	AAEL026175	AAEL025750	
AAEL021595	AAEL028236	AAEL018351	AAEL019849	
AAEL023591	AAEL027166	AAEL025392	AAEL018241	
AAEL027610	AAEL018125	AAEL022387	AAEL019438	
AAEL018241	AAEL024149	AAEL027699	AAEL027829	
AAEL026519	AAEL025667	AAEL023395	AAEL026603	
AAEL025531	AAEL026023	AAEL019537	AAEL023729	
AAEL017975	AAEL019588	AAEL019528	AAEL025332	
AAEL021614	AAEL025199	AAEL021072	AAEL022079	
AAEL026194	AAEL023874	AAEL023882	AAEL023999	
AAEL020777	AAEL024161	AAEL021302	AAEL021795	
AAEL026751	AAEL021180	AAEL027019	AAEL025839	
AAEL017976	AAEL028002	AAEL020603	AAEL023560	
AAEL020575		AAEL022829	AAEL027270	
AAEL025126		AAEL025552	AAEL029031	
AAEL019637		AAEL019868	AAEL026215	
AAEL019995		AAEL019844	AAEL019751	
AAEL028247		AAEL017975	AAEL028221	
AAEL020957		AAEL020340	AAEL023348	
AAEL026833		AAEL021016	AAEL027188	
AAEL023321		AAEL019893	AAEL021583	
AAEL022079		AAEL024560	AAEL027632	
AAEL018189		AAEL022334	AAEL019463	
AAEL020236		AAEL019564	AAEL017976	
AAEL026215		AAEL024038	AAEL024512	
AAEL022059		AAEL025531	AAEL023321	
AAEL024560		AAEL021982	AAEL026531	
AAEL019902		AAEL019767	AAEL023591	
AAEL027096		AAEL022253	AAEL019684	
AAEL026008		AAEL024468	AAEL023243	

3 dpi 28 ºC downregulated	3 dpi 32 ºC upregulated	3 dpi 32 ºC downregulated
AAEL019677	AAEL020330	AAEL024207
AAEL024298	AAEL021012	
AAEL022506	AAEL024560	
AAEL026775	AAEL023321	
AAEL028022	AAEL017976	
AAEL026260		
AAEL018189		
AAEL025401		
AAEL024022		
AAEL020175		
AAEL026241		
AAEL021303		
AAEL022382		
AAEL025549		
AAEL022900		

7 dpi 18 ºC upregulated	7 dpi 18 ºC downregulated	7 dpi 28 ºC upregulated	7 dpi 28 ºC upregulated	7 dpi 28 ºC downregulated
AAEL026008	AAEL025334	AAEL019955	AAEL024337	AAEL019889
AAEL022059	AAEL018189	AAEL027610	AAEL028635	AAEL019885
AAEL022253	AAEL023617	AAEL020330	AAEL021929	AAEL024757
AAEL025332	AAEL020035	AAEL021257	AAEL026214	AAEL018234
AAEL024597	AAEL029039	AAEL019658	AAEL025531	AAEL024284
AAEL024257		AAEL023524	AAEL026603	AAEL020075
AAEL026833		AAEL024512	AAEL019463	AAEL026107
AAEL021072		AAEL020706	AAEL026833	AAEL021576
AAEL020330		AAEL026481	AAEL018334	AAEL021308
AAEL019704		AAEL022059	AAEL020936	AAEL019629
AAEL020804		AAEL024269	AAEL027545	AAEL022124
AAEL023321		AAEL025921	AAEL025126	AAEL021449
AAEL019751		AAEL020502	AAEL022775	AAEL024830
AAEL019899		AAEL029061	AAEL026300	
AAEL028247		AAEL027700	AAEL024717	
AAEL017976		AAEL026175	AAEL021555	
AAEL019684		AAEL019537	AAEL019751	
AAEL027802		AAEL024406	AAEL018340	
AAEL017975		AAEL019527	AAEL022253	
AAEL022079		AAEL027068	AAEL022932	
AAEL023002		AAEL021073	AAEL024753	
AAEL023591		AAEL024838	AAEL023125	
AAEL024512		AAEL025296	AAEL018150	
AAEL024038		AAEL019487	AAEL021375	
AAEL019688		AAEL021016	AAEL022593	
AAEL020028		AAEL025177	AAEL017976	
AAEL022829		AAEL026446	AAEL018343	
AAEL023395		AAEL029031	AAEL022982	
AAEL026848		AAEL022387	AAEL020392	
AAEL025731		AAEL021795	AAEL027984	
AAEL024887		AAEL028228	AAEL019752	
AAEL020092		AAEL022578	AAEL021302	
AAEL025894		AAEL023100	AAEL023294	
AAEL023555		AAEL028247	AAEL025903	
AAEL026031		AAEL019564	AAEL023844	
AAEL029030		AAEL019847	AAEL022829	
AAEL027610		AAEL021583	AAEL023847	
		AAEL026843	AAEL019917	
		AAEL024291	AAEL026751	
		AAEL023591	AAEL024583	
		AAEL023321	AAEL022079	
		AAEL024540	AAEL017975	
		AAEL024216		
		AAEL019954		
		AAEL026008		
		AAEL022674		
		AAEL021099		
		AAEL020430		
		AAEL027188		
		AAEL021263		

7 dpi 32ºC upregulated	7 dpi 32ºC downregulated
AAEL020330	AAEL025193
AAEL027157	AAEL023039
AAEL027610	AAEL022225
AAEL023509	AAEL025401
AAEL022253	AAEL018041
AAEL019504	AAEL020306
AAEL028247	AAEL021011
AAEL019590	AAEL024449
AAEL023844	AAEL026029
AAEL026819	AAEL020913
AAEL024560	AAEL019689
AAEL023591	AAEL026468
AAEL018159	AAEL024183
AAEL027654	AAEL018184
AAEL025574	
AAEL020502	
AAEL022334	
AAEL018103	
AAEL022208	
AAEL019641	
AAEL019564	
AAEL026161	
AAEL022059	
AAEL026537	
AAEL017975	
AAEL017976	
AAEL027362	
AAEL022589	
AAEL019751	
AAEL023999	
AAEL021929	
AAEL021302	
AAEL022079	
AAEL027829	
AAEL022982	
AAEL024179	
AAEL026008	
AAEL019602	
AAEL026390	
AAEL025126	
AAEL026496	

Supplementary Table 10. LncRNA

3 dpi 18 ºC upregulated	3 dpi 18 ºC downregulated	3 dpi 28 ºC upregulated	3 dpi 28 ºC downregulated
AAEL019751	AAEL021086	AAEL025079	AAEL026775
AAEL027238	AAEL021576	AAEL019578	AAEL026260
AAEL023999	AAEL024146	AAEL027093	AAEL018189
AAEL022900	AAEL023395	AAEL025894	AAEL020175
AAEL025532	AAEL019698	AAEL027106	AAEL022382
AAEL026868	AAEL021513	AAEL021929	AAEL025549
AAEL021302	AAEL023431	AAEL027700	AAEL022900
AAEL024540	AAEL024926	AAEL019773	
AAEL025894	AAEL021138	AAEL025126	
AAEL026519	AAEL023158	AAEL023395	
AAEL021614	AAEL024122	AAEL019528	
AAEL020777	AAEL024149	AAEL021072	
AAEL020575	AAEL024161	AAEL023882	
AAEL025126		AAEL021302	
AAEL022079		AAEL022829	
AAEL018189		AAEL025552	
		AAEL019844	
		AAEL020340	
		AAEL022334	
		AAEL024038	
		AAEL021982	
		AAEL027655	
		AAEL027493	
		AAEL024540	
		AAEL024112	
		AAEL026447	
		AAEL026537	
		AAEL026603	
		AAEL025332	
		AAEL022079	
		AAEL023999	
		AAEL027270	
		AAEL019751	
		AAEL021583	
		AAEL022876	
		AAEL027362	

3 dpi 32ºC upregulated, 3 dpi 32ºC downregulated
None

7 dpi 18 ºC upregulated	7 dpi 18 ºC downregulated	7 dpi 28 ºC upregulated	7 dpi 28 ºC downregulated	7 dpi 32 ºC upregulated	7 dpi 32 ºC downregulated
AAEL025332	AAEL018189	AAEL026481	AAEL026107	AAEL022334	AAEL025193
AAEL024257		AAEL024269	AAEL021576	AAEL022208	AAEL023039
AAEL021072		AAEL027700	AAEL022124	AAEL026161	AAEL020306
AAEL019704		AAEL025296		AAEL026537	AAEL026029
AAEL019751		AAEL023100		AAEL027362	AAEL024183
AAEL022079		AAEL021583		AAEL022589	
AAEL024038		AAEL024540		AAEL019751	
AAEL020028		AAEL019954		AAEL023999	
AAEL022829		AAEL021929		AAEL021929	
AAEL023395		AAEL026603		AAEL021302	
AAEL024887		AAEL018334		AAEL022079	
AAEL025894		AAEL025126		AAEL026390	
		AAEL019751		AAEL025126	
		AAEL022932			
		AAEL019752			
		AAEL021302			
		AAEL025903			
		AAEL022829			
		AAEL023847			
		AAEL019917			
		AAEL022079			
